# Selective RNA sequestration in biomolecular condensates directs cell fate transitions

**DOI:** 10.1038/s41587-025-02853-z

**Published:** 2025-10-28

**Authors:** Patrizia Pessina, Mika Nevo, Junchao Shi, Srikanth Kodali, Eduard Casas, Yingzhi Cui, Alicia L. Richards, Emily J. Park, Xi Chen, Florencia Levin-Ferreyra, Alejandra Rivera Tostado, Erica Stevenson, Nevan J. Krogan, Danielle L. Swaney, Qilong Ying, Qi Chen, Justin Brumbaugh, Bruno Di Stefano

**Affiliations:** 1 Stem Cells and Regenerative Medicine Center, Baylor College of Medicine, Houston, TX USA; 2 Center for Cell and Gene Therapy, Baylor College of Medicine, Houston, TX USA; 3 Department of Molecular and Cellular Biology, Baylor College of Medicine, Houston, TX USA; 4 Dan L. Duncan Comprehensive Cancer Center, Baylor College of Medicine, Houston, TX USA; 5 Department of Molecular, Cellular and Developmental Biology, University of Colorado Boulder, Boulder, CO USA; 6 University of Colorado Cancer Center, Anschutz Medical Campus, Aurora, CO USA; 7 Charles C. Gates Center for Regenerative Medicine, University of Colorado Anschutz Medical Campus, Aurora, CO USA; 8 Molecular Medicine Program, Division of Urology, Department of Surgery, University of Utah School of Medicine, Salt Lake City, UT USA; 9 J. David Gladstone Institutes, San Francisco, CA USA; 10 Quantitative Biosciences Institute, University of California San Francisco, San Francisco, CA USA; 11 Department of Bioengineering and Therapeutic Sciences, University of California San Francisco, San Francisco, CA USA; 12 Department of Stem Cell Biology and Regenerative Medicine, Keck School of Medicine, University of Southern California, Los Angeles, CA USA; 13 BioFrontiers Institute, University of Colorado, Boulder, CO USA

**Keywords:** Embryonic stem cells, Totipotent stem cells, RNA metabolism

## Abstract

Controlling stem cell differentiation is a longstanding goal in biomedical research. Here we explore how cell fate is influenced by RNA condensates, specifically P-bodies, which modulate gene expression posttranscriptionally. We profiled the transcriptomes of biomolecular condensates in diverse developmental contexts spanning multiple vertebrate species. Our analyses revealed conserved, cell type-specific sequestration of untranslated RNAs encoding cell fate regulators. P-body RNA contents do not reflect active gene expression in each cell type but are enriched for translationally repressed transcripts characteristic of the preceding developmental stage. Mechanistically, P-body contents are controlled by microRNAs and can be profoundly reshaped by perturbing AGO2 or polyadenylation site usage. Applying these insights to stem cell differentiation, we show that manipulating P-body assembly or microRNA activity can direct naive mouse and human pluripotent stem cells toward totipotency or primed human embryonic cells toward the germ cell lineage. Our findings link cell fate decisions to RNA condensates across vertebrates and provide a means of controlling cell identity.

## Abstract (web summary)

Stem cell differentiation is controlled by manipulating RNA condensates.

## Main

Post-transcriptional mechanisms are critical for establishing and maintaining cell identity^1,2^. For instance, RNA splicing, decay and alternative polyadenylation are pivotal regulatory mechanisms during development, reprogramming and tissue homeostasis^3–10^. There is a growing appreciation that RNA regulation is associated with transcript compartmentalization in biomolecular condensates^11–16^. These condensates include P-bodies, evolutionarily conserved cytoplasmic structures containing RNA and RNA-binding proteins (RBPs)^11,17–22^. RNAs can be directed into P-bodies by proteins involved in translation repression, microRNA (miRNA)-mediated pathways and messenger RNA (mRNA) decay processes^17,20,23–26^. While P-bodies were initially characterized as hotspots for mRNA decay^20,21^, recent observations suggest that they also regulate translation by sequestering mRNA away from translational machinery^27–33^. In some contexts, P-body transcripts reenter the ribosome pool upon P-body dissolution^17,20,23–26,34^, suggesting that P-bodies provide a nuanced, dynamic mechanism to fine-tune gene expression.

Emerging evidence suggests a role for P-bodies in regulating cell fate by sequestering mRNA encoding chromatin remodelers and transcription factors^28,35,36^. P-body dysfunction is implicated in Parkinson’s disease^37^ and cancer^38–41^, suggesting that disrupted RNA sequestration may destabilize cell identity and contribute to pathologies. Still, a precise role for RNA sequestration in P-bodies during differentiation and development remains unclear. To address this knowledge gap, we purified intact P-bodies and quantified constituent coding and noncoding RNA across multiple cell types and species. Our findings revealed RNA sequestration in P-bodies as a conserved regulatory mechanism often leading to the storage of specific transcripts that reflect preceding developmental stages. We demonstrate that the ribosome occupancy and corresponding protein levels of mRNAs enriched in P-bodies, including fate-instructive transcripts, increased following acute dissolution of P-bodies. Mechanistically, miRNAs regulate mRNA sequestration in a context-dependent manner. Finally, we show that perturbing RNA sequestration activates a totipotency transcriptional program in naive human pluripotent cells and facilitates the conversion of primed human embryonic stem (ES) cells into primordial germ cell-like cells (PGCLCs). Our findings define a fundamental, conserved role for P-bodies in cell fate specification and suggest strategies for exploiting RNA condensates to direct cell identity.

## Results

### P-body purification

**Fig. 1:**
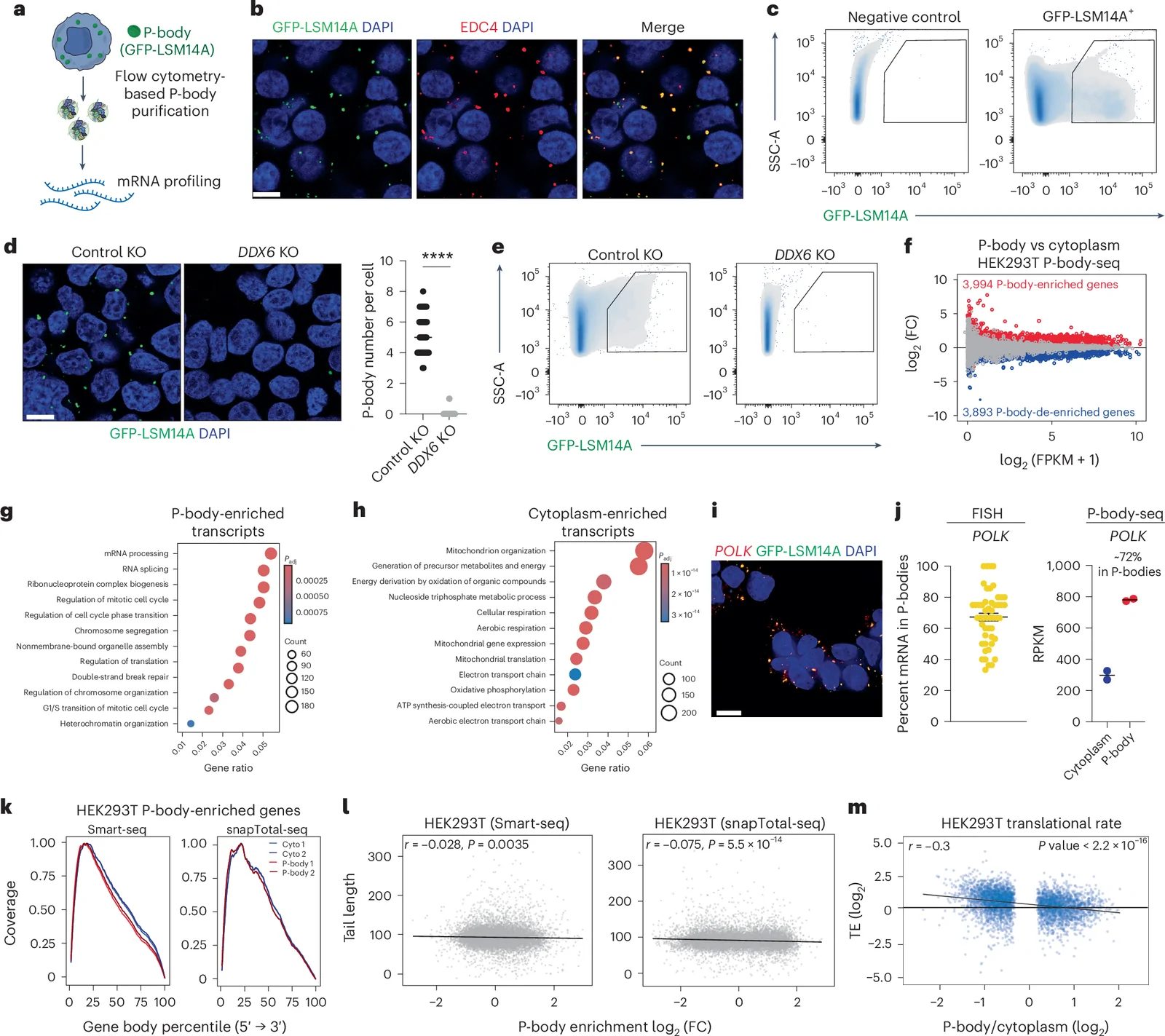
P-body-seq permits comprehensive profiling of P-body contents. **a**, Schematic for the purification and transcriptomic profiling of P-bodies from HEK293T cells based on GFP-LSM14A expression. **b**, Representative IF imaging of GFP-LSM14A puncta (green), colocalizing with EDC4 puncta (red) in HEK293T cells. Nuclei were counterstained with 4,6-diamidino-2-phenylindole (DAPI; blue) (scale bar, 10 μm). *n* = 3 independent experiments. **c**, Representative flow cytometry plots showing gating for GFP-LSM14A^+^ P-bodies in HEK293T cells. SSC, side scatter. **d**, Representative imaging of GFP-LSM14A puncta (green) in control and *DDX6*-KO HEK293T cells. Nuclei were counterstained with DAPI (blue) (scale bar, 10 μm) (left). P-body number in control (*n* = 50 cells) and *DDX6*-KO (*n* = 50 cells) HEK293T cells (right). Unpaired two-sided Student’s *t*-test, median ± s.d., ****P < 0.0001. **e**, Representative flow cytometry plots showing gating for GFP-LSM14A^+^ P-bodies in control and *DDX6*-KO HEK293T cells. **f**, MA plot of RNA-seq data depicting P-body-enriched genes in red and cytoplasm-enriched genes in blue in HEK293T cells (*n* = 2 biological independent samples per group, *P* < 0.05). FC, fold change; FPKM, fragments per kilobase of transcript per million mapped reads. **g**, GO pathway analysis of P-body-enriched mRNA in HEK293T cells, using expressed genes as background. Enrichment was tested using two-sided Fisher’s exact test with multiple-testing correction (Benjamini–Hochbergfalse discovery rate (FDR)). *P*_adj_, adjusted *P* value. **h**, GO pathway analysis of cytoplasm-enriched mRNA in HEK293T cells, using expressed genes as background. Enrichment was tested using two-sided Fisher’s exact test with multiple-testing correction (Benjamini–Hochberg FDR). **i**, Representative FISH imaging of *POLK* RNA molecules (red) combined with imaging of GFP-LSM14A puncta (green). Nuclei were counterstained with DAPI (blue) (scale bar, 10 μm). *n* = 3 independent experiments. **j**, Quantification of *POLK* mRNA molecules in P-bodies based on FISH (*n* = 50 cells; right) and P-body sequencing (right); median ± s.d. RPKM, reads per kilobase per million mapped reads. **k**, Read coverage distribution over the gene body of the longest annotated isoforms for genes enriched in P-bodies or the cytoplasm (cyto) in HEK293T cells. **l**, PolyA tail length as determined in ref. ^50^ compared to P-body enrichment based on Smart-seq and snapTotal-seq (Pearson correlation test). **m**, Translation efficiency (TE; log_2_ (Ribo-seq counts/RNA-seq counts)) negatively correlates with mRNA enrichment in P-bodies in HEK293T cells (Pearson correlation test (two sided), *P* = 3.94 × 10^−98^).

**Extended Data Fig. 1:**
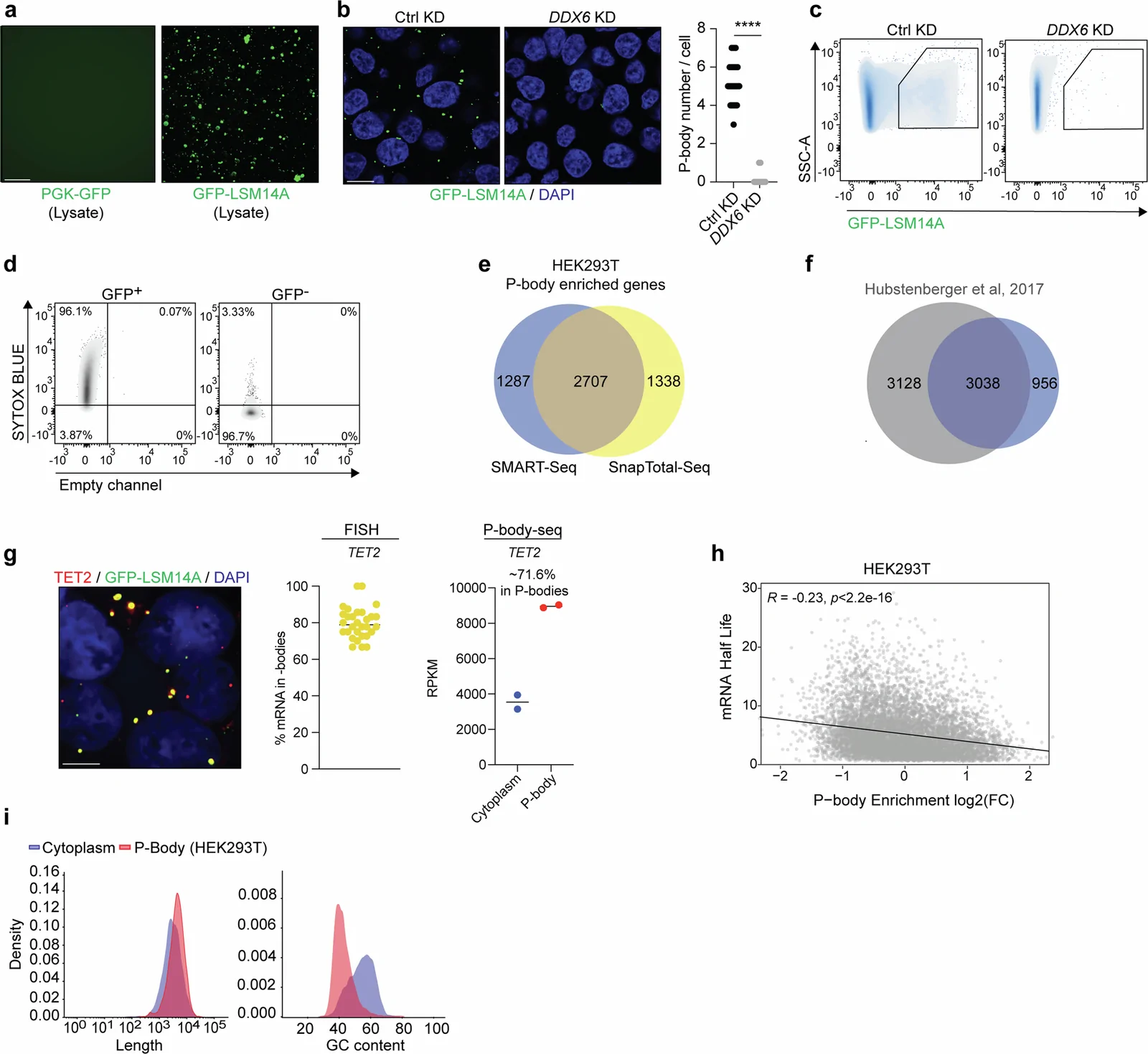
DDX6 suppression abolishes P-body formation. (**a**) Representative image of pre-sorted cell lysate containing GFP-LSM14A^+^ P-bodies (scale: 10μm). n=3 independent experiments. (**b**) Representative imaging of GFP-LSM14A puncta (green) in control and *DDX6* KD HEK293T cells. Nuclei were counterstained with DAPI (blue) (scale: 10μm) (left panel). P-body number in control (n=50 cells) and *DDX6* KD (n=50 cells) HEK293T cells (right panel). Unpaired two-sided Student’s t-test, median ± s.d., ****: p<0.0001. (**c**) Representative flow cytometry plots showing gating for GFP-LSM14A^+^ P-bodies in control and DDX6 KD HEK293T cells. (**d**) Representative flow cytometry plots showing gating for SYTOX BLUE^+^ events in GFP^+^ and GFP^−^ gates of pre-sorted cytoplasmic fraction from HEK293T cells. (**e**) Venn diagram showing the overlap between P-body-associated mRNAs in HEK293T from SMART-Seq and SnapTotal-Seq. (**f**) Venn diagram showing the overlap between P-body-associated mRNAs in HEK293T of this study (blue) and a published dataset from^29^ (grey). (**g**) Representative FISH imaging of *TET2* RNA molecules (red) combined with imaging of GFP-LSM14A puncta (green). Nuclei were counterstained with DAPI (blue) (scale: 5 μm). Quantification of TET2 mRNA molecules in P-bodies based on FISH (n=30 cells; left) and P-body sequencing (right). (**h**) Correlation between mRNA half-life as determined in ref. ^49^ and P-body enrichment in HEK293T cells, Pearson correlation test (two-sided), p=3.08e-106. (**i**) Length (left panel) and GC content (right panel) density plots of mRNAs in purified P-body and cytoplasmic fractions of HEK293T cells.

To profile P-body contents, we adapted a fluorescence-activated sorting method^29,42^ using a green fluorescent protein (GFP)-LSM14A expression construct in HEK293T cells (Fig. 1a). LSM14A is an established protein component of P-bodies^29^, and GFP-LSM14A puncta colocalized with the P-body marker EDC4 (ref. ^43^) (Fig. 1b). After cell lysis, intact GFP-LSM14A particles were detectable by microscopy and were readily isolated using fluorescence-activated particle sorting (FAPS) (Fig. 1c and Extended Data Fig. 1a). By contrast, particles were undetectable in cells expressing a cytoplasmic GFP control (Fig. 1c and Extended Data Fig. 1a). To corroborate these data, we depleted *DDX6* in GFP-LSM14A HEK293T cells to disrupt P-bodies^19,21,28,32^. Both short hairpin RNA (shRNA)-mediated suppression of *DDX6* and CRISPR knockout (KO) resulted in the complete loss of GFP^+^ particles (Fig. 1d,e and Extended Data Fig. 1b,c). To confirm the presence of RNA in GFP-LSM14A particles, we stained lysates from GFP-LSM14A-expressing cells with SYTOX Blue, an RNA-binding fluorescent dye. We observed RNA in GFP^+^ particles but not in GFP^−^ particles (Extended Data Fig. 1d). These findings confirm the robust labeling and isolation of P-bodies using GFP-LSM14A.

We characterized RNA from purified P-bodies and corresponding cytosolic fractions using Smart-seq^44^ (hereafter P-body-seq). We detected 3,994 mRNAs enriched in P-bodies of HEK293T cells relative to the cytosol (Fig. 1f). Gene ontology (GO) analysis revealed that P-body-enriched mRNAs encoded regulators of RNA processing, transcription, chromatin organization and cell cycle (Fig. 1g), while cytosolic mRNAs were involved in housekeeping functions such as metabolic processes and structural components (Fig. 1h). To ensure that our analyses were not biased by polyA-directed library preparations, we performed snapTotal-seq^45^, an alternative low-input library preparation that uses random primers rather than oligo(dT). snapTotal-seq results were highly consistent with Smart-seq data (~70% overlap; Extended Data Fig. 1e). Moreover, we compared our dataset to P-body-enriched transcripts previously reported in HEK293T cells^29^ and found that 3,038 mRNAs (76%) were shared between the datasets (Extended Data Fig. 1f). To confirm P-body-seq results using an orthogonal approach, we employed single-molecule fluorescence in situ hybridization (FISH) (smFISH) in conjunction with immunofluorescence (IF)^46^ and verified the localization of *POLK* and *TET2* within P-bodies (Fig. 1i,j and Extended Data Fig. 1g). In line with previous reports^26,38^, approximately 70% of total *POLK* and *TET2* mRNA was localized to P-bodies determined using either smFISH or P-body-seq, demonstrating substantial transcript sequestration in P-bodies.

P-bodies have been proposed as sites for RNA decay^20,21,47,48^; however, P-body enrichment showed poor correlation with transcript half-life measurements in HEK293T cells^49^, consistent with previous reports^29^ (Extended Data Fig. 1h). Read distribution analysis also showed no evidence of increased truncated transcripts in P-bodies relative to the cytoplasm (Fig. 1k), in line with previous reports^26,29,38^. In addition, we observed poor correlation between mean polyA tail length^50^ and P-body enrichment (*r* = −0.028 in Smart-seq data and *r* = −0.075 in snapTotal-seq data) (Fig. 1l), indicating that P-body-localized RNAs are intact and are not preferentially deadenylated relative to cytoplasmic transcripts.

We next asked whether properties intrinsic to RNAs correlated with P-body enrichment. Transcript length had no appreciable relationship with P-body localization, but transcripts with high AU content were enriched among P-body-targeted mRNAs (Extended Data Fig. 1i). Notably, AU-rich sequences are associated with inefficient translation^28,51,52^. Accordingly, analysis of published ribosome profiling data obtained in HEK293T cells^50^ revealed reduced translation efficiency for P-body-associated transcripts (Fig. 1m). These data suggest that transcripts sequestered in P-bodies are translationally repressed compared to cytoplasmic-enriched mRNAs.

### P-body contents are cell type specific

**Fig. 2:**
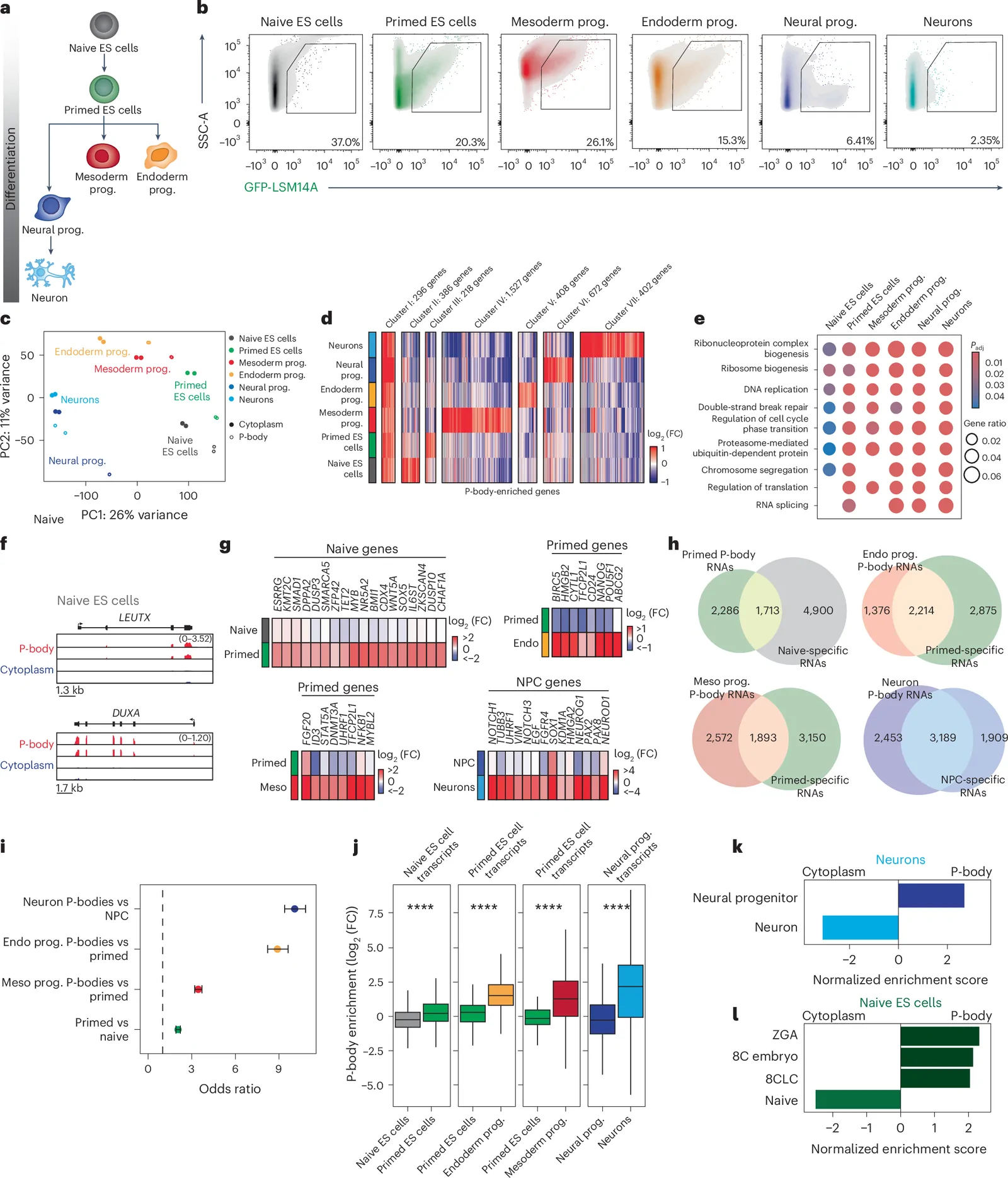
RNA sequestration in P-bodies is cell type specific. **a**, Schematic highlighting developmental stages profiled by P-body-seq. Prog., progenitor. **b**, Representative flow cytometry plots showing gating for GFP-LSM14A^+^ P-bodies in the indicated samples. **c**, Principal-component (PC) analysis of RNA-seq data for the indicated samples. **d**, Heatmap showing expression levels of differentially enriched mRNAs between purified P-body fractions of the indicated samples, with manual clustering based on P-body-enriched genes (log_2_ (FC) > 0, *P* value < 0.05) that are specific to each cell type or enriched in all cell types (cluster I). Gene number in each cluster is indicated in the figure. Differential enrichment was assessed using DESeq2 (two-sided Wald test, Benjamini–Hochberg *P*_adj_ values, *P* < 0.05; *n* = 2 biologically independent samples per group). **e**, GO pathway analysis using expressed genes as a background for P-body-enriched mRNA in the indicated samples. Enrichment was tested using two-sided Fisher’s exact test with multiple-testing correction (Benjamini–Hochberg FDR). **f**, Gene tracks showing 8C-related genes from RNA-seq data of P-bodies and cytoplasm fractions of naive ES cells. **g**, log_2_ (FC) of current cell fate markers in the current and downstream cell fate, where log_2_ (FC) > 0 indicates P-body enrichment and log_2_ (FC) < 0 indicates P-body depletion. Endo, endoderm; meso, mesoderm; NPC, neural progenitor cell. **h**, Venn diagrams showing the overlap of P-body-enriched genes in the downstream cell type (log_2_ (FC) > 0, *P* < 0.05) with genes that are more highly expressed in the current cell type than in the downstream cell type (current/downstream log_2_ (FC) > 0, *P* < 0.05). Differential enrichment was assessed using DESeq2 (two-sided Wald test, Benjamini–Hochberg *P*_adj_ values, *P* < 0.05). *n* = 2 biologically independent samples per group. **i**, Odds ratio of the overlap between the comparisons in **h**. Center points represent the odds ratio, and error bars represent 95% confidence intervals; *n* of gene sets is reflected in **h**. Dashed line indicates an odds ratio of 1 (odds ratios >1 indicate positive association between groups). **j**, log_2_ (FC) of current fate lineage-specific genes (current/downstream log_2_ (FC) > 2.5, *P* < 0.05) in the current and downstream cell state; box plots show the median (center line), interquartile range (IQR; box, 25th–75th percentiles) and whiskers extending to 1.5× the IQR). Differential enrichment was assessed using DESeq2 (two-sided Wald test, Benjamini–Hochberg *P*_adj_ values, *P* = 3.9 × 10^−23^, *P* = 6.4 × 10^−54^, *P* = 2.3 × 10^−29^, *P* = 1.1 × 10^−37^). *n* = 2 biologically independent samples per group (*n* of gene sets is reflected in **h**). **k**, GSEA performed on P-body-versus-cytoplasm differential expression in neurons for the neural progenitor-related gene expression signature and the neuron-related gene expression signature. Enrichment significance was calculated with the permutation test (two sided), with multiple-testing correction using the Benjamini–Hochberg method (FDR < 0.05). **l**, GSEA performed on P-body-versus-cytoplasm differential expression in human naive ES cells for human blastomere and naive ES cell-related gene sets from refs. ^58,103^. Enrichment significance was calculated with the permutation test (two sided), with multiple-testing correction using the Benjamini–Hochberg method (FDR < 0.05).

**Extended Data Fig. 2:**
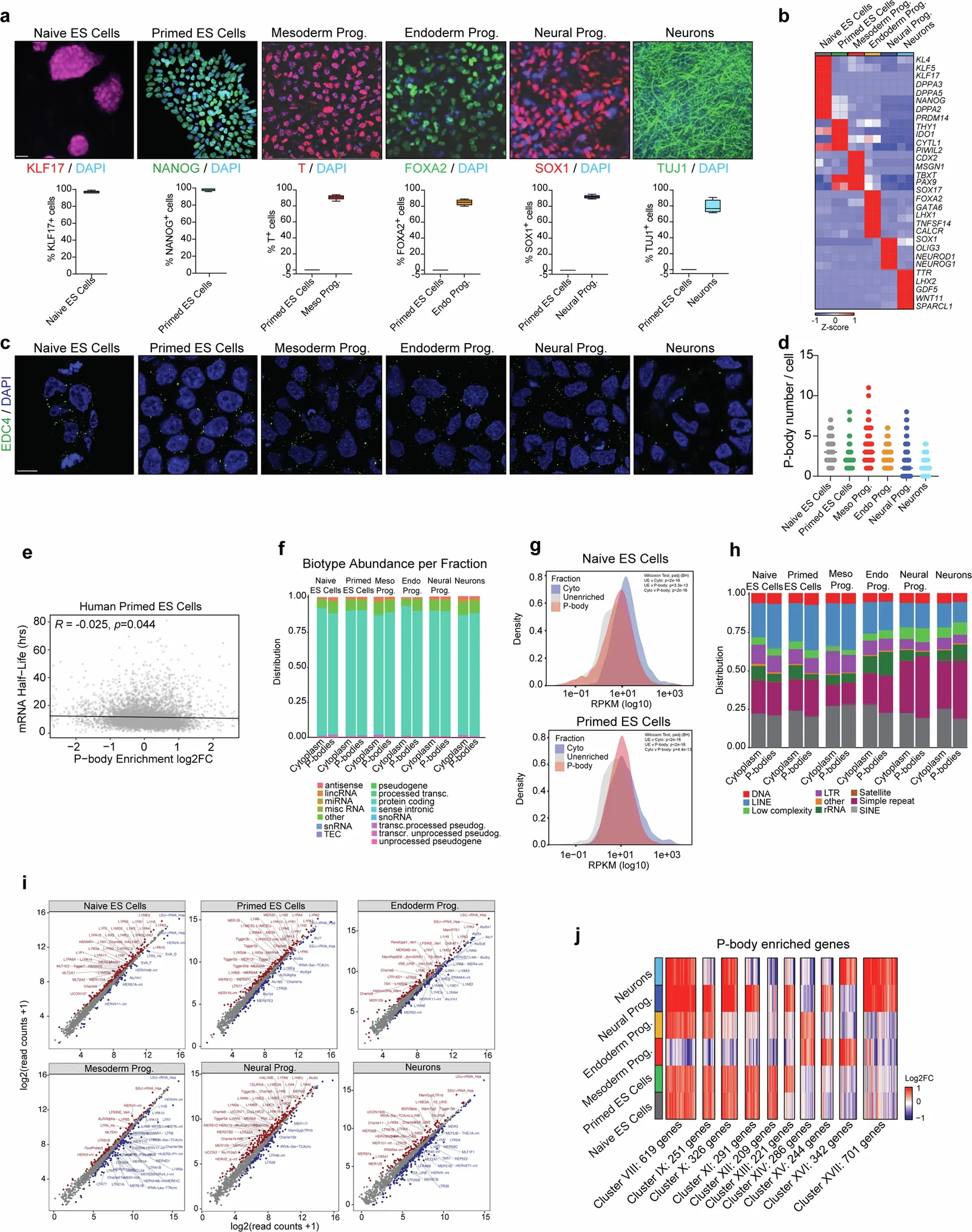
Directed differentiation of human ES cells permits analysis of P-body contents. (**a**) Representative IF images of lineage-specific markers for the indicated samples. Nuclei were counterstained with DAPI (blue) (scale: 25μm) (upper panel). Quantification of lineage-specific positive cells in the indicated samples (bottom panel). Box plots show the distribution of the data with the box spanning the 25th to 75th percentiles (interquartile range, IQR), the horizontal line inside the box indicating the median, and whiskers extending to the minimum and maximum values. (**b**) Heatmap showing expression levels of lineage-specific genes for the indicated samples based on RNA-seq analysis (n=2 biologically independent samples per group). (**c**) Representative IF imaging of EDC4 puncta (green) in the indicated samples. Nuclei were counterstained with DAPI (blue) (scale: 10μm). (**d**) P-body number in the indicated samples. Naïve human ES cells (n=70 cells), primed human ES cells (n=90 cells), mesoderm progenitors (n=90 cells), endoderm progenitors (n=90 cells), neural progenitors (n=90 cells), neurons (n=90 cells), median ± s.d. (**e**) Correlation between mRNA half-life as determined in Ref. ^57^ and P-body enrichment in primed human ES cells, Pearson correlation test (two-sided). (**f**) Distribution of biotypes within the P-bodies and cytoplasm of each cell type. (**g**) Density plots of mean RPKM for genes in each category: P-body enriched (log2FC>0, p<0.05), cytoplasm enriched (log2FC<0, p<0.05), or unenriched (everything else). Two-sided Wilcoxon test with Benjamini–Hochberg adjustment shown. Cliff’s delta effect size statistic in primed (Cyto v Unenriched=0.32, Cyto v P-body=0.11, P-body v Unenriched=0.24) and in naïve (Cyto v Unenriched=0.4, Cyto v P-body=0.33, P-body v Unenriched=0.10), where r <0.1 is negligible, 0.1-0.3 is small, 0.3-0.5 is medium, and > 0.5 is a large effect. (**h**) Ratio of repetitive elements in purified P-body vs. cytoplasmic fractions of the indicated samples. (**i**) Differential expression analysis of transposable element expression in purified P-body vs. cytoplasmic fractions of the indicated samples. *red: padj<0.05, log2FC>0, blue: padj<0.05, log2FC<0. Differential enrichment was assessed using DESeq2 (two-sided Wald test, Benjamini–Hochberg adjusted p-values). n=2 biologically independent samples per group. (**j**) Heatmap showing expression levels of differentially enriched mRNAs between purified P-body fractions of the indicated samples. Manual clustering represents P-body enriched genes that are shared between two or more cell types. Gene number in each cluster is indicated in the figure (n=2 biologically independent samples per group, p<0.05).

P-body-associated transcripts can reenter translation following genetic or environmental perturbation^19,25,29,51,53,54^, suggesting that their sequestration may influence developmental fate decisions. Yet, defining P-body function in complex biological processes has been challenging, in part because sequencing-based approaches to define P-body contents have been limited to nonvertebrates or transformed cell lines^25,29,55^. We thus applied P-body-seq to profile transcripts in various human cell types across developmental stages. We stably integrated GFP-LSM14A at the *AAVS1* locus in human ES cells and cultured them under naive and primed conditions, which model the pre- and post-implantation epiblast, respectively^56^. We also induced differentiation of the same human pluripotent stem cell line into progenitors of all three germ layers (mesoderm, endoderm and neural progenitors) and ultimately into neurons (Fig. 2a). IF confirmed robust differentiation into the expected lineages (80% efficiency or greater; Extended Data Fig. 2a), which was supported by the expression of lineage-specific genes (Extended Data Fig. 2b). We detected EDC4^+^ P-bodies in each cell type, albeit in varying amounts (Extended Data Fig. 2c,d), suggesting differences in RNA sequestration between cell types.

To profile P-body contents, we sorted GFP-LSM14A^+^ particles from each cell type. Particle size and fluorescence varied between cell types (Fig. 2b), likely reflecting differential expression of GFP-LSM14A from the *AAVS1* locus, but, in all cases, we isolated sufficient RNA for P-body-seq. Supporting our observations in HEK293T cells, analysis of half-life data in human pluripotent stem cells^57^ suggested that RNA in P-bodies is not preferentially degraded compared to cytoplasmic RNA (Extended Data Fig. 2e). We observed diverse RNA biotypes in P-bodies, including protein-coding mRNA and lincRNA (Extended Data Fig. 2f). We note, however, that RNA sequestration in P-bodies is not merely a reflection of expression level, as the expression of P-body-enriched transcripts was distributed similarly relative to that of cytoplasmic and unenriched transcripts (Extended Data Fig. 2g). We likewise identified repetitive elements in P-bodies, although we did not detect marked differences in their abundance between P-bodies and the cytoplasm (Extended Data Fig. 2h,i). Based on principal-component analysis, we observed unique mRNA profiles for P-bodies from each cell type, suggesting that distinct cell identities have unique transcripts stored in P-bodies (Fig. 2c). Principal-component analysis further suggested a stepwise progression from naive ES cells toward mesoderm and/or endoderm lineages on one path and the ectoderm lineage on another (Fig. 2c). Notably, we found that P-body contents did not necessarily cluster most closely with the cytoplasm from the same cell type (Fig. 2c). For example, P-body samples from neurons cluster most closely with cytoplasmic samples from neural progenitors and P-body contents of mesoderm progenitors cluster with the cytoplasm of primed ES cells (Fig. 2c). These data suggest that P-bodies sequester transcripts from stem and progenitor cells to suppress their protein-level expression during differentiation.

### P-body contents reflect preceding human developmental stages

**Extended Data Fig. 3:**
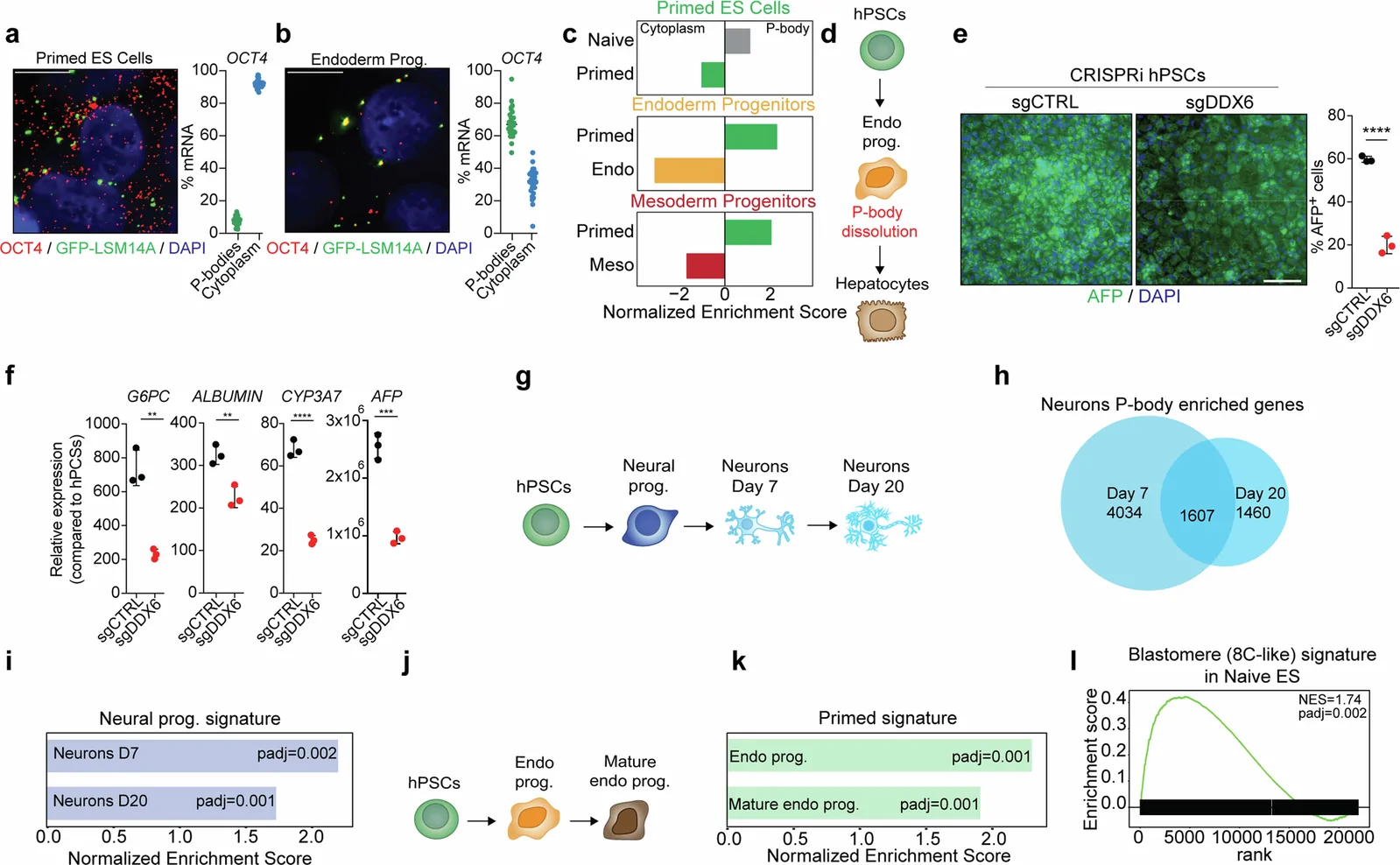
Transient sequestration of developmental stage-specific RNAs in P-bodies confers plasticity during early development. (**a, b**) Representative FISH imaging of *OCT4* RNA molecules (red) combined with imaging of GFP-LSM14A puncta (green) in primed ES cells (**a**) and endoderm progenitors (**b**) with corresponding quantification (n=30 cells; right). Nuclei were counterstained with DAPI (blue) (scale: 5μm). (**c**) GSEA of P-body vs cytoplasm differential expression in human primed ES cells, endoderm progenitors, and mesoderm progenitors, with gene sets related to the differentiated and preceding developmental stage. (**d**) A schematic of the strategy for P-body dissolution in human endoderm progenitors during their differentiation to hepatocytes. (**e**) Representative IF imaging of AFP (green) positive cells in hPSCs upon *DDX6* KO compared to control cells. Nuclei were counterstained with DAPI (blue) (scale: 100μm) (Left panel). Quantification of AFP^+^ cells upon *DDX6* suppression. Unpaired two-sided Student’s t-test, sgControl (n=3 fields), sgDDX6 (n=3 fields), mean ± s.d., ****: p<0.0001 (Right panel). (**f**) qRT-PCR analysis for the indicated genes in hepatocytes. Unpaired two-sided Student’s t-test, mean ± s.d., n=3 biologically independent samples per group,**: p<0.01, ****: p<0.0001; p=0.0013, p=0.0071, p=0.0004. (**g**) A schematic of neuron maturation. (**h**) Venn diagram showing the overlap between P-body-associated mRNAs in neurons cultured for 7 and 20 days. (**i**) GSEA of P-body vs cytoplasm differential expression in day 7 and day 20 neurons, with a neural progenitor related gene sets. Enrichment significance was calculated by permutation test (two-sided), with multiple testing correction using the Benjamini–Hochberg method. (**j**) A schematic of endoderm progenitor maturation. (**k**) GSEA of P-body vs cytoplasm differential expression in endoderm progenitors and mature endoderm progenitors with a gene set for primed human ES cell-related genes. Enrichment significance was calculated by permutation test (two-sided), with multiple testing correction using the Benjamini–Hochberg method. (**l**) GSEA of P-body vs cytoplasm differential expression in naïve human ES cells with a gene set for 8-cell embryo^58^. Enrichment significance was calculated by permutation test (two-sided), with multiple testing correction using the Benjamini–Hochberg method.

We grouped transcripts based on their enrichment across cell types (Fig. 2d and Extended Data Fig. 2j). A fraction of transcripts was enriched in the P-bodies of all cell types, and GO categories linked to DNA damage and cell cycle progression were common across cell types (Fig. 2e). By contrast, transcripts enriched in the P-bodies of single cell types encoded fate-instructive factors, such as *LEUTX* and *DUXA* in naive ES cells (Fig. 2f). Both *LEUTX* and *DUXA* are associated with totipotency and the eight-cell (8C) embryo^58,59^, the developmental stage preceding naive pluripotency. To explore whether P-bodies sequester transcripts important for stem and progenitor cell maintenance during differentiation, we identified P-body-enriched transcripts related to cell fate and examined their localization in differentiated cell types and corresponding developmental precursors (Fig. 2g). P-bodies of differentiated cell types contained transcripts associated with the preceding developmental state, while these transcripts were largely cytoplasmic or unenriched in precursor cells (Fig. 2g and Discussion). smFISH analysis of *OCT4* (*POU5F1*) in human primed ES cells and endoderm progenitors confirmed these findings, with *OCT4* transcripts transitioning from primarily cytoplasmic localization in primed ES cells to P-bodies during endoderm differentiation (Extended Data Fig. 3a,b). We also observed a significant overlap between transcripts that were more expressed in precursor cells and P-body enriched in differentiated cells (Fig. 2h–j). Finally, we conducted gene set enrichment analysis (GSEA) using differential gene expression data from P-body and cytoplasmic fractions for each cell type (Fig. 2k,l and Extended Data Fig. 3c). In neurons, we observed robust enrichment of a transcriptional signature characteristic of neural progenitor cells in the P-body fraction (Fig. 2k). Consistent with these findings, P-body dissolution impaired neuronal specification from neural progenitors^28,35,36^, providing functional evidence that P-bodies sequester genes related to stem and progenitor cell self-renewal to facilitate differentiation. Conversely, a neuronal transcriptional signature was enriched in the cytoplasm of neurons, where these transcripts are expected to be available to translational machinery (Fig. 2k).

Next, we assessed the functional requirement of P-bodies in endoderm differentiation using a CRISPR interference (CRISPRi)-based approach to deplete DEAD-box helicase 6 (DDX6)^28^ and dissolve P-bodies^19^ (Extended Data Fig. 3d). DDX6 depletion significantly impaired differentiation toward AFP^+^ hepatocytes (Extended Data Fig. 3e) and suppressed the induction of other hepatic differentiation markers such as *ALB*, *G6PC* and *CYP3A7* compared to control cells (Extended Data Fig. 3f). These results suggest that P-bodies are essential for differentiation to hepatocytes.

To test whether P-body-associated transcripts eventually decline after differentiation is completed, we extended neuronal differentiation to 20 d (Extended Data Fig. 3g). Analysis of P-body contents in mature neurons showed substantial overlap (~50%) with transcripts sequestered from neurons at day 7 of differentiation (Extended Data Fig. 3h); however, prolonged culture led to progressive depletion of neural progenitor-associated transcripts from P-bodies (Extended Data Fig. 3i). Similar patterns were observed in mature endoderm progenitors, which showed decreased enrichment for primed ES cell expression profiles compared to newly differentiated endoderm progenitors (Extended Data Fig. 3j,k).

We next examined P-body-associated transcripts across pluripotent states. In primed ES cells, P-bodies showed enrichment of naive ES cell-specific gene signatures (Extended Data Fig. 3c), consistent with observations that P-body dissolution promotes primed-to-naive state conversion^28^. Similarly, P-bodies in naive ES cells were enriched for gene expression signatures characteristic of the preceding developmental stage, including zygotic genome activation (ZGA), the 8C embryo and 8C-like cells (8CLC)^58,59^ (Fig. 2l and Extended Data Fig. 3l). While naive human ES cells typically express low levels of ZGA- and 8CLC-related transcripts^58^, our data suggest that these transcripts are sequestered into P-bodies to prevent their translation and inhibit inappropriate reversion to an earlier developmental state. Conversely, naive pluripotency transcripts were enriched in the cytoplasm relative to P-bodies (Fig. 2l), likely reflecting active expression of genes supporting the naive cell fate. These findings suggest that RNA condensates in ES cells selectively sequester totipotency-associated factors. Altogether, these data indicate that P-body contents do not simply reflect the gene expression profiles of a given cell type but are instead enriched for transcripts characteristic of the preceding developmental stage.

### RNA sequestration is conserved across vertebrates

**Fig. 3:**
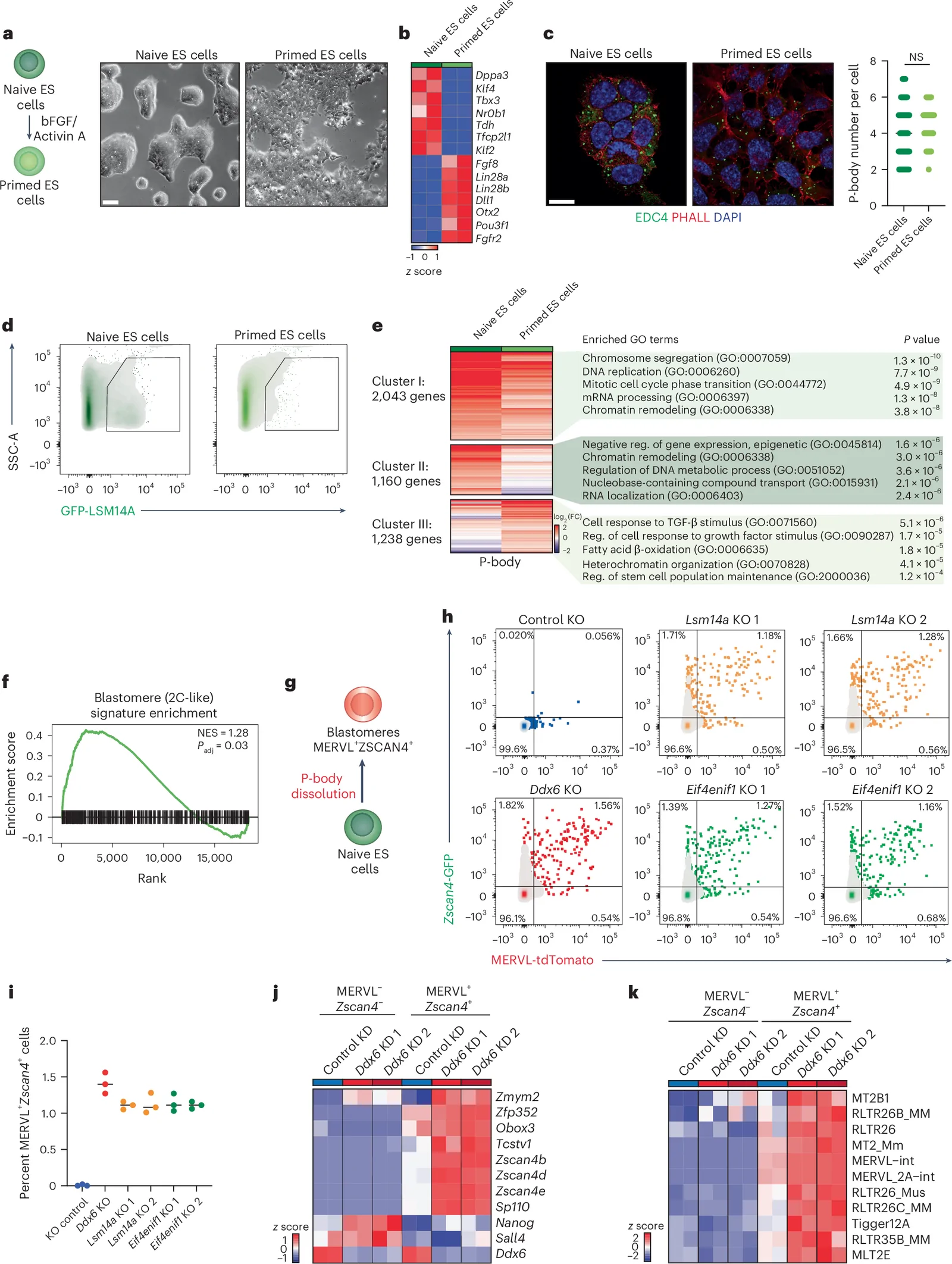
RNA sequestration in P-bodies safeguards mouse ES cell identity. **a**, Schematic of the conversion from naive to primed state mouse ES cells (left). Representative bright-field images of naive and primed mouse ES cells (scale bar, 50 μm) (right). *n* = 3 independent experiments. bFGF, basic fibroblast growth factor. **b**, Heatmap showing expression levels of naive-specific and primed-specific transcripts (*n* = 2 biological independent samples per group). **c**, Representative IF imaging of EDC4 puncta (green) in naive and primed mouse ES cells. Cell membranes were labeled with phalloidin (PHALL; red), and nuclei were counterstained with DAPI (blue) (scale bar, 10 μm) (left). P-body numbers in naive (*n* = 60 cells) and primed (*n* = 60 cells) mouse ES cells (right). Unpaired two-sided Student’s *t*-test, median ± s.d.; not significant (NS), *P* > 0.05, *P* = 0.0843. **d**, Representative flow cytometry plots showing gating for GFP-LSM14A^+^ P-bodies in naive and primed mouse ES cells. **e**, Heatmap showing expression levels of differentially enriched mRNAs between purified P-body fractions of naive and primed mouse ES cells, with GO pathway analysis using expressed genes as a background for P-body-enriched transcripts for the indicated clusters. Clusters were generated manually to represent genes that are P-body enriched in both naive and primed mouse ES cells or specifically enriched in one cell type. Gene number in each transcript cluster is indicated in the figure (*n* = 2 biological independent samples per group, *P* < 0.05; reg., regulation). **f**, GSEA analysis of blastomere-related genes^63^ in the purified P-body fraction versus the cytoplasmic fraction from naive mouse ES cells (normalized enrichment score (NES) = 1.28, *P* = 0.03). Enrichment significance was calculated using a permutation test (two sided), with multiple-testing correction using the Benjamini–Hochberg method. **g**, Schematic of the strategy for P-body dissolution in mouse naive ES cells carrying blastomere-specific reporters (MERVL-tdTomato and *Zscan4*-GFP). **h**,**i**, Flow cytometric analysis (**h**) and quantification (**i**) of MERVL-tdTomato^+^ and *Zscan4*-GFP^+^ cells upon *Lsm14a*, *Ddx6* and *Eif4enif1* KO in mouse naive ES cells. Control, *Lsm14a*, *Ddx6* KO and *Eif4enif1* KO (*n* = 3 biological independent samples per group, median ± s.d.). *Tcstv1* (*Tcstv1a*). **j**,**k**, RNA-seq data from sorted MERVL- and *Zscan4*-negative and -positive mouse ES cells after *Ddx6* KD, showing expression levels of pluripotency- and totipotency-related transcripts (**j**) and transposable elements (**k**).

**Extended Data Fig. 4:**
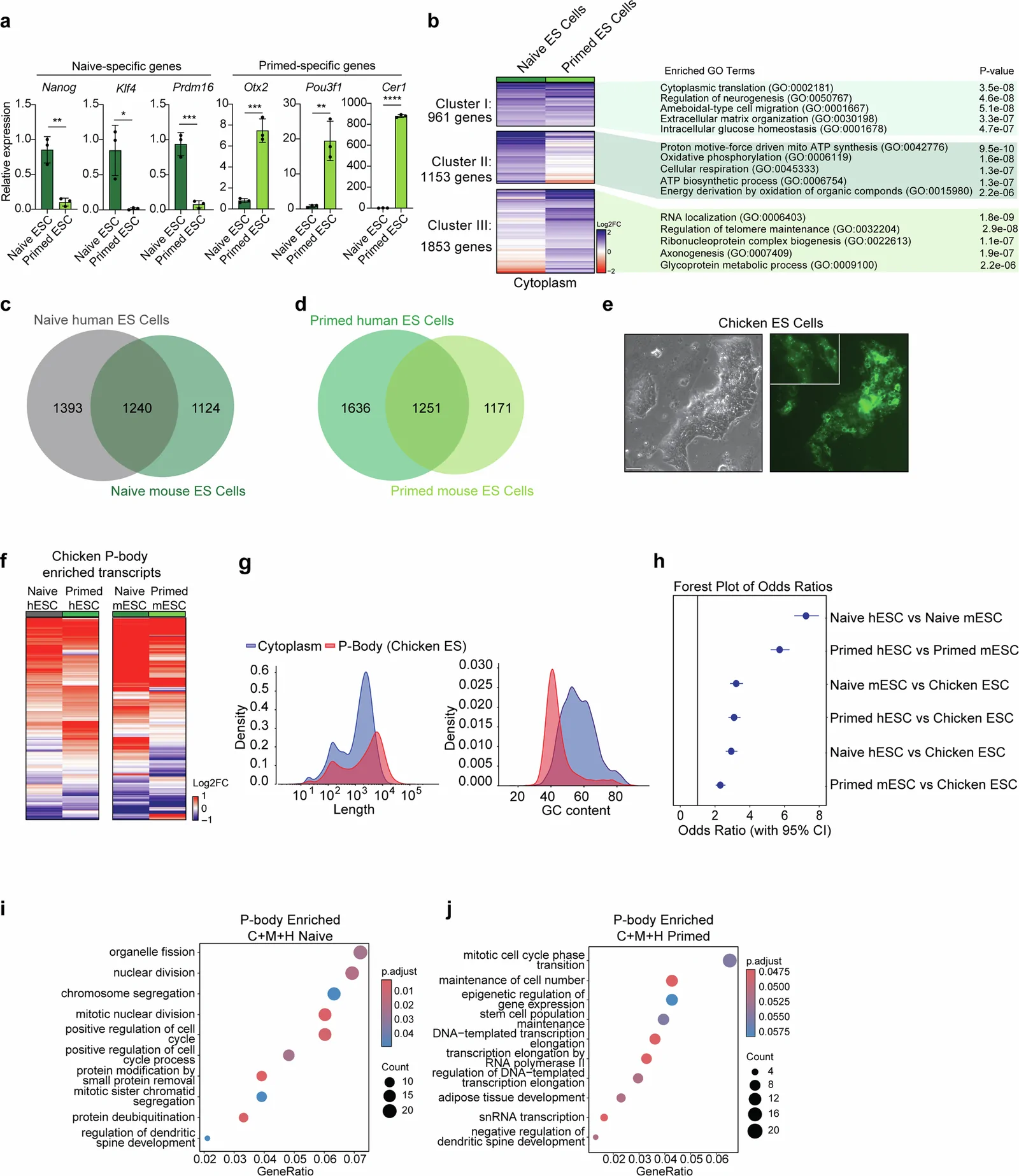
RNA sequestration in P-bodies is conserved across vertebrates. (**a**) qRT-PCR analysis of naïve-specific and primed-specific gene expression in naïve and primed mouse ES cells (n=3 biologically independent samples per group). Unpaired two-sided Student’s t test, mean ± s.d., *: p<0.05, **: p<0.01, ***: p<0.001, ****: p<0.0001; p=0.0028, p=0.059, p=0.0010, p=0.0005, p=0.0045. (**b**) Heatmap showing expression levels of differentially enriched mRNAs between cytoplasmic fractions of naïve and primed mouse ES cells, with GO pathway analysis of upregulated genes in each cluster. Clusters were generated manually to reflect genes that are cytoplasm enriched in both or one of the cell types. Gene number in each cluster is indicated in the figure (n=2 biologically independent samples per group, p<0.05). (**c, d**) Venn diagrams showing the overlap between P-body enriched mRNAs in human and mouse naïve ES cells (**c**) and human and mouse primed ES cells (**d**). (**e**) Representative bright field image (left panel) and GFP expression (right panel) in GFP-LSM14A chicken ES cells (scale: 50μm). n=3 independent experiments. (**f**) Heatmap showing P-body enrichment in human and mouse primed and naïve cells for genes enriched in P-bodies from chicken ES cells. (**g**) Length (left panel) and GC content (right panel) density plots of mRNAs enriched in purified P-body and cytoplasmic fractions of chicken ES cells. (**h**) Forest Plot of odds ratios for P-body enriched genes in each cell type; Center points represent the odds ratio, and error bars represent 95% confidence intervals (CI), n naive hESC=2633, n primed hESC=2980, n naive mESC=2379, n primed mESC=2439, n chicken ESC=1515. (**i, j**) GO pathway analysis of P-body enriched mRNAs, showing common pathways between chicken ES cells and mouse and human naïve (**i**) and primed (**j**) ES cells. Enrichment was tested by two-sided Fisher’s exact test with multiple testing correction (Benjamini–Hochberg method).

Next, we investigated whether P-body contents and functions are conserved across vertebrate species. We first considered mouse naive and primed ES cells^60^. Similar to their human counterparts, naive mouse ES cells resemble the in vivo pre-implantation mouse epiblast, while the primed state represents post-implantation epiblast cells^61^. Conversion from naive to primed mouse ES cells was efficient (Fig. 3a,b and Extended Data Fig. 4a), with both cell types harboring similar numbers of P-bodies (Fig. 3c).

P-body-seq analyses of mouse naive and primed ES cells (Fig. 3d) revealed that a significant fraction of P-body-enriched transcripts exhibited cell type-specific localization (54%) (Fig. 3e and Extended Data Fig. 4b), reinforcing our observations in human pluripotent stem cells. GO analysis revealed that transcripts enriched in P-bodies of both naive and primed ES cells were associated with cell cycle progression and RNA processing, similar to human datasets (Fig. 3e). Transcripts exclusively enriched in P-bodies of naive and primed ES cells were associated with chromatin remodeling and growth factor response, respectively (Fig. 3e).

Direct cross-species comparison revealed that ~50% of P-body-enriched transcripts were shared between mice and humans in both naive and primed cell states (Extended Data Fig. 4c,d).

To test whether this mechanism is conserved in phylogenetically distant vertebrates, we transduced chicken ES cells with a GFP-LSM14A lentivirus. Chicken ES cells are derived at the blastoderm stage and contribute to chimeric embryos^62^. In line with our findings in human and mouse cells, we detected GFP-LSM14A particles in chicken ES cells (Extended Data Fig. 4e), which we purified by FAPS. After P-body-seq, we found that mRNAs enriched in P-bodies of chicken ES cells exhibited extensive overlap with the P-body transcriptomes of mice and humans (Extended Data Fig. 4f). Comparative analysis revealed similar transcript features among all three species, particularly in the preferential sequestration of AU-rich mRNA in P-bodies, regardless of transcript length (Extended Data Fig. 4g). Thus, RNA sequestration in P-bodies is conserved across vertebrates.

Further analysis using the odds ratio statistic identified a significant positive association of chicken ES cells with both mouse and human ES cells, with the strongest association observed between mouse and human samples (Extended Data Fig. 4h). GO analysis of P-body-associated transcripts shared between chicken ES cells and mouse and human naive and primed ES cells revealed enrichment in categories related to RNA processing, transcription, chromatin organization and cell cycle (Extended Data Fig. 4i,j). These data indicate that transcripts sequestered in chicken ES cell P-bodies encode important regulatory factors, consistent with our findings in human and mouse ES cells.

### Disrupting P-bodies induces translation of totipotency proteins in mouse ES cells

**Extended Data Fig. 5:**
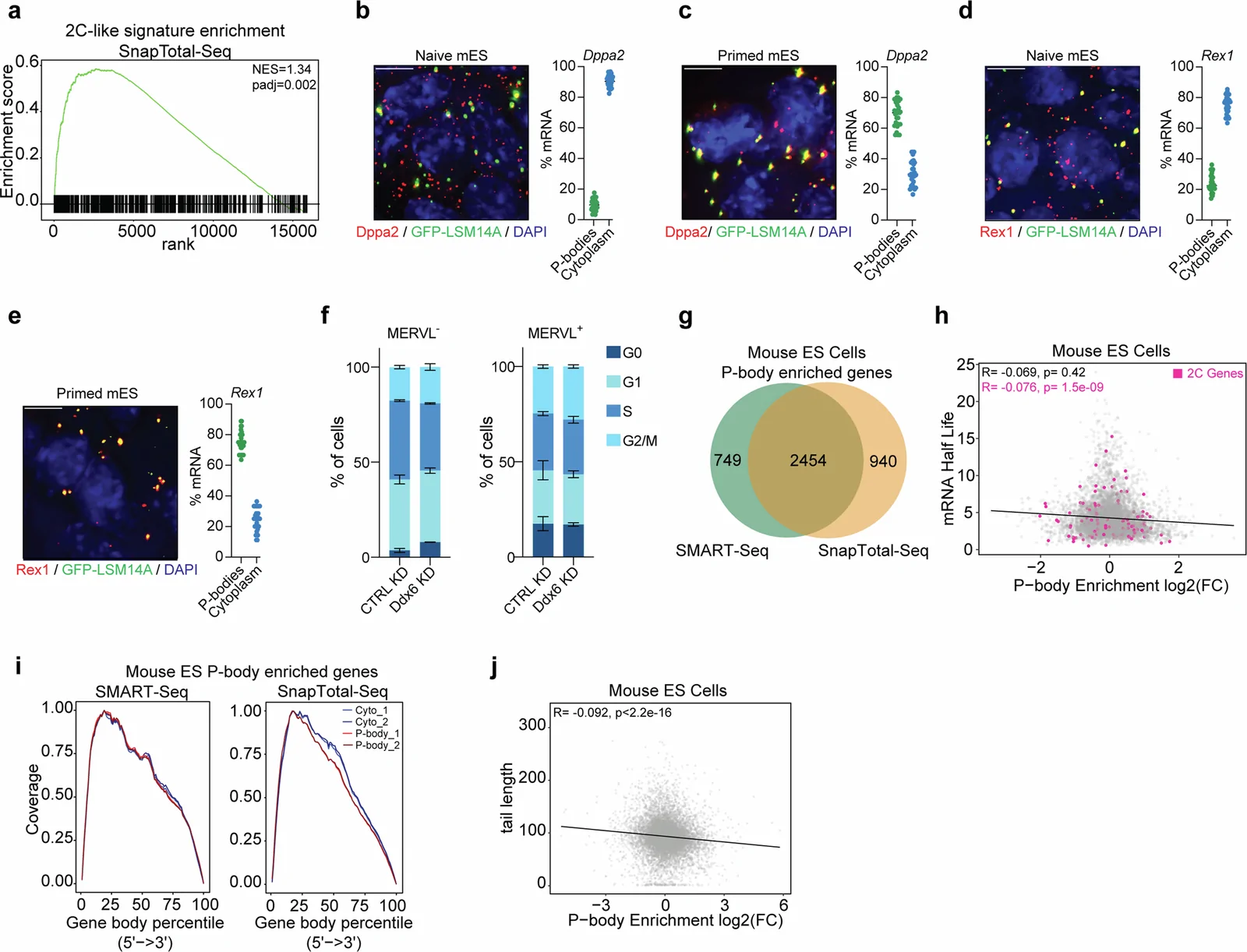
P-body-enriched RNAs in mouse ES cells are not preferentially degraded. (**a**) GSEA for blastomere-related transcripts^63^ in P-body vs cytoplasm differential enrichment data from mouse ES cells using SnapTotal-Seq. Enrichment significance was calculated by permutation test (two-sided), with multiple testing correction using the Benjamini–Hochberg method. (**b, c**) Representative FISH imaging of *Dppa2* RNA molecules (red) combined with imaging of GFP-LSM14A puncta (green) in naïve (**b**) and primed (**c**) ES cells with corresponding quantification (n=30 cells; right). Nuclei were counterstained with DAPI (blue) (scale: 5μm). (**d, e**) Representative FISH imaging of *Rex1* (*Zfp42*) RNA molecules (red) combined with imaging of GFP-LSM14A puncta (green) in naïve (**d**) and primed (**e**) ES cells with corresponding quantification (n=30 cells; right). Nuclei were counterstained with DAPI (blue) (scale: 5μm). (**f**) Cell cycle analysis of MERVL-negative and -positive cells after *Ddx6* KD. n=3 biologically independent samples per group, mean ± s.d. (**g**) Venn diagram showing the overlap between P-body-associated mRNAs in ES cells from SMART-Seq and SnapTotal-Seq. (**h**) mRNA half-life as determined in^66^ compared to P-body enrichment in primed mouse ES cells, 2C genes^63^ are highlighted in pink, Pearson correlation test (two-sided). (**i**) Read coverage distribution over the gene body of the longest annotated isoforms of genes enriched in P-bodies in naïve mouse ES cells using SMART-Seq and SnapTotal-Seq. (**j**) Poly-A tail length as determined in^67^ compared to P-body enrichment based on SMART-Seq and SnapTotal-Seq, Pearson correlation test (two-sided), p=2.35e-69.

In human naive pluripotent stem cells, we found that P-bodies sequester transcripts related to the 8C stage embryo (Fig. 2l and Extended Data Fig. 3l). In mice, the equivalent developmental stage occurs in the two-cell embryo, although rare cells in naive mouse ES cell cultures, known as 2C cells, transiently express genes characteristic of the two-cell state^63^. To determine whether transcripts enriched in the P-bodies of mouse naive ES cells comprise a signature characteristic of totipotent cells, we performed GSEA. Consistent with our findings in human naive ES cells, we observed a significant enrichment of 2C-associated transcripts among P-body-enriched mRNAs, regardless of library preparation method (Fig. 3f and Extended Data Fig. 5a). smFISH analysis confirmed that the naive pluripotency markers *Dppa2* and *Zfp42* were predominantly cytoplasmic in naive ES cells but primarily P-body enriched in primed ES cells (Extended Data Fig. 5b–e). This observation further indicates that P-body-based regulation of developmental processes is conserved between species.

To test the functional consequence of sequestration of 2C transcripts, we employed CRISPR–Cas9 to KO *Ddx6* and dissolve P-bodies in mouse ES cells harboring the 2C-specific reporters MERVL-tdTomato and *Zscan4c*-GFP^64,65^ (Fig. 3g, schematic). *Ddx6* depletion significantly increased signal from both reporters (Fig. 3h,i) without altering cell proliferation (Extended Data Fig. 5f), suggesting that disrupting P-bodies facilitates the otherwise rare conversion of naive ES cells to the 2C state. RNA sequencing (RNA-seq) of MERVL and *Zscan4* double-positive cells confirmed increased expression of genes (*Obox3*, *Zscan4b*, *Zscan4d*, *Zscan4e* and *Zfp352*) and transposable elements (MERVL, MT2B1, MLT2E) characteristic of the 2C state following *Ddx6* suppression (Fig. 3j,k), suggesting that P-body dissolution enhances cell fate conversion. KO of other essential P-body factors, encoded by *Eif4enif1* and *Lsm14a*^32^, similarly led to P-body loss and activation of the MERVL and *Zscan4* reporters (Fig. 3h,i), supporting our hypothesis that P-bodies sequester fate-instructive 2C transcripts that are transiently suppressed and enter translation upon P-body disruption.

**Fig. 4:**
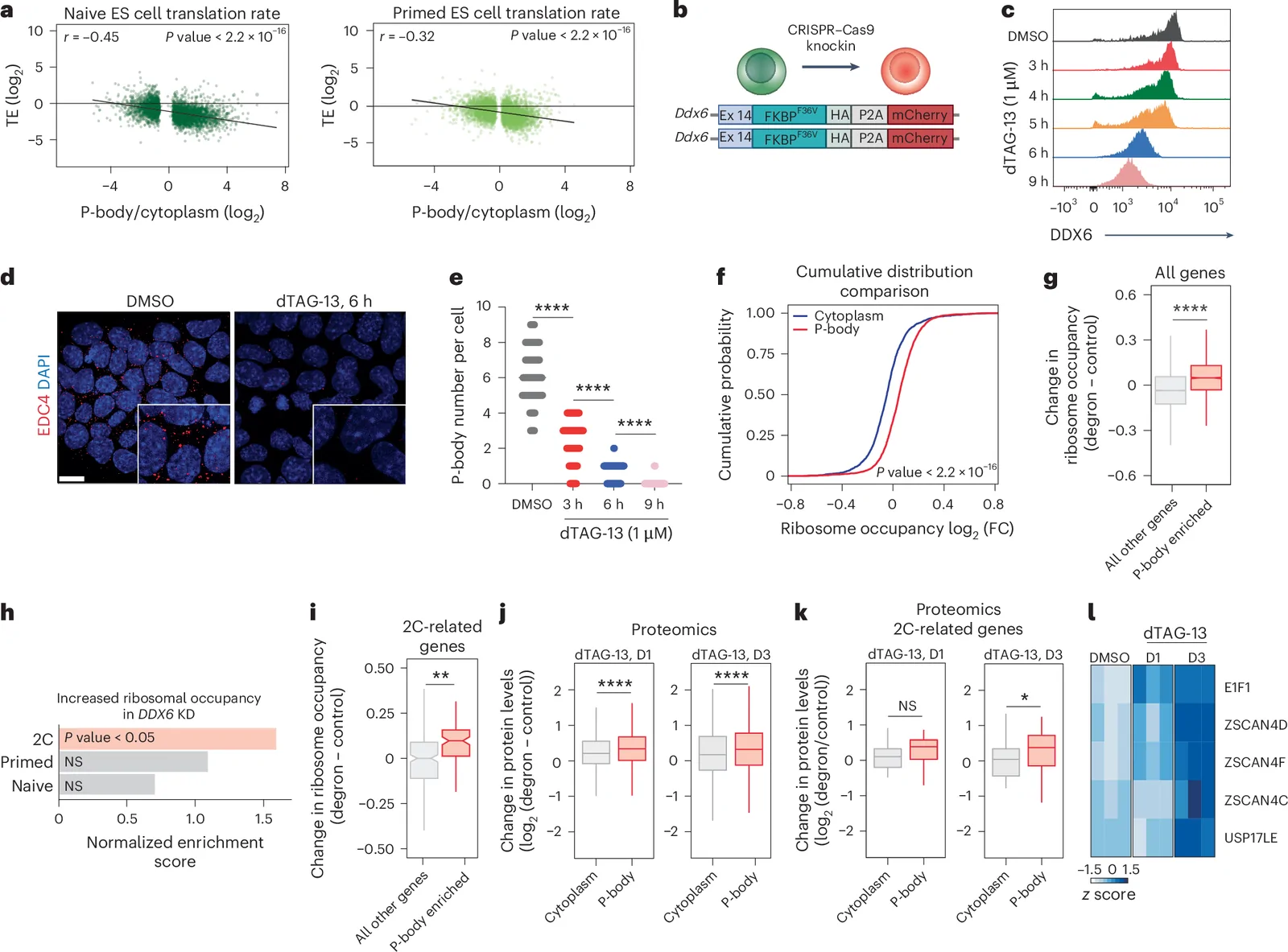
Transcripts related to the 2C state are sequestered in P-bodies, preventing their translation and activation of a 2C gene expression program. **a**, Translation efficiency (log_2_ (Ribo-seq counts/RNA-seq counts)) negatively correlates with mRNA enrichment in P-bodies in naive and primed mouse ES cells. Ribosome profiling data are from ref. ^68^, Pearson correlation test (two-sided). **b**, Schematic showing CRISPR–Cas9-based homozygous insertion of sequence for FKBP12^F36V^-HA-P2A-mCherry in place of the stop codon of the endogenous *Ddx6* allele. Ex, exon. **c**, Representative intracellular flow cytometry plots for DDX6 in *Ddx6*-FKBP12^F36V^ GFP-LSM14A-expressing mouse naive ES cells, either untreated (dimethylsulfoxide; DMSO) or treated with dTAG-13 at the indicated time points. **d**, Representative IF imaging of EDC4 puncta (red) in *Ddx6*-FKBP12^F36V^ GFP-LSM14A-expressing mouse naive ES cells, either untreated (DMSO) or treated with dTAG-13 for 6 h. Nuclei were counterstained with DAPI (blue) (scale bar, 10 μm). **e**, P-body number in *Ddx6-*FKBP12^F36V^ GFP-LSM14A-expressing mouse naive ES cells, either untreated (DMSO) or treated with dTAG-13 at the indicated time points. DMSO, *n* = 70 cells; 3 h, dTAG-13, *n* = 70 cells; 6 h, dTAG-13, *n* = 70 cells; 9 h, dTAG-13, *n* = 70 cells; unpaired two-sided Student’s *t*-test, median ± s.d., ****P < 0.0001. **f**, Cumulative distribution function plot showing ribosome occupancy (log_2_ (FC)) of P-body-enriched and P-body-depleted mRNAs for untreated (DMSO) versus dTAG-13-treated (6 h) *Ddx6-*FKBP12^F36V^ GFP-LSM14A-expressing mouse naive ES cells; Wilcox test *P* = 2.97 × 10^−129^. **g**, Box plots showing the change in ribosome occupancy (log_2_ (ribosome-bound/total RNA) in the degron, log_2_ (ribosome-bound/total RNA) in the control) of P-body-enriched genes versus all other genes. Boxes indicate the IQR (25th–75th percentiles), center lines show the median, and whiskers extend to 1.5× IQR. Statistical significance was assessed using unpaired two-sided *t*-test (mean ± s.d., ****P < 0.0001; *P*_adj_ = 1.6 × 10^−38^ (Holm’s method); all other genes, *n* = 9,133; P-body, *n* = 2,843). **h**, Normalized enrichment score of gene sets from 2C^63^ (*P* = 0.003), naive^104^ (*P* = 0.923) and primed^69^ (*P* = 0.499) samples. Enrichment significance was calculated with the permutation test (two-sided), with multiple-testing correction using the Benjamini–Hochberg method (FDR < 0.05). **i**, Box plots showing the change in ribosome occupancy of P-body-enriched 2C-related genes^63^ compared to non-P-body-enriched 2C genes. Boxes indicate the IQR (25th–75th percentiles), center show lines the median, and whiskers extend to 1.5× IQR. Unpaired two-sided *t*-test, mean ± s.d., **P < 0.01; *P*_adj_ = 0.044 (Holm’s method); all other genes, *n* = 254; P-body, *n* = 104. **j**, Box plots showing the change in protein levels (log_2_ (degron/control)) of all P-body-enriched genes compared to P-body-depleted genes (cytoplasm) after 1 d and 3 d (D1 and D3) of dTAG-13 treatment. Boxes indicate the IQR (25th–75th percentiles), center lines show the median, and whiskers extend to 1.5× IQR. Unpaired two-sided *t*-test, mean ± s.d., ****P < 0.0001; *P*_adj_ = 8.5 × 10^−7^, *P*_adj_ = 6.6 × 10^−6^ (Holm’s method); cytoplasm, *n* = 1,090; P-body, *n* = 1,661. **k**, Box plots showing the change in protein levels log_2_ (degron/control) of P-body-enriched 2C-related genes compared to P-body-depleted genes (cytoplasm) after 1 d and 3 d of dTAG-13 treatment. Boxes indicate the IQR (25th–75th percentiles), center lines show the median, and whiskers extend to 1.5× IQR. Unpaired two-sided *t*-test, mean ± s.d.; not significant, *P* > 0.05; *P < 0.05; *P*_adj_ = 0.086, *P*_adj_ = 0.047 (Holm’s method); cytoplasm, *n* = 28; P-body, *n* = 42. **l**, Heatmap showing protein levels of 2C-related genes after 1 d and 3 d of dTAG-13 treatment compared to control samples.

To explore this possibility further, we assessed the integrity of P-body-associated transcripts in ES cells, including those encoding 2C regulators. Analysis of both snapTotal-seq and Smart-seq data revealed over 70% overlap in P-body-associated transcripts (Extended Data Fig. 5g) between methods. Further comparison between P-body enrichment and mRNA half-lives in ES cells based on 4-thiouridine labeling^66^ demonstrated minimal correlation between mRNA stability and P-body enrichment (*r* = −0.069) (Extended Data Fig. 5h). We found no appreciable evidence of truncated mRNAs or preferential deadenylation^67^ of P-body-associated RNAs compared to cytosolic fractions (*r* = −0.092) (Extended Data Fig. 5i,j), consistent with previous reports^26,29,38^. These findings suggest that, in ES cells, similar to our observations in HEK293T cells, P-body-associated transcripts are not preferentially degraded compared to cytosolic transcripts. We then examined the translation rate of RNAs within P-bodies of mouse ES cells^68,69^. We observed a strong, inverse correlation between P-body enrichment and translation efficiency, suggesting that RNAs localized to P-bodies in naive and primed ES cells are characterized by poor translation efficiency (Fig. 4a). These results indicate that P-body-associated RNAs in ES cells are intact and translationally suppressed.

**Extended Data Fig. 6:**
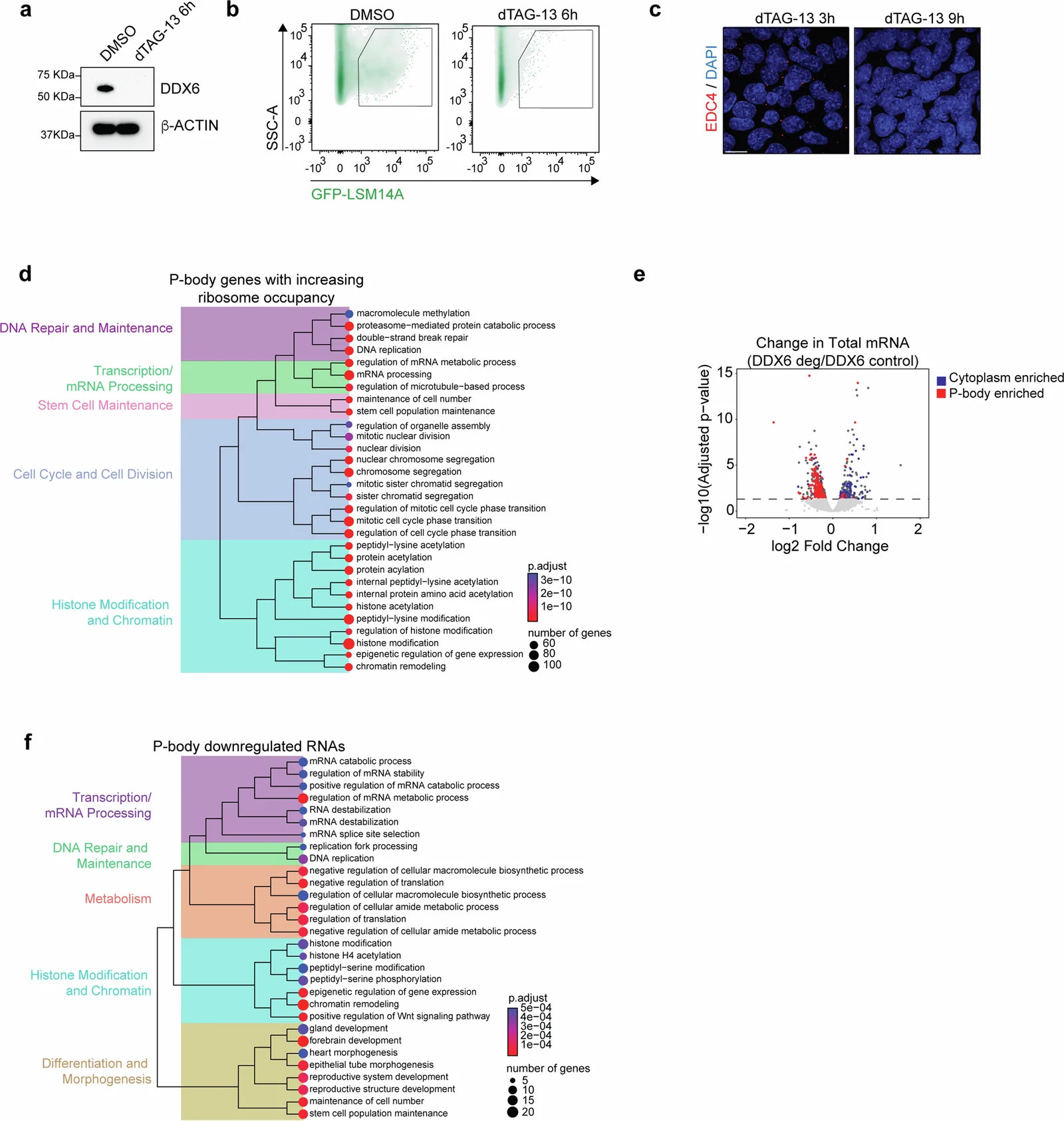
A degron system facilitates acute disruption of P-bodies. (**a**) Representative western blot showing DDX6 protein levels in *Ddx6*-FKBP12^F36V^ GFP-LSM14A mouse naïve ES cells, either untreated (DMSO) or treated with dTAG-13 for 6 h. n=3 independent experiments. (**b**) Representative flow cytometry plots showing gating for GFP-LSM14A^+^ P-bodies in *Ddx6*-FKBP12^F36V^ GFP-LSM14A mouse naïve ES cells, either untreated (DMSO) or treated with dTAG-13 for 6 h. (**c**) Representative IF imaging of EDC4 puncta (red) in *Ddx6*-FKBP12^F36V^ GFP-LSM14A mouse naïve ES cells, treated with dTAG-13 for 3 and 9 h. Nuclei were counterstained with DAPI (blue) (scale: 10mm). n=3 independent experiments. (**d**) GO terms for P-body enriched genes with increased ribosome occupancy of *Ddx6-*FKBP12^F36V^ GFP-LSM14A mouse naïve ES cells following dTAG13 treatment for 6 h). Enrichment was tested by two-sided Fisher’s exact test with multiple testing correction (Benjamini–Hochberg method). (**e**) Volcano plot of RNA-seq data depicting differential expression of total RNA fraction in *Ddx6-*FKBP12^F36V^ GFP-LSM14A mouse naïve ES cells following dTAG13 treatment for 6 h, with P-body enriched genes in red and cytoplasm enriched genes in blue (n=3 biologically independent samples per group, p < 0.05). (**f**) GO terms of P-body enriched genes that showed downregulated gene expression in *Ddx6-*FKBP12^F36V^ GFP-LSM14A mouse naïve ES cells following dTAG13 treatment for 6 h. Enrichment was tested by two-sided Fisher’s exact test with multiple testing correction (Benjamini–Hochberg method). Source data

To directly assess whether P-body-enriched mRNAs engage with translational machinery in stem cells after P-body dissolution, we generated a targeted DDX6 degron system in mouse naive ES cells (*Ddx6*-FKBP12^F36V^) (Fig. 4b) and performed polysome profiling after acute DDX6 loss. Addition of degradation tag-13 (dTAG-13) facilitated DDX6 protein degradation and eliminated detectable P-bodies within 6 h (Fig. 4c–e and Extended Data Fig. 6a–c). Consistent with our model, we observed a significant increase in the ribosome occupancy of P-body-associated mRNAs following DDX6 suppression compared to total cytosolic RNAs (*P* < 2.2^−16^; Fig. 4f,g). GO analysis confirmed that transcripts exhibiting enhanced ribosome occupancy were associated with stem cell maintenance (Extended Data Fig. 6d). Given the observation that disrupting P-bodies in mouse naive ES cells facilitated conversion to the 2C state, we next asked whether 2C-related transcripts demonstrated higher ribosome occupancy after DDX6 degradation. Indeed, 2C-associated mRNAs enriched in P-bodies exhibited increased ribosome occupancy after P-body dissolution (Fig. 4h,i). These data provide further evidence that mRNAs sequestered in P-bodies engage translational machinery upon their release from P-bodies in ES cells, which in turn, alters cell fate.

To measure protein-level changes, we performed large-scale, quantitative proteomics in *Ddx6* degron ES cells after dTAG-13 treatment for 1 and 3 d. P-body dissolution caused significant upregulation of proteins encoded by P-body-associated mRNAs (Fig. 4j), including regulators of 2C-like cells (Fig. 4k). Elevated levels of these proteins facilitated the transition of ES cells to a 2C-like state, as shown by the increased abundance of totipotency factors such as ZSCAN4C, ZSCAN4D, ZSCAN4F, USP17LE and EIF1 (ref. ^64^) (Fig. 4l). These findings demonstrate that disrupting RNA sequestration in P-bodies promotes the translation of proteins important for cell fate transitions.

In line with previous studies, a fraction of P-body-enriched RNAs underwent degradation after acute loss of DDX6 (Extended Data Fig. 6e). These downregulated mRNAs were linked to differentiation processes (Extended Data Fig. 6f), perhaps reflecting a separate mechanism for protecting transcripts needed for differentiation until appropriate signals or conditions are achieved during development^70^.

### miRNAs direct cell type-specific sequestration of mRNAs into P-bodies

**Extended Data Fig. 7:**
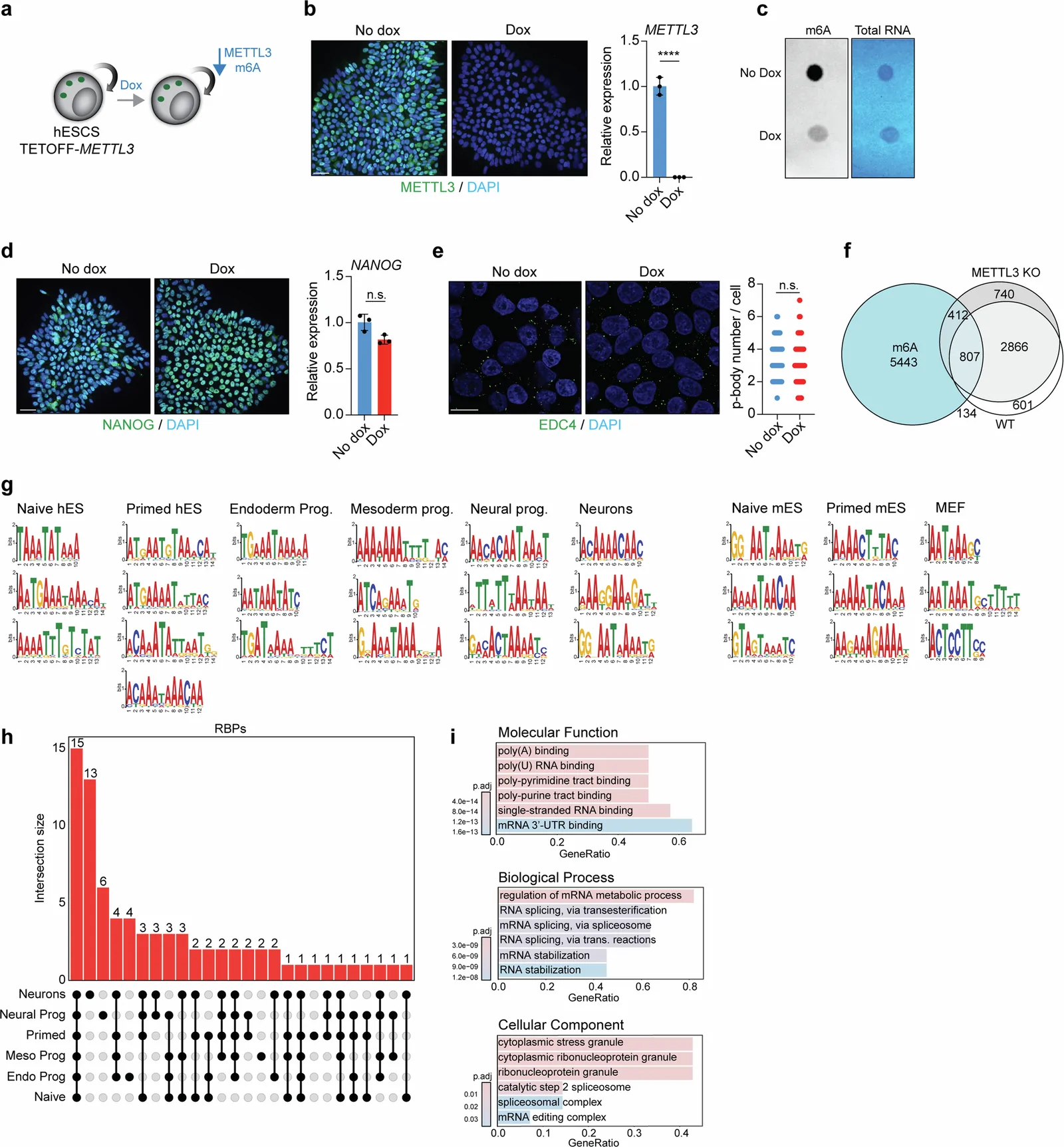
m6A RNA modification and RBPs do not drive cell-type specific transcript sequestration. (**a**) A schematic of the strategy for METTL3 suppression in TETOFF-*METTL3* human ES cells. (**b**) Representative IF imaging of METTL3 (green) in control (no dox) and METTL3 KO induced (dox) ES cells. Nuclei were counterstained with DAPI (blue) (scale: 50μm) (left panel). qRT-PCR analysis of *METTL3* expression in control (no dox) and METTL3 KO induced (dox) ES cells. Unpaired two-sided Student’s t-test, n=3 biologically independent samples per group, mean ± s.d., ****: p<0.0001 (right panel). (**c**) m6A RNA dot blot in in control (no dox) and METTL3 KO induced (dox) ES cells. Methylene Blue staining was used as loading control. (**d**) Representative IF imaging of NANOG (green) in control (no dox) and METTL3 KO induced (dox) ES cells. Nuclei were counterstained with DAPI (blue) (scale: 50μm) (left panel). qRT-PCR analysis of *NANOG* expression in control (no dox) and METTL3 KO induced (dox) ES cells. Unpaired two-sided Student’s t-test, n=3 biologically independent samples per group, mean ± s.d., n.s.: p>0.05; p=0.5542 (**e**) Representative IF imaging of EDC4 puncta (green) in control (no dox) and METTL3 KO induced (dox) ES cells. Nuclei were counterstained with DAPI (blue) (scale: 10μm) (left panel). P-body number in control (no dox, n=56) and METTL3 KO induced (dox, n=56) ES cells (right panel). Unpaired two-sided Student’s t-test, median ± s.d., n.s.: p>0.05; p=0.3805. (**f**) Venn diagram showing the overlap between P-body-associated mRNAs in WT (no dox) and METTL3 KO induced (dox) ES cells, and m6A-methylated mRNAs in human ES cells. (**g**) Motifs enriched in the top 100 P-body enriched genes for each cell type, determined using STREME. (**h**) Upset plot of the RNA Binding Proteins that were predicted to bind the motifs identified in (**g**) for each human cell type, determined by Tomtom with the Ray 2013 database. (**i**) GO terms for proteins that recognize the enriched motifs in all human cell types. Enrichment was tested by two-sided Fisher’s exact test with multiple testing correction (Benjamini–Hochberg method).

Our data suggest cell type-specific sequestration of mRNA into P-bodies, yet the underlying mechanism remained unclear. We first considered RNA modifications, focusing on *N*^6^-methyladenosine (m^6^A), the most abundant modification in eukaryotic mRNA^71^. We used a human ES cell model that permits doxycycline-inducible ablation of the m^6^A methyltransferase METTL3 (TET-OFF *METTL3*)^72^ (Extended Data Fig. 7a). Eight days of doxycycline treatment suppressed METTL3 expression (Extended Data Fig. 7b) and reduced global m^6^A levels (Extended Data Fig. 7c), while pluripotency genes remained unaffected (Extended Data Fig. 7d). IF for EDC4^+^ puncta revealed no change in P-body numbers upon *METTL3* deletion (Extended Data Fig. 7e). We profiled P-body contents and found no significant differences in *METTL3*-KO cells compared to the control (85% overlap; Extended Data Fig. 7f; compare white and gray circles). Moreover, only a small fraction of P-body-enriched RNA was m^6^A methylated, and enrichment remained largely unchanged after *METTL3* KO (Extended Data Fig. 7f). These results indicate that m^6^A methylation does not primarily direct RNA sequestration into P-bodies in human ES cells, consistent with findings on stress granules in mouse ES cells^73^.

**Fig. 5:**
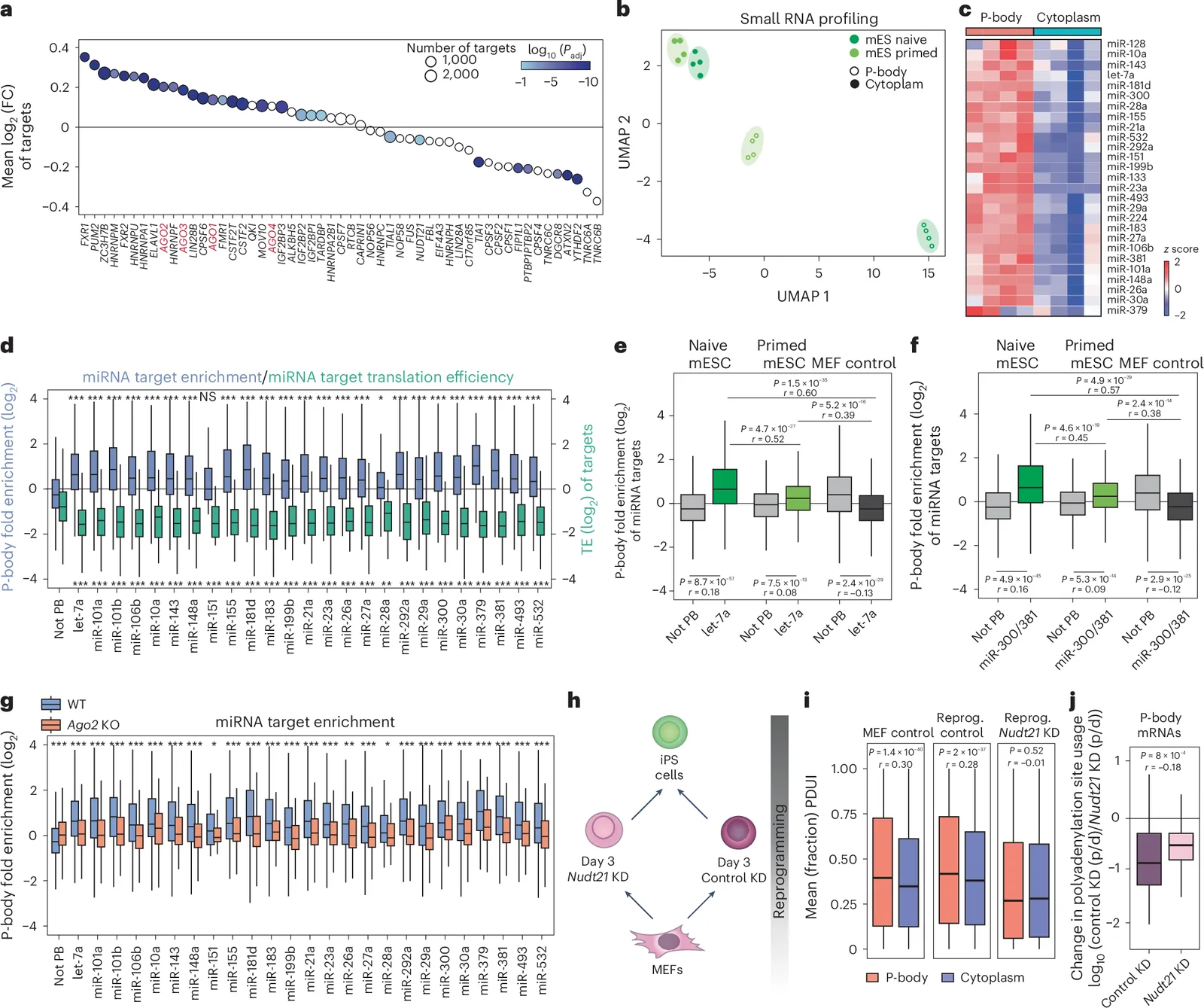
miRNAs regulate RNA sequestration in a context-dependent manner. **a**, mRNA targets of 53 RBPs were analyzed for P-body enrichment in HEK293T cells using CLIP–seq data summarized in ref. ^29^. Circle size reflects the number of targets per RBP. Color indicates Benjamini–Hochberg *P*_adj_ from a two-sided Wilcoxon test comparing target log_2_ (FC) values to those of all genes not bound by that RBP. AGO (Argonaute) protein family members are highlighted in red. *C17orf85*, *NCBP3*. *PTBP1PTBP2*, *PTBP1* and *PTBP2*. **b**, Uniform manifold approximation and projection (UMAP) analysis of small RNA-seq data for the indicated samples based on differentially expressed genes between purified P-body and cytoplasmic fractions. mES, mouse ES cell. **c**, Heatmap showing expression levels of selected miRNAs in P-body and cytoplasmic fractions in mouse naive ES cells (*n* = 4 biological independent samples per group). **d**, Quantification of P-body enrichment for miRNA targets (blue) and their corresponding translation efficiency (green) from mouse naive ES cells. Ribosome profiling data are from ref. ^68^. Boxes indicate the IQR (25th–75th percentiles), center lines show the median, and whiskers extend to 1.5× IQR. Unpaired two-sided Wilcoxon test compares the targets of each miRNA to all genes that are not targets of P-body-enriched miRNA (PB) with Benjamini–Hochberg adjustment (*P < 0.05, **P < 0.01, ***P < 0.001; *n* and exact *P* values are noted in the source data). **e**,**f**, Box plots showing P-body enrichment of let-7a (let-7a targets, *n* = 395; non-P-body targets, *n* = 5,275) (**e**) and miR-300 and miR-381 (miR-300 and miR-381 targets, *n* = 353; non-P-body targets, *n* = 5,275) (**f**) targets in naive and primed mouse ES cells and MEFs. Unpaired Wilcoxon tests compare target genes to nontargets within each cell type (Benjamini–Hochberg *P*_adj_ values and Wilcoxon *r* effect size reported in the figure). Boxes indicate the IQR (25th–75th percentiles), center lines show the median, and whiskers extend to 1.5× IQR. Paired, two-sided Wilcoxon tests compare enrichment between cell types (Benjamini–Hochberg *P*_adj_ values and Wilcoxon *r* effect size are shown in the figure). **g**, Box plot comparing P-body enrichment of miRNA targets between WT and *Ago2*-KO mouse naive ES cells. Boxes indicate the IQR (25th–75th percentiles), center lines show the median, and whiskers extend to 1.5× IQR. Paired, two-sided Wilcoxon test with Benjamini–Hochberg *P*_adj_ values; *P < 0.05, **P < 0.01, ***P < 0.001; NS, *P* > 0.05; *n* and exact *P* values are noted in the source data. **h**, Schematic of induced Pluripotent Stem (iPS) cell reprogramming, including perturbation of the APA regulator NUDT21. **i**, PolyA site usage index (PDUI) of P-body- and cytoplasm-enriched transcripts. PDUI < 0.5 indicates proximal polyA preference; PDUI > 0.5 indicates distal polyA preference. Boxes indicate the IQR (25th–75th percentiles), center lines show the median, and whiskers extend to 1.5× IQR. Two-sided Wilcoxon test, Benjamini–Hochberg *P*_adj_ values and Wilcoxon *r* effect size are reported in the figure (MEF control, *n* = 1,967; MEF reprogrammed control (reprog. control), *n* = 2,065; *Nudt21*-KD reprogrammed MEF (reprog. *Nudt21* KD), *n* = 2,189). **j**, Change in polyadenylation site usage after *Nudt21* KO from Poly(A) Site sequencing (PAS-seq) data for genes that are lost from *Nudt21*-KO P-bodies compared to genes in the P-bodies of *Nudt21*-KO and control KO cells. p, proximal; d, distal. Boxes indicate the IQR (25th–75th percentiles), center lines show the median, and whiskers extend to 1.5× IQR. Two-sided Wilcoxon test, Benjamini–Hochberg *P*_adj_ values and Wilcoxon *r* effect size are reported in the figure. For all panels in which effect size is reported, *r* < 0.1 is a negligible effect, *r* = 0.1–0.3 is small, *r* = 0.3–0.5 is medium and *r* > 0.5 is a large effect (control KD, *n* = 124; *Nud21* KD, *n* = 404). Source data

We next explored the potential involvement of RBPs in RNA sequestration within P-bodies by mapping the P-body enrichment of transcripts targeted by 53 RBPs using published crosslinking immunoprecipitation followed by sequencing (CLIP–seq) data^74^ (Fig. 5a). Targets of RBPs that localize to RNA granules, including PUM2 and HNRNPU^19,75^, were enriched in P-bodies; however, these proteins are widely expressed and unlikely to confer cell type specificity. De novo motif analysis of P-body-enriched transcripts predicted recognition by multiple RBPs that were conserved across cell types, with no evident cell type-specific patterns (Extended Data Fig. 7g,h). GO analysis of the identified RBP targets revealed enrichment for pathways consistent with known P-body functions, including 3′ untranslated region (3′-UTR) processing, ribonucleoprotein granule assembly and mRNA stabilization (Extended Data Fig. 7i). These data suggest that RBPs are unlikely to drive cell type-specific RNA sequestration.

Further inspection of the CLIP–seq data revealed enrichment for targets of the Argonaute protein family (AGO1, AGO2, AGO3 and AGO4) within P-bodies (Fig. 5a). AGO proteins are essential components of the RNA-induced silencing complex and cooperate with miRNAs to suppress mRNA translation^76,77^. Previous studies have shown localization of AGO proteins in P-bodies and suggested a connection between certain miRNAs and mRNA recruitment to ribonucleoprotein granules in nonmammalian cells and transformed cell lines^23,78–80^. Moreover, miRNAs are expressed in a cell type-specific manner and play a prominent role in cell fate transitions^81,82^.

**Extended Data Fig. 8:**
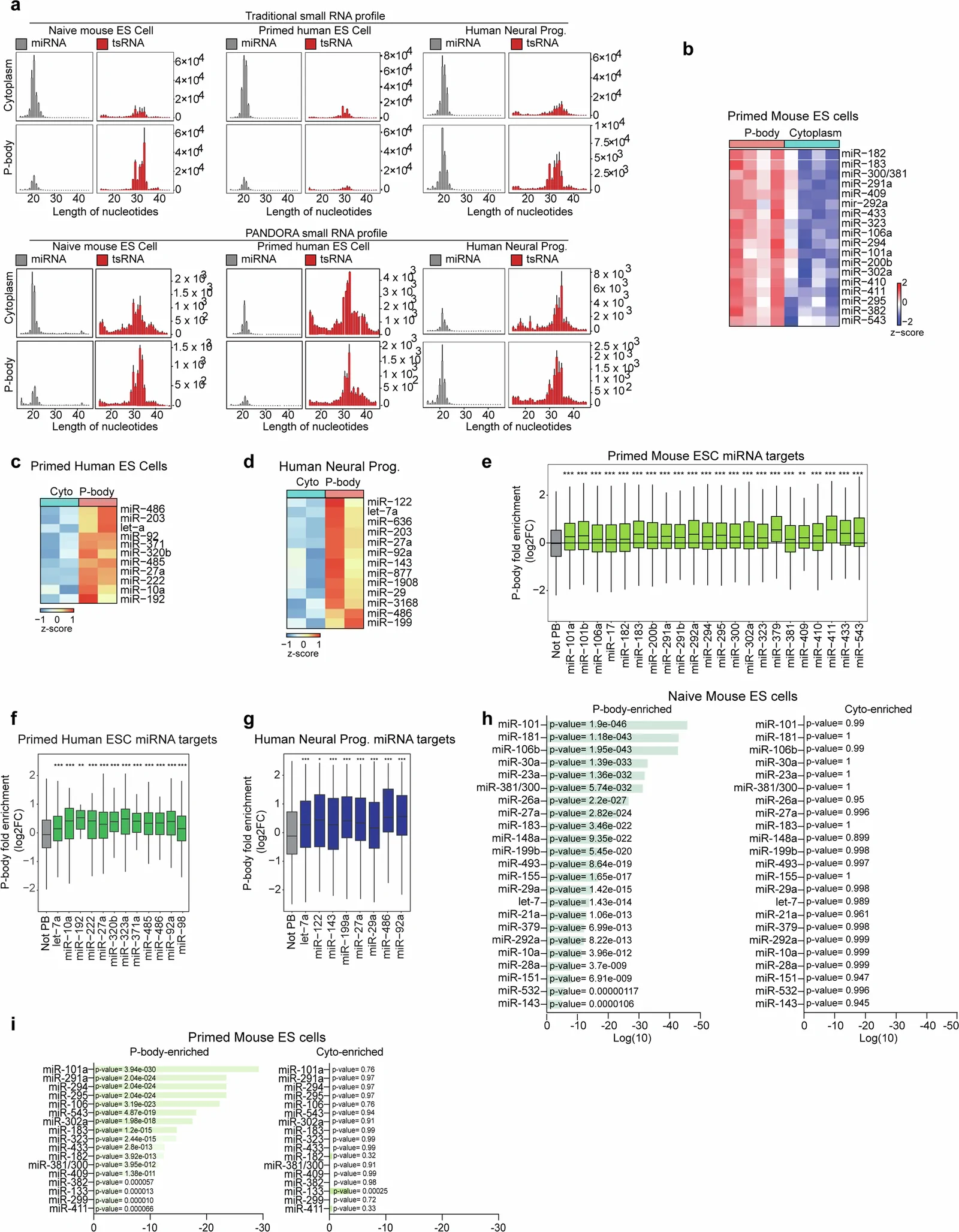
miRNAs direct selective sequestration of transcripts into P-bodies. (**a**) Changes in traditional (upper panel) and PANDORA (lower panel) small RNA distribution between purified P-body and cytoplasmic fractions in the indicated samples. (**b**) Heatmap showing expression levels of selected miRNAs in P-body and cytoplasmic fractions in mouse naïve ES cells. (n=4 biological independent samples per group). (**c**) Heatmap showing expression levels of selected miRNAs in P-body and cytoplasmic fractions in primed human ES cells. (n=2 biological independent samples per group). (**d**) Heatmap showing expression levels of selected miRNAs in P-body and cytoplasmic fractions in human neural progenitors. (**e**) Box plot showing P-body enrichment of targets of miRNAs enriched in P-bodies of mouse primed ES cells. Boxes indicate the interquartile range (25th–75th percentile), center lines the median, and whiskers extend to 1.5X IQR. Unpaired two-sided Wilcoxon tests comparing the targets to all genes that are not targets of P-body enriched miRNA, with Benjamini–Hochberg adjustment, *: p<0.05, **: p<0.01, ***p<0.001, n and exact p-values are reported in Source Data ED Fig 8. (**f, g**) Box plot showing P-body enrichment of targets of miRNAs enriched in P-bodies of primed ES cells (**f**) and human neural progenitors (**g**). Boxes indicate the interquartile range (25th–75th percentile), center lines the median, and whiskers extend to 1.5X IQR. Unpaired two-sided Wilcoxon tests compare the targets to all genes that are not targets of P-body enriched miRNA, with Benjamini–Hochberg adjustment, *: p<0.05, **: p<0.01, ***: p<0.001, n and exact p-values are reported in Source Data ED Fig 8 (**h, i**) MIENTURNET predictions for miRNA targeting specifically P-body-enriched mRNA in naïve (**h**) and primed (**i**) mouse ES cells. P-values were calculated using the hypergeometric test implemented in MIENTURNET. Source data

**Extended Data Fig. 9:**
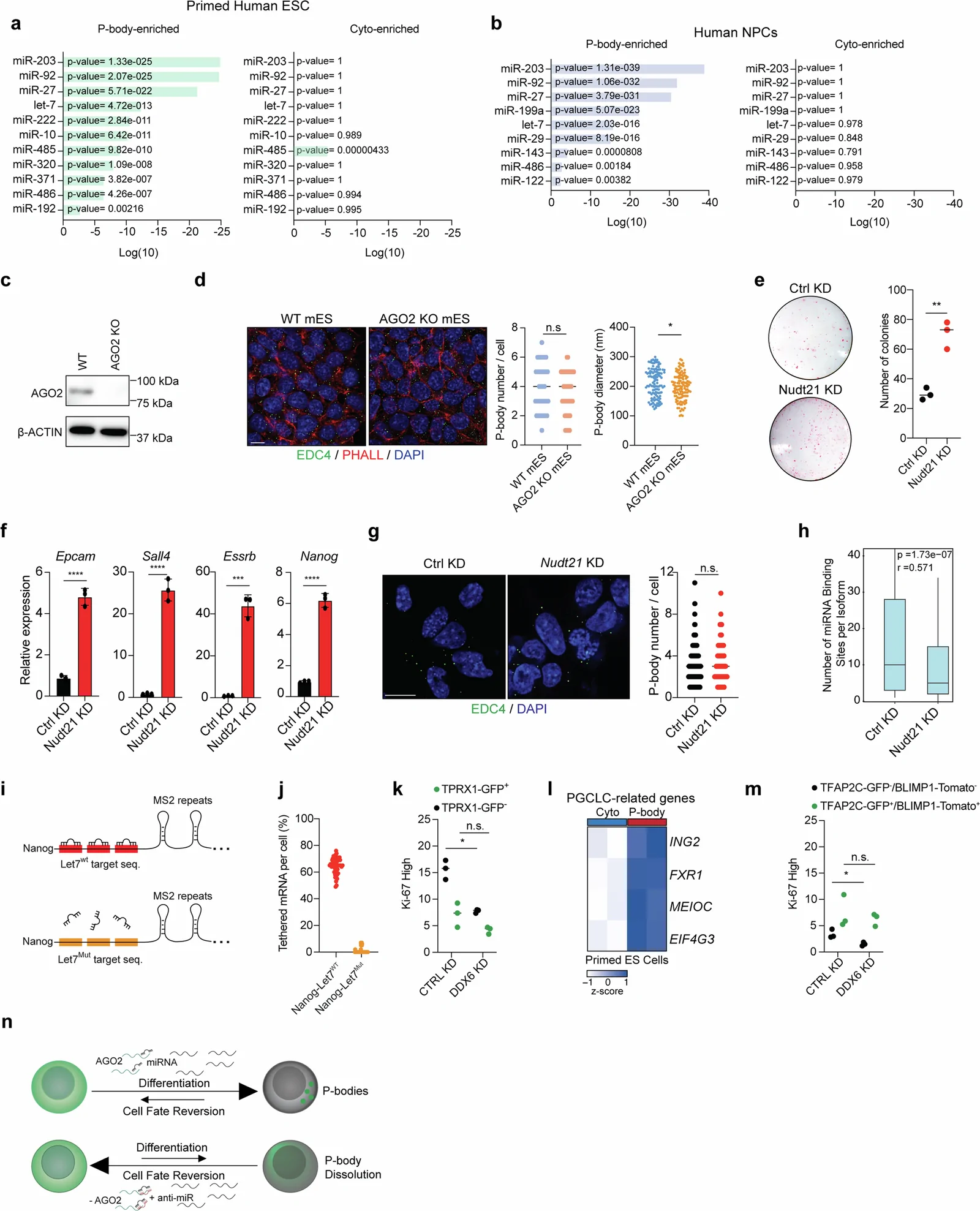
Uncoupling miRNA-mRNA interaction disrupts enrichment of transcripts into P-bodies. (**a, b**) MIENTURNET predictions for miRNA targeting specifically P-body-enriched mRNA in primed human ES (**a**) and neural progenitors (**b**). P-values were calculated using the hypergeometric test implemented in MIENTURNET. (**c**) Representative western blot showing AGO2 protein levels in WT and AGO2 KO mouse naïve cells. (**d**) Representative IF images of EDC4 puncta (green) in naïve and primed mouse ES cells. Cell membranes were labeled with Phalloidin (red), and nuclei were counterstained with DAPI (blue) (scale: 10μm) (left panel). P-body number and size quantification in WT (n=60 cells) and AGO2 KO (n=60 cells) mouse naïve ES cells (right panel). Unpaired two-sided Student’s t-test, median ± s.d., *: p<0.05, n.s.: p>0.05; p=0.1720, p=0.0113. (**e**) Representative images and quantification of Alkaline Phosphatase-positive, transgene-independent iPSC colonies in control and *Nudt21* knockdown cells at day 15 of reprogramming. Unpaired two-sided Student’s t-test, n=3 biologically independent samples per group, mean ± s.d., **: p<0.01; p=0.0022. (**f**) qRT-PCR analysis of the expression of ES-specific genes in control and *Nudt21* KD reprogramming samples. Unpaired two-sided Student’s t test, n=3 biologically independent samples per group, mean ± s.d., ***: p<0.001, ****: p<0.0001; p=0.0001. (**g**) Representative IF imaging of EDC4 puncta (green) in control and *Nudt21* KD reprogramming samples. Nuclei were counterstained with DAPI (blue) (scale: 10μm) and P-body number in control (n=90 cells) and *Nudt21* KD induced (dox, n=90 cells) reprogramming samples (right panel). Unpaired two-sided Student’s t-test, mean ± s.d., n.s.: p>0.05; p=0.1549. (**h**) The number of miRNA binding sites per gene isoform for genes that are lost from Nudt21 knockdown P-bodies but retained in Control knockdown P-bodies, in the most highly expressed isoform in each condition. Boxes indicate the interquartile range (25th–75th percentile), center lines the median, and whiskers extend to 1.5X IQR. Paired two-sided Wilcoxon test, with Benjamini–Hochberg-adjusted p-value and Wilcoxon r; r<0.1 is a negligible effect, 0.1-0.3 is small, 0.3-0.5 is medium, and >0.5 is a large effect. (Ctrl KD n=279, Nudt21 KD n=922). (**i**) A schematic of the strategy for the generation of Let7^wt^ and Let7^mut^ reporter cell lines. (**j**) Quantification of tethered *Nanog* mRNA from Nanog Let7^wt^ (n=50 cells) and Nanog Let7^mut^ cells (n=50 cells). (**k**) Ki67 levels in TPRX1-negative and -positive cells. Unpaired two-sided Student’s t test, n=3 biologically independent samples per group, median ± s.d., *: p<0.05, n.s.: p>0.05; p=0.0147, p=0.1474. (**l**) Heatmap showing expression levels of selected PGCLC-related mRNAs in P-body and cytoplasmic fractions in human primed ES cells (n=2 biologically independent samples per group). (**m**) Ki67 levels in TFAP2C/BLIMP1-negative and -positive cells. Unpaired two-sided Student’s t test, n=3 biologically independent samples per group, median ± s.d., *: p<0.05, n.s.: p >0.05; p=0.0434, p=0.6161. (**n**) A model showing miRNA-mediated RNA sequestration in cell fate. Source data

To test the role of miRNAs in directing RNA sequestration of developmental transcripts into P-bodies, we profiled small RNA populations in both P-body and cytoplasmic fractions across mouse and human pluripotent cells and neural progenitors using small noncoding RNA-seq^83^ (Extended Data Fig. 8a). Uniform manifold approximation and projection plot analysis revealed clustering of naive and primed mouse ES cell samples according to their cellular origin (Fig. 5b). Subsets of miRNAs were selectively enriched in P-bodies of pluripotent cells as well as progenitor cells (Fig. 5c and Extended Data Fig. 8b–d), including miR-300 and miR-let-7, which are known regulators of stem cell potency^84,85^. Targets of miRNAs enriched in P-bodies were also sequestered within these RNA condensates (Fig. 5d and Extended Data Fig. 8e–g) and translationally repressed (Fig. 5d). Further analysis^86^ revealed that P-body-targeted transcripts were enriched for specific miRNA binding sites that were absent in cytoplasmic transcripts (Extended Data Figs. 8h,i and 9a,b).

We plotted P-body enrichment for targets of miR-let-7, miR-300 and miR-381 across cell types. In both naive and primed ES cells, we observed increased enrichment of their targets in P-bodies (Fig. 5e,f). By contrast, we found no enrichment for target transcripts in mouse embryonic fibroblast (MEF) P-bodies (Fig. 5e,f). Together, our data suggest that miRNA-targeted genes are sequestered into P-bodies in a cell type-specific manner.

### Disrupting miRNA function prevents RNA sequestration in P-bodies

Previous studies demonstrated the importance of AGO2 in the sequestration of let-7 targets into P-bodies in transformed cells^78^. To investigate the involvement of AGO2 in global RNA sequestration, we generated *Ago2*-KO mouse ES cells^87^ (Extended Data Fig. 9c). *Ago2*-KO cells had similar numbers of P-bodies as wild-type (WT) cells (Extended Data Fig. 9d), although P-bodies appeared modestly smaller (Extended Data Fig. 9d). We then sorted P-bodies from *Ago2*-KO ES cells and compared their transcriptome to WT counterparts. We found that *Ago2* KO significantly reduced miRNA-targeted mRNA levels in P-bodies (Fig. 5g).

Next, we investigated whether modulation of polyA site usage (that is, alternative polyadenylation (APA)) influenced RNA sequestration into P-bodies. APA generates RNA isoforms that commonly differ in the length of their 3′ UTR^88^. Because miRNA target sequences are frequently found in the 3′ UTR, we reasoned that forcing proximal polyadenylation (that is, shorter 3′ UTRs) would relieve miRNA-based targeting of transcripts into P-bodies. To test this, we suppressed expression of *Nudt21*, encoding a component of the CFIm complex that facilitates distal polyadenylation^5,89^, in MEFs during reprogramming to pluripotency (Fig. 5h). We chose this context because a matched dataset of APA changes is available^5^, permitting direct comparison between polyA site usage and P-body enrichment. *Nudt21* knockdown (KD) led to increased reprogramming efficiency (Extended Data Fig. 9e) and expression of pluripotency-related genes^5^ (Extended Data Fig. 9f). Similar to *Ago2* KO, P-body numbers were largely unaffected by *Nudt21* suppression (Extended Data Fig. 9g). P-body-seq from uninduced MEFs as well as reprogramming intermediates with and without *Nudt21* silencing revealed that, in MEFs and day 3 control reprogramming cells, 3′ UTRs of P-body-targeted RNAs were longer than cytoplasmic counterparts, suggesting that regulatory sequences within 3′ UTRs mediate P-body enrichment (Fig. 5i). After *Nudt21* suppression, we observed a global reduction in 3′ UTR length and the 3′ UTRs of transcripts enriched in P-bodies had similar lengths relative to those of cytoplasmic transcripts (Fig. 5i). We next used published data to compare changes in polyadenylation site usage to changes in transcript localization between P-bodies and the cytoplasm after *Nudt21* KD^5^. Notably, transcripts enriched in P-bodies of control cells but absent from P-bodies after *Nudt21* KD (that is, genes that relocated away from P-bodies) had shorter 3′ UTRs after *Nudt21* suppression (Fig. 5j). Analysis of previously published polyA site usage data^5^ revealed that these transcripts contained significantly fewer miRNA target sites after *Nudt21* KD (Extended Data Fig. 9h). These results suggest that the loss of miRNA binding sites prevents mRNA localization to P-bodies.

### Disrupting miRNAs relocalizes target transcripts and influences cell identity

**Fig. 6:**
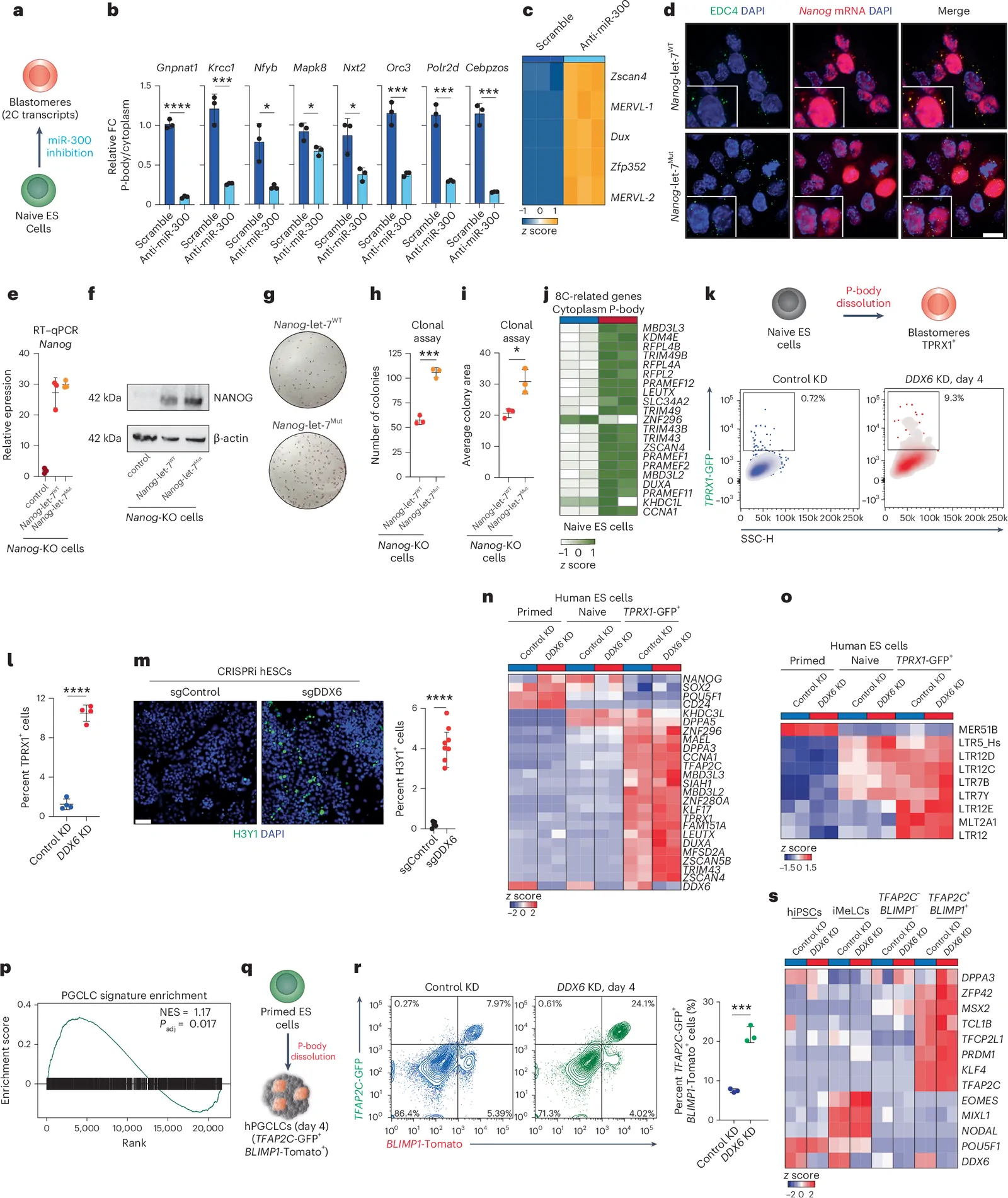
Modulating miRNA function and P-body assembly alters cell fate. **a**, Schematic of the strategy for miR-300 inhibition in mouse naive ES cells. **b**, Quantitative PCR with reverse transcription (RT–qPCR) analysis of expression of miR-300 targets in P-bodies of mouse naive ES cells after miR-300 inhibition and for the control; *n* = 3 biological independent samples per group, unpaired two-sided Student’s *t*-test, mean ± s.d.; *P < 0.05, ***P < 0.001, ****P < 0.0001; *P* = 0.0009, *P* = 0.0133, *P* = 0.0237, *P* = 0.0195, *P* = 0.0009, *P* = 0.0004, *P* = 0.0002. **c**, Heatmap showing expression levels of differentially enriched 2C-related genes in mouse naive ES cells after miR-300 inhibition and for the control. **d**, Representative IF imaging of EDC4 puncta (green) and *Nanog*-MS2 (red) in *Nanog*-let-7^WT^ (top) and *Nanog*-let-7^Mut^ cells (bottom). Nuclei were counterstained with DAPI (blue) (scale bar, 10 μm). *n* = 3 independent experiments. **e**, RT–qPCR analysis of *Nanog* expression in *Nanog*-let-7^WT^ and *Nanog*-let-7^Mut^ cells compared to control cells (*Nanog* KO). *n* = 3 biologically independent samples per group, mean ± s.d. **f**, Representative western blot showing NANOG protein levels in *Nanog*-let-7^WT^ and *Nanog*-let-7^Mut^ cells compared to control cells (*Nanog* KO). **g**–**i**, Representative pictures (**g**) and quantification of alkaline phosphatase staining of cell colony number (**h**) and size (**i**) of *Nanog*-let-7^WT^ and *Nanog*-let-7^Mut^ cells cultured with fetal bovine serum and leukemia inhibitory factor (LIF). Unpaired two-sided Student’s *t*-test, *n* = 3 biologically independent samples per group, mean ± s.d.; *P < 0.05, ***P < 0.001; *P* = 0.0002, *P* = 0.0135. **j**, Heatmap showing expression levels of differentially enriched 8C-related mRNAs between purified P-body and cytoplasmic fractions in human naive ES cells (*n* = 2 biological independent samples per group). **k**, Schematic of the strategy for P-body dissolution in naive human ES cells carrying a blastomere-specific reporter (*TPRX1*-GFP). Flow cytometric analysis of *TPRX1*-GFP^+^ expression after 4 d of *DDX6* KD. k, 1,000. SSC, side scatter. **l**, Quantification of *TPRX1*-GFP^+^ cells upon *DDX6* KD. Unpaired two-sided Student’s *t*-test, *n* = 4 biologically independent samples per group, mean ± s.d., ****P < 0.0001. **m**, Representative IF imaging of H3Y1 (green)-positive cells in naive human ES cells upon *DDX6* KO compared to control cells. Nuclei were counterstained with DAPI (blue) (scale bar, 50 μm) (left). Quantification of H3Y1^+^ cells upon *DDX6* suppression. Unpaired two-sided Student’s *t*-test, single-guide (sg)Control (*n* = 5 fields), sgDDX6 (*n* = 8 fields), mean ± s.d., ****P < 0.0001 (right). hESC, human ES cell. **n**,**o**, RNA-seq data from primed, naive and *TPRX1*-positive human ES cells after *DDX6* KD, showing expression levels of pluripotency- and totipotency-related transcripts (**n**) and transposable elements (**o**). **p**, GSEA analysis for the human PGCLC-related gene expression signature^96^ in P-body-versus-cytoplasmic differential expression in human primed ES cells. Enrichment significance was calculated using the permutation test (two sided), with multiple-testing correction using the Benjamini–Hochberg method. **q**, Schematic of human iPS cell (hiPSC)-to-PGCLC differentiation. hPGCLC, human PGCLC. **r**, Left: flow cytometric analysis of *TFAP2C*-GFP and *BLIMP1*-Tomato expression after 4 d of PGCLC differentiation in three-dimensional aggregates. Right: quantification of *TFAP2C*-GFP^+^ and *BLIMP1*-Tomato^+^ cells by flow cytometry with three experiments. Error bars indicate mean ± s.d., *n* = 3 biologically independent samples per group; statistical significance was determined using a two-sided unpaired Student’s *t*-test; ***P < 0.001, *P* = 0.0003. **s**, RNA-seq analysis of hiPSCs (BTAG cell line^96^), intermediate mesenchymal-like cells (iMeLCs) and *TFAP2C*-and-*BLIMP1*-negative and -positive cells after DDX6 suppression, showing expression levels of pluripotency, mesenchymal and PGC-related genes. Source data

We next examined whether manipulating specific miRNAs could impact RNA localization and cell identity by depleting miR-300 in mouse ES cells using an antisense inhibitor (Fig. 6a). Following 48 h of anti-miR-300 treatment, we observed a significant reduction of miR-300 targets in P-bodies (Fig. 6b).

To test whether releasing miR-300 targets from P-bodies was sufficient to induce a change in cell fate (Fig. 6a), we examined the expression of 2C-related genes. We observed upregulation of 2C-related transcripts, including *Zscan4* and MERVL, upon miR-300 suppression, suggesting that miR-300 restricts reversion of pluripotent cells to an earlier developmental fate (Fig. 6c). Notably, these transcripts are not direct targets of miR-300, suggesting that their upregulation results from a change in cell fate, rather than regulation from the miRNA itself. These findings indicate that RNA sequestration in P-bodies can be manipulated to change cell identity.

We next tested whether mRNAs could be directed into P-bodies through the addition of miRNA target sequences. We generated reporter constructs based on the pluripotency master regulator encoded by *Nanog*, which included a 3′ UTR with either six WT or six mutant let-7 sequences (let-7^WT^ and let-7^Mut^)^90^ and 24 bacteriophage MS2 stem loops (Extended Data Fig. 9i). These stem loops recruit a tagged version of the MS2 coat protein (JF_546_ HaloTag-NLS-MCP) to permit direct, subcellular visualization of *Nanog* mRNA. We note that the nuclear localization signal on MCP effectively clears free MCP from the cytoplasm, strongly increasing the signal-to-noise ratio for the assay^91^. After transfection into *Nanog*-KO ES cells, *Nanog*-let-7^WT^-MS2 transcripts colocalized with the P-body marker EDC4 (Fig. 6d and Extended Data Fig. 9j), suggesting that let-7 target sequences were sufficient to direct *Nanog* mRNA into P-bodies. By contrast, *Nanog*-let-7^Mut^-MS2 transcripts failed to accumulate in P-bodies (Fig. 6d and Extended Data Fig. 9j). Both constructs produced similar steady-state RNA levels (Fig. 6e), excluding the possibility that the observed localization patterns arose from differences in RNA abundance. NANOG protein levels, however, were lower in cells expressing *Nanog*-let-7^WT^-MS2, supporting a nondegradative, posttranscriptional regulatory mechanism (Fig. 6f).

Finally, we investigated whether miRNA-dependent recruitment of RNAs into P-bodies could be leveraged to alter cell identity. Under naive conditions, *Nanog*-KO cells self-renew; however, when switched to serum–LIF ES culture medium, NANOG helps to sustain self-renewal. We therefore transitioned *Nanog*-KO cells expressing either *Nanog*-let-7^WT^-MS2 or *Nanog*-let-7^Mut^-MS2 from naive to standard culture conditions. Cells expressing Nanog-MS2-let-7^WT^ had substantially lower self-renewal capacity than *Nanog*-let-7^Mut^-MS2-expressing cells, as evidenced by increased colony number and size in clonal assays (Fig. 6g–i). These data highlight the crucial role of miRNA-mediated RNA sequestration in regulating cell potency.

### Manipulating P-body-mediated RNA sequestration to direct cell fate

We next sought to manipulate P-bodies to facilitate otherwise inefficient cell fate conversions from human pluripotent stem cells to rare, clinically relevant cell types. We first focused on human totipotent-like cells, which have been identified in vitro at a frequency of 0.1% in naive pluripotent cell culture^58^. P-bodies in naive human pluripotent stem cells sequester transcripts associated with the totipotent 8C state (Figs. 2l and 6j), likely preventing their translation and restricting naive ES cell plasticity. To test whether P-body dissolution could overcome this restriction, we generated human naive ES cells carrying a totipotency-specific reporter, *TPRX1*-GFP^59^ (Fig. 6k). DDX6 suppression increased the proportion of *TPRX1*-GFP^+^ cells by 100-fold (~0.1% to ~10%) over previous reports^58^ (Fig. 6k,l). As further validation, we used CRISPRi to deplete *DDX6* (ref. ^28^), which activated *H3Y1*, a DUX4 target gene expressed in the 8C embryo^92^(Fig. 6m). Proliferation rates were unchanged between *DDX6*-KD and control samples (Extended Data Fig. 9k), suggesting that disrupting P-bodies increased the rate of cell fate conversion rather than cell expansion. Sorted *TPRX1*^+^ cells displayed increased expression of genes (for example, *DUXA*, *ZSCAN5B*, *ZSCAN4*) and transposable elements (for example, LTR12, MLT2A1) (Fig. 6n,o) associated with totipotency and the 8C state, while downregulating primed pluripotency markers such as *POU5F1* and MER51B^93^, consistent with enhanced cell fate conversion. These findings demonstrate that P-bodies sequester totipotency-associated transcripts to suppress their expression and suggest that manipulating P-bodies could enable the induction and expansion of rare human cell types in vitro.

We next explored whether modulating P-bodies could enhance the conversion from human ES cells to PGCLCs, which are difficult to derive in vitro, share transcriptional features with totipotent stem cells^94,95^ and are highly relevant for reproductive medicine. Our data indicated that P-bodies in primed human ES cells sequester transcripts associated with a primordial germ cell signature^96^ (Fig. 6p), including *FXR1*, *ING2*, *EIF4G3* and *MEIOC* (Extended Data Fig. 9l). We introduced shRNAs targeting *DDX6* into primed human pluripotent cells carrying primordial germ cell (PGC)-specific reporters, *TFAP2C*-GFP and *PRDM1* (*BLIMP1*)-Tomato^96^ (Fig. 6q). We induced germ cell fate and achieved a maximum conversion efficiency of less than 8% in standard conditions; however, *DDX6* suppression consistently increased the formation of *TFAP2C*-and-*BLIMP1* double-positive PGCLCs to greater than 24% (Fig. 6r). Furthermore, *DDX6*-depleted *TFAP2C*^+^*BLIMP1*^+^ PGCLCs exhibited elevated expression of PGC markers, including *TFAP2C*, *PRDM1* and *DPPA3* (Fig. 6s), without significant changes in cell proliferation (Extended Data Fig. 9m). These findings demonstrate that P-body dissolution facilitates the efficient programming of primed human ES cells toward the germ cell lineage. Our work further establishes a framework for using condensate biology to expand clinically relevant stem cell populations, highlighting P-body modulation as a strategy for cell fate engineering.

## Discussion

We identified RNA sequestration through P-bodies as a fundamental regulatory mechanism in development and differentiation (Extended Data Fig. 9n). Using FAPS, we compared P-body contents between cell types at distinct developmental stages. Our analyses revealed cell type-specific RNA sequestration patterns that reflected transcriptional profiles characteristic of preceding developmental stages. These data suggest that transcripts important for stem and progenitor cells are directed to P-bodies to inhibit their translation upon differentiation. Functionally, disrupting P-bodies in naive mouse ES cells led to the upregulation of established 2C reporters and drove a transcriptional signature characteristic of the 2C state. We observed increased ribosome occupancy and protein levels for P-body-enriched transcripts, including 2C genes, after acute disruption of P-bodies in mouse naive ES cells, suggesting that sequestration directly inhibited translation. Together, these data provide functional evidence that P-body-based regulation is sufficient to alter cell fate.

Our findings suggest the potential of manipulating P-body-mediated regulation to facilitate conversion of pluripotent stem cells into clinically relevant cell types. While human totipotent stem cells were previously identified at a low frequency (~0.1%) in naive pluripotent cell populations^58^ or generated using chemical reprogramming at low efficiency (~1%)^59^, disrupting P-body-mediated regulation strongly enhanced the frequency of *TPRX1*-GFP^+^ totipotent-like cells to nearly 10% under naive culture conditions. Totipotent cells hold promise for producing high-quality blastoids and for generating differentiated cells with unprecedented efficiency^59,97,98^. We also manipulated RNA condensates to promote specification of human pluripotent stem cells into PGCLCs, which could assist research on germ cell specification and the development of therapeutics for infertility^95,96,99^. Moreover, our P-body-seq identified fate-instructive factors within P-bodies in adult progenitor cells of the three germ layers, suggesting that manipulating P-bodies in these cells could similarly redirect cell fate or expand cell populations that are currently challenging to propagate. These insights establish a foundation for leveraging condensate biology to engineer cell fate.

P-body-mediated RNA sequestration is likely to function in biological contexts beyond development, including stem cell maintenance and homeostasis. For example, OCT4 must be precisely regulated to support stem cell self-renewal^100^; too little OCT4 leads to precocious differentiation, while only 1.5-fold overexpression directs cells toward primitive endoderm and mesoderm^101^. P-body sequestration of key transcription factors in progenitor cells may fine-tune their expression to preserve stem cell self-renewal. Indeed, we observe signal for some transcription factors (for example, those encoded by *FGF20* and *SOX1*; Fig. 2g) in both the cytoplasm and P-bodies of stable stem cell populations.

Our findings suggest that differentiating cells may employ RNA sequestration as a reversible mechanism that enables developmental plasticity during fate transitions. Analogous to the ‘gatekeeping’ role of NANOG in balancing pluripotency and differentiation^102^, P-body localization may permit stem and progenitor cells to sample the environment and direct cell fate accordingly. Supporting this model, sequestered transcripts ultimately decline in terminally differentiated cells, suggesting that P-body localization is involved in processes requiring rapid changes in gene expression programs, such as development and stress responses.

The observation that P-bodies store distinct transcripts in diverse developmental contexts raises the question of how particular mRNAs are selected for sequestration. We show that a subset of miRNAs, which are often cell type specific, is enriched in P-bodies, and disrupting miRNA–target interaction through multiple orthogonal approaches prevented P-body enrichment of corresponding miRNA targets. That said, RNA sequestration patterns likely reflect a complex interplay of regulatory factors that intersect with miRNA activity and the presence of a given miRNA in multiple cell types does not imply that its targets will exhibit identical localization outcomes between different cell types. Additional factors, including miRNA target abundance, 3′ UTR length, the presence or absence of RBPs and combinatorial targeting of transcripts by multiple miRNAs likely contribute to cell type-specific P-body enrichment.

## Methods

### Mouse embryonic stem cell culture

Mouse ES cells used in this study were C57BL/6 × 129S4Sv/Jae F1-derived v6.5 ES cells. These cells were maintained at 37 °C in a culture medium suitable for naive mouse ES cells, which consisted of a 1:1 ratio of DMEM/F12 medium (Sigma-Aldrich) and Neurobasal Medium (Life Technologies). The culture medium was supplemented with 1× MEM Non-essential Amino Acid Solution (Sigma-Aldrich), 1 mM sodium pyruvate (Sigma-Aldrich), 2 mM l-glutamine (Sigma-Aldrich), 100 U ml^−1^ penicillin (Sigma-Aldrich), 100 mg ml^−1^ streptomycin (Sigma-Aldrich), 50 μM β-mercaptoethanol (Sigma-Aldrich), N2 and B27 supplements (referred to as N2B27 medium), two small-molecule inhibitors, PD0325901 (1 μM, Axon Medchem) and CHIR99021 (3 μM, Axon Medchem), and mLIF (10 ng ml^−1^, R&D Systems).

For the induction of mouse primed ES cells, a seeding density of 1.0 × 10^5^ mouse naive ES cells was used. These cells were plated into the wells of a 12-well plate that had been precoated with Matrigel (Corning). The culture medium for this induction consisted of N2B27 medium supplemented with 20 ng ml^−1^ activin A (PeproTech), 12 ng ml^−1^ bFGF (PeproTech) and 1% KnockOut Serum Replacement (KSR, Life Technologies).

### Human primed pluripotent stem cell culture and naive conversion

Conventional human primed ES cells (UCLA-4 and WIBR3 ‘TET-OFF *METTL3*’, both female cell lines) as well as CRISPRi GEN1C hiPSCs^28^, were cultivated on Matrigel-coated dishes in mTeSR1 medium (Stemcell Technologies) at 37 °C. These cells were propagated by using a 0.02% EDTA solution (Sigma-Aldrich) for passaging purposes. Cell passaging occurred every 4–5 d to ensure proper maintenance.

In the context of the doxycycline experiment, the TET-OFF *METTL3* cell line was cultured in mTeSR1 medium supplemented with 2 μg ml^−1^ doxycycline (Sigma-Aldrich) for 1 week.

To achieve reversion to a naive state, female UCLA-4 human ES cells that had been passaged 6 d beforehand underwent a procedure involving washing with 1× PBS (Life Technologies) and then treatment with TrypLE Express Enzyme (1×, Life Technologies) for 3 min. This enzymatic treatment enabled cell dissociation into a single-cell suspension, which was subsequently plated at a density of 30,000 cells per 9.5 cm^2^ on irradiated CF-1 MEFs (a mixture of pooled male and female cells). This process occurred in human ES cell medium supplemented with 10 μM Y-27632 (Axon Medchem). After a 2-d incubation period, the medium was changed to 5i/LAF and then replaced daily. The 5i/LAF medium composition consisted of a 50:50 mixture of DMEM/F12 (Sigma-Aldrich) and Neurobasal Medium (Life Technologies), supplemented with 1× N2 supplement (Life Technologies), 1× B27 supplement (Life Technologies), 10 ng ml^−1^ bFGF (PeproTech), 1% non-essential amino acids (Sigma-Aldrich), l-glutamine (2 mM, Sigma-Aldrich), penicillin–streptomycin (Sigma-Aldrich), 0.1 mM β-mercaptoethanol (Sigma-Aldrich), 50 μg ml^−1^ BSA (Sigma-Aldrich), 0.5 μM IM-12 (Axon Medchem), 0.5 μM SB590885 (Axon Medchem), 1 μM WH-4-023 (Axon Medchem), 10 μM Y-27632 (Axon Medchem), 20 ng ml^−1^ activin A (PeproTech), 20 ng ml^−1^ rhLIF (PeproTech), 0.5% KSR (Life Technologies) and 1 μM PD0325901 (Axon Medchem). After approximately 8–10 d, cells were detached using Accutase (Sigma-Aldrich), and subsequent centrifugation was performed in fibroblast medium composed of DMEM (Sigma-Aldrich) supplemented with 10% FBS (Corning), l-glutamine (2 mM, Sigma-Aldrich), 1% non-essential amino acids (Sigma-Aldrich), penicillin–streptomycin (Sigma-Aldrich) and 0.1 mM β-mercaptoethanol (Sigma-Aldrich). The cells were then replated after passage through a 40-μm cell strainer in 5i/LAF medium on irradiated CF-1 MEFs. The established naive human ES cell lines were cultured on irradiated CF-1 MEFs at a density of 2.5 × 10^6^ cells per 9.5 cm^2^ in 5i/LAF medium and were passaged every 6–7 d. The cells were provided with fresh medium daily. These naive human ES cells were maintained under low-oxygen conditions (5% O_2_) at 37 °C.

### Three-germ layer human ES differentiation

Human mesoderm progenitors were generated from human primed ES cells using the STEMdiff Mesoderm Differentiation Kit (Stemcell Technologies) according to the manufacturer’s instructions.

For the derivation of human endoderm progenitors from human primed ES cells, the STEMdiff Definitive Endoderm Differentiation Kit (Stemcell Technologies) was used following the manufacturer’s instructions.

To induce neural progenitors, human primed ES cells were cultivated in neural induction medium. This medium composition included a 1:1 ratio of Advanced DMEM/F12 medium and Neurobasal Medium, supplemented with 1× N2 supplement (Life Technologies), 1× B27 supplement (Life Technologies), l-glutamine (2 mM, Sigma-Aldrich), 4 μM CHIR99021 (Axon Medchem), 3 μM SB432542 (Stemcell Technologies), 5 μg ml^−1^ BSA, 10 ng ml^−1^ human LIF (PeproTech) and 0.1 μM Compound E (γ-Secretase Inhibitor XXI, PeproTech). After 9 d, the cells were passaged onto Matrigel-coated dishes using Accutase (Sigma-Aldrich). They were then maintained in neural induction medium with the omission of Compound E. For the purpose of neuronal differentiation, the neural progenitors were cultured on Matrigel-coated dishes, using DMEM/F12 medium (Sigma-Aldrich), 1× N2 supplement (Life Technologies), 1× B27 supplement (Life Technologies), 300 ng ml^−1^ cAMP (Sigma-Aldrich) and 0.2 μM vitamin C (Sigma-Aldrich).

### Endoderm progenitor maturation

hPSCs were sequentially differentiated toward anterior-most primitive streak, definitive endoderm and then mature endoderm progenitors, as previously described^105^. Briefly, the following medium compositions were used on each day of differentiation: day 1, CDM2 base medium supplemented with 100 ng ml^−1^ activin A (PeproTech), 3 μM CHIR99021 (Axon Medchem), 20 ng ml^−1^ FGF2 (PeproTech) and 50 nM PI-103 (Tocris); day 2, CDM2 base medium supplemented with 100 ng ml^−1^ activin A, 250 nM LDN-193189 and 50 nM PI-103; day 3, CDM3 base medium supplemented with 20 ng ml^−1^ FGF2, 30 ng ml^−1^ BMP4 (R&D Systems), 75 nM TTNPB (Selleck) and 1 μM A-83-01 (Reprocell); days 4–6, CDM3 base medium supplemented with 10 ng ml^−1^ activin A, 30 ng ml^−1^ BMP4 and 1 μM forskolin (Sigma-Aldrich).

The composition of CDM2 basal medium is 50% IMDM medium, 50% F12, l-glutamine (2 mM, Sigma-Aldrich), penicillin–streptomycin (Sigma-Aldrich), 1 mg ml^−1^ polyvinyl alcohol (Sigma), 1% (vol/vol) chemically defined lipid concentrate (Thermo Fisher), 450 μM 1-thioglycerol (Sigma-Aldrich), 0.7 μg ml^−1^ recombinant human insulin (Sigma-Aldrich) and 15 μg ml^−1^ human transferrin (Sigma). The composition of CDM3 basal medium is 45% IMDM, 45% F12, l-glutamine (2 mM, Sigma-Aldrich), penicillin–streptomycin (Sigma-Aldrich), 10% KSR (Thermo Fisher), 1 mg ml^−1^ polyvinyl alcohol (Sigma-Aldrich) and 1% (vol/vol) chemically defined lipid concentrate (Thermo Fisher).

### Hepatocyte differentiation

For hepatic differentiation, endoderm cells were differentiated for 4 weeks in SFD-based hepatic induction medium supplemented with ascorbic acid (50 μg ml^−1^, Sigma), monothioglycerol (4.5 × 10^−4^ M, Sigma-Aldrich), BMP4 (50 ng ml^−1^), bFGF (10 ng ml^−1^), VEGF (10 ng ml^−1^), EGF (10 ng ml^−1^), TGFα (20 ng ml^−1^, PeproTech), HGF (100 ng ml^−1^, PeproTech), dexamethasone (1 × 10^−7^ M, Sigma-Aldrich) and 1% DMSO (Sigma). SFD serum-free medium consists of 75% IMDM (Life Technologies), 25% Ham’s F12 (CellGro), 0.5× N-2 Supplement (Gibco), 0.5× B27 without retinoic acid (Gibco), 0.1% BSA (Sigma-Aldrich), 50 μg ml^−1^ ascorbic acid phosphate magnesium (Wako) and 4.5 × 10^−4^ M monothioglycerol.

### Primordial germ cell-like cell differentiation

BTAG human iPSCs were maintained on iMatrix-511 (Reprocell) in StemFit medium (Reprocell) supplemented with 100 ng ml^−1^ bFGF (PeproTech). PGCLC induction was achieved as previously described^96^. iMeLCs were induced by plating 2.0 × 10^5^ hiPSCs per well of a 12-well plate coated with human plasma fibronectin (Millipore, FC010) in GK15 medium (GMEM (Life Technologies) supplemented with 15% KSR, 0.1 mM NEAA, 2 mM l-glutamine, 1 mM sodium pyruvate and 0.1 mM 2-mercaptoethanol) containing 50 ng ml^−1^ activin A (PeproTech), 3 μM CHIR99021 and 10 μM ROCK inhibitor (Y-27632, Axon Medchem). hPGCLCs were induced by plating 4 × 10^3^ iMeLCs per well in a cell-repellent V-bottom 96-well plate (Greiner Bio-One, 651970) in GK15 medium supplemented with 1,000 U ml^−1^ hLIF (Millipore, LIF1005), 200 ng ml^−1^ BMP4 (HumanZyme), 100 ng ml^−1^ SCF (R&D Systems, 455-MC), 50 ng ml^−1^ EGF (R&D Systems, 236-EG) and 10 μM ROCK inhibitor.

### Generation of GFP-LSM14A cells

For HEK293T cells, mouse ES cells and MEFs, pEGFP-LSM14A from the Weil laboratory^29^ was assembled into pLV-EF1a-Ires-Blast (Addgene, 85133).

For human ES cells, the EGFP-LSM14A fragment from pEGFP-LSM14A was amplified using primers F (CCTACCCTCGTAAAGCCGGGCTACCGGTCGCCACCATG) and R (AACTAGAAGGCACAGTTAATGGATCCTTAGGGTCCAAAAGC) and assembled into pAAVS1-tet-iCas9-BFP2 (Addgene, 125519), replacing the iCas9-BFP2 sequence with pEGFP-LSM14A.

### Generation of DDX6 degron mouse embryonic stem cells

To introduce a sequence encoding FKBP12^F36V^-HA-2A-mCherry in place of the endogenous *Ddx6* stop codon, a first donor plasmid was created by integrating two 200-bp homology arms specific to the *Ddx6* gene into the pNQL004-SOX2-FKBPV-HA2-P2A-mCherry targeting construct (Addgene, 175552). In conjunction with this donor plasmid, a *Ddx6*-targeting sgRNA (AGGTACATACGTGCTTGTTA) was cloned into the pSpCas9 (BB)-2A-Hygro plasmid (Addgene, 127763). Both donor plasmids were transfected into GFP-LSM14A mouse ES cells using the Lipofectamine 3000 transfection method. Cells exhibiting stable mCherry expression were subsequently isolated using fluorescence-activated cell sorting to establish single-cell clones. Homozygous insertion was confirmed by genotyping PCR. For this purpose, a forward primer (GCTGGGGACAGAGATCAAACC) and a reverse primer (GTAGGGCTATGCGGCCCTAC) within the DDX6 homology arms were used, along with a reverse primer specific to the mCherry sequence (ATCTGGGCAACCCCTTCTTCC). Loss of endogenous DDX6 upon dTAG-13 treatment was confirmed through western blot analysis.

### Generation of *Ago2*-KO mouse embryonic stem cells

The *Ago2*-KO cell line was established with a paired CRISPR–Cas9 approach implemented on WT mouse ES cells, as previously described in ref. ^87^.

Briefly, v6.5 mouse ES cells underwent transfection with pX458-sgRNA_Ago2_3 and pX458-sgRNA_Ago2_4 plasmids (Addgene, 73531 and 73532). GFP-positive cells were isolated by single-cell sorting in 96-well plates. To verify the deletion, PCR using the Ago2KOΔEx1_Fw (GAAGGCGAAAAAGCCTCCCC) and Ago2KOΔEx1_Rev (GAGCTAGCTTCCCGTCGC) primers was conducted. Positive clones were expanded and verified by western blot and RT–qPCR analyses.

### Short hairpin RNA-mediated microRNA gene silencing

The pLKO-shDDX6 (TRCN0000074696) vector, designed to target the human *DDX6* gene, was sourced from the Molecular Profiling Laboratory of the MGH Cancer Center.

To target the mouse *Ddx6* gene, shERWOOD UltramiR Lentiviral shRNAs (82, ACAGCTGAACCAGTTGAAGAA; 83, CTGGGCTACTCTTGCTTTTAA) were procured from Transomic Technologies, as previously described^28^.

To accomplish *Nudt21* gene silencing, oligonucleotide pairs encoding shRNA sequences specific to *Nudt21* (GGACAACTTTCTTCAAATT) were annealed and cloned into the pSICOR-mCherry-puro vector (Addgene, 31845). The efficacy of KD was validated by RT–qPCR.

In the context of miRNA KD experiments, miR-300 and control miRNA inhibitors (miRNA Power Inhibitors, Qiagen) were directly introduced into the cell culture medium. These inhibitors were added at a final concentration of 50 nM, according to the manufacturer’s recommendations.

### Generation of *TPRX1*-GFP human ES cells

For knockin *TPRX1*-EGFP generation, sgRNA (GCTCCCGAGCTAGTTTGGCG) targeting the insertion site of the donor construct containing homologous arms flanked by an EGFP–puromycin cassette, kindly gifted by the Esteban laboratory (Guangzhou Institutes of Biomedicine and Health,Chinese Academy of Sciences)^59^, was cloned into the pDonor plasmid. Donor plasmids were electroporated into UCLA-4 human ES cells using the Neon Transfection System. Homozygous insertion was confirmed by genotyping PCR.

### Generation of microRNA reporter cell lines

Plasmid pNZ176, containing Halo-NLS-MCP, was a kind gift from T. Stasevich (Department of Biochemistry and Molecular Biology, Colorado State University). The Halo-NLS-MCP fragment was amplified by PCR from this vector in parallel with PCR amplification of the Ires BFP fragment from the TRE KRAB Cas9 Ires BFP plasmid (Addgene, 85449) using Phusion polymerase (New England Biolabs) according to the manufacturer’s recommendations. The resulting fragments were used for NEBuilder HiFi DNA Assembly (New England Biolabs). The final vector was transfected along with the PBase vector (VectorBuilder) into *Nanog*-KO^106^ mouse ES cells using the Lipofectamine 3000 transfection method. BFP-positive cells were isolated using fluorescence-activated cell sorting.

The MS2 reporter construct for *Nanog* was generated using a multistep cloning approach. First, *Nanog* was amplified by PCR from the pMXs-Nanog (Addgene, 13354) plasmid using Phusion polymerase (New England Biolabs) according to the manufacturer’s recommendations. Primers to amplify *Nanog* were CGCTGTGATCGTCACTTGGCGCCGCCATGAGTGTGGGTC and CGCTGTGATCGTCACTTGGCCCACCATGAGTGTGGGTC. The PCR product was treated with DpnI (New England Biolabs) and purified using a PCR cleanup kit (Qiagen). The resulting *Nanog* fragment was used for Gibson assembly (New England Biolabs) with pUB_smFLAG_ActB_MS2 (Addgene, 81083) digested with NotI and NheI (New England Biolabs). The resulting pNANOG-MS2 plasmid was used for Gibson assembly (New England Biolabs) with the let-7^WT^ and let-7^Mut^ sequences amplified from the RL-6xB and RL-6xBMUT vectors (a kind gift fromM.R. Fabian (McGill Centre for Translational Research in Cancer, McGill University))^90^ using the following primers: Wt_F, TGAAATATGATCTAGAGGACAGCCTATTGAACTACCTCACTCG; Mut_F, TGAAATATGATCTAGAGCACAGCCTATTGAACTACCCCTCACT; R, TTGTAGGTTAGCGGCCGCACCGAATGCG. Finally, the *Nanog*-let-7^WT^-MS2 and *Nanog*-MS2-let-7^Mut^-MS2 fragments were amplified using the primers F (ACCCTCGTAAAGGTCTAGAGGCCACCATGAGTGTGGGTC) and R (GTCCTCTAGATCATATTTCACCTGGTGGAGTCAC) and assembled into the PiggyBac vector PB-TRE-EGFP-EF1a-rtTA-Blasti digested with Nhe1 and Kpn1. The resulting PiggyBac vectors carrying Nanog-let-7WT-MS2 or Nanog-MS2-let-7Mut-MS2 cassettes were used to transfect *Nanog*-KO ES cells expressing HALO-NLS-MCP IRES BFP.

### P-body purification

P-body purification was performed as previously described. Briefly, GFP-LSM14A cells were subjected to lysis for 20 min on ice using lysis buffer (comprising 50 mM Tris at pH 7.4, 1 mM EDTA, 150 mM NaCl and 0.2% Triton X-100), supplemented with 65 U ml^−1^ of the ribonuclease inhibitor RNaseOut (Promega) and an EDTA-free protease inhibitor cocktail (Roche Diagnostics). After this step, the lysates underwent centrifugation at 200*g* for 5 min at 4 °C to remove nuclei from the mixture. Residual DNA was removed by incubation in the presence of 10 mM MgSO_4_, 1 mM CaCl_2_ and 4 U ml^−1^ RQ1 DNase (Promega) for 30 min at room temperature. After centrifugation at 10,000*g* for 7 min at 4 °C, pellets were resuspended in 40 μl lysis buffer containing 80 U RNaseOut (Promega) to generate the cytoplasmic fraction. P-bodies were then isolated from this cytoplasmic fraction using FACSAria-based sorting. After sorting, the P-body samples were pelleted by centrifugation at 10,000*g* for 7 min at 4 °C. These pellets, alongside aliquots of the corresponding presorting cytoplasmic fraction, were stored at −80 °C.

### RNA sequencing

For P-body-seq, RNA was isolated from P-body and cytoplasmic fractions using the miRNeasy Micro Kit (Qiagen). Complementary DNA (cDNA) libraries were constructed using the SMART-Seq v4 Ultra kit and the Nextera XT kit for Illumina (Takara Bio). When indicated in the text, libraries were prepared using the snapTotal-seq protocol as previously described^45^. Libraries were sequenced with paired-end 150-bp reads.

### RNA-sequencing analysis

Smart-seq data were analyzed by mapping reads using hisat2.1.0 (ref ^107^) to the mouse mm10 genome assembly, the human hg38 assembly or the chicken gal6 assembly, all retrieved from UCSC. Gene counts were annotated using featureCounts version 1.6.2 using the corresponding UCSC refGene annotation.

snapTotal-seq data were processed using the nf-core RNA-seq pipeline (version 3.9) within a Singularity container. Reads were aligned to the mouse mm10 genome assembly and the human hg38 genome assembly, both of which were retrieved from UCSC with corresponding UCSC refGene annotations. Raw counts were obtained from salmon (version 1.10.2), and the resulting dds objects from nf-core were exported to R for downstream analysis. To determine P-body-enriched and -depleted genes, lowly expressed genes (<10 reads over all samples) were filtered out. Next, DESeq2 version 1.40.2 was used to calculate differential expression between the P-body and cytoplasmic fractions, setting an FDR of *P* < 0.05 as a threshold for significance. P-body-to-cytoplasmic ratios are represented as log_2_ (FC (P-body/cytoplasm)), where positive log_2_ (FC) indicates P-body enrichment and negative log_2_ (FC) indicates P-body depletion (cytoplasm enriched).

Cell fate gene sets were generated from sequencing data from the cytoplasmic fractions. DESeq2 analysis was performed comparing progenitor cell types with the differentiated cell type in pairwise comparisons (that is, naive versus primed; primed versus endoderm progenitors, mesoderm progenitors and mature endoderm progenitors; and neural progenitors versus neurons). Cell type-specific gene sets were defined as genes with log_2_ (FC) > 0 and *P*_adj_ < 0.05. For GSEA, these gene sets were further filtered with a log_2_ (FC) threshold of >2.5 and *P*_adj_ < 0.05. GSEA were performed using the fgsea version 1.26.0 R package with nperm = 50,000. Additional GSEA was performed with publicly available data from the following sources: human neurons^108^, human naive cells^103^, mouse 2C^63^, human 8C^58^, human 8CLC^58^, ZGA^58^ and PGCLCs^96^. Gene ranks were determined with the stat parameter in the results tables generated using DESeq2. GO analysis was performed using enrichGO from the clusterProfiler package, with the universe parameter set to only genes detected in sequencing data.

Orthologs were determined using Ensembl BioMart genes version 108. To calculate GC content and transcript length distributions for P-body- and cytoplasm-enriched genes, we used the biomaRt package to access cDNA sequences from Ensembl (hsapiens_gene_ensembl). Sequences were retrieved using the ‘cdna’ attribute, with GC content calculated as the percentage of G and C nucleotides and transcript length as sequence length in nucleotides. For uniformity, only the longest isoform per gene was retained. Transposable element abundance was quantified by mapping reads to RepeatMasker^109^ (mm39 and hg38) annotations using a custom pipeline to identify repeat family expression patterns across samples.

miRNA targets were obtained from the miRDB database^110^, in which targets with a threshold score greater than the 75th percentile were deemed a target. To infer targets of RBPs, we used previously compiled CLIP data^29^ from the CLIPdb 1.0 database, focusing on targets of RBPs that are bound at the 3′ UTR of the target genes. For miRNA and RBP plots, the log_2_ (FC) P-body-to-cytoplasm ratio of the targets was plotted.

To calculate GC content and transcript length distributions for P-body- and cytoplasm-enriched genes, we used the biomaRt package to access cDNA sequences from Ensembl (hsapiens_gene_ensembl). Sequences were retrieved using the cdna attribute, with GC content calculated as the percentage of G and C nucleotides and transcript length as sequence length in nucleotides. For uniformity, only the longest isoform per gene was retained. To determine the relationship between P-body enrichment and expression levels, RPKM of genes in naive and primed human ES cells were obtained from GSE111020 (ref. ^56^).

### RNA stability analysis

To assess the correlation between P-body enrichment and mRNA half-life, publicly available SLAM-seq data from HEK293T cells^49,111^, mouse ES cells^66^ and human iPSCs were used^57^. Additionally, polyA tail length, as an indicator of mRNA stability, was evaluated by comparing PAIso–seq data from mouse ES cells^67^. For each dataset, a Pearson correlation was calculated.

Gene coverage plots were generated using PyCoverage (geneBody_coverage.py from the RSeQC package). BED12 files were created for P-body-enriched genes, cytoplasm-enriched genes and all transcripts for each cell type (gene sets defined previously in RNA-seq methods). Gene annotation files in BED format were downloaded from the UCSC Genome Browser for mm10 and hg38 genome assemblies, selecting Genes and Gene Predictions group, NCBI RefSeq track and the UCSC RefSeq (refGene) table. These files were converted to BED12 format with custom R code, with which block sizes were calculated as the difference between exon start and end positions and block starts were calculated as the difference between exon start and transcript start. Only the longest isoform per gene was retained. Filtered genome BED12 files for the specified gene lists were then used in the geneBody_coverage.py command, with BAM files for each fraction (two replicates each) analyzed per cell type and species. Resulting outputs were plotted in R, displaying read counts across gene bodies.

### Polysome profiling analysis

RNA-seq data from polysome-containing samples and total RNA samples were analyzed using the nf-core/rnaseq pipeline (version 3.9) with default settings. Reads were aligned to the mouse mm10 genome assembly, retrieved from UCSC using the corresponding UCSC refGene annotation. Raw counts were obtained from salmon version 1.10.2.

To determine differentially expressed genes in *DDX6*-KD samples, lowly expressed genes (<10 reads over all samples) were filtered out. Next, DESeq2 was used to call differential expression between control and dTAG-13-treated total RNA samples. An FDR of *P* < 0.05 was used as the threshold for significance of differentially expressed genes.

For polysome fractionation analysis, genes were filtered to protein-coding genes using the mm10 refGene annotation. Ribosome occupancy was calculated as log_2_ (ribosome-bound/total RNA) using DESeq2. Change in ribosome occupancy was calculated as ribosome occupancy degron − ribosome occupancy control. GSEA was performed as described above using mouse gene sets from 2C^63^, naive^104^ and primed^69^ cells.

### Translation efficiency analysis

Ribo-seq data were obtained from ref. ^68^. To download Ribo-seq raw data, GSE133794 was downloaded from the Gene Expression Omnibus and processed using the nf-core/rnaseq pipeline (version 3.9). Reads were aligned to the mouse mm10 genome assembly, retrieved from UCSC using the corresponding UCSC refGene annotation. Raw reads were obtained from salmon version 1.10.2. Reads were normalized by RPKM, and lowly expressed genes (<10 reads over all samples) were filtered out. Translation efficiency was calculated as log_2_ (reads in Ribo-seq/reads in RNA-seq) for each gene.

### Reporting summary

Further information on research design is available in the Nature Portfolio Reporting Summary linked to this article.

## Online content

Any methods, additional references, Nature Portfolio reporting summaries, source data, extended data, supplementary information, acknowledgements, peer review information; details of author contributions and competing interests; and statements of data and code availability are available at https://doi.org/10.1038/s41587-025-02853-z.

## Supplementary information

Supplementary Information Supplementary Methods.

Reporting Summary

Supplementary Table 1 P-body-enriched genes in mouse cell types.

Supplementary Table 2 P-body-enriched genes in human cell types.

## Source data

Source Data Fig. 5 Statistical source data.

Source Data Fig. 6 Unprocessed western blot membrane.

Source Data Extended Data Fig. 6 Unprocessed western blot membrane.

Source Data Extended Data Fig. 8 Statistical source data.

Source Data Extended Data Fig. 9 Unprocessed western blot membrane.

## Data Availability

The accession number for RNA-seq, small RNA-seq, PANDORA-seq and polysome profiling data reported in this paper is GSE245943. Mass spectrometry proteomics data have been deposited to the ProteomeXchange Consortium via the PRIDE partner repository with the dataset identifier PXD058827. Source data are provided with this paper.
