# Supplementary material for: Selective RNA sequestration in biomolecular condensates directs cell fate transitions
Source: Nat Biotechnol. 2025 Oct 28;44(9):1553–68. doi: 10.1038/s41587-025-02853-z (PMC12728835; doi:10.1038/s41587-025-02853-z)
Supplement: Supplementary file 1 [file 41587_2025_2853_MOESM1_ESM.pdf]

nature biotechnology

Supplementary information https://doi.org/10.1038/s41587-025-02853-z

# Selective RNA sequestration in biomolecular condensates directs cell fate transitions

In the format provided by the authors and unedited

# SUPPLEMENTARY INFORMATION

## SUPPLEMENTARY METHODS

### HEK293T culture

HEK293T cells (human, female) were obtained from ATCC (Cat# CRL3216). These cells were maintained at a temperature of 37°C and were cultured in DMEM medium (Life Technologies), supplemented with 10% FBS (Corning), L-Glutamine (2 mM, Sigma-Aldrich), Penicillin (100 U/mL, Sigma-Aldrich), and Streptomycin (100 mg/mL, Sigma-Aldrich).

### Derivation of mouse embryonic fibroblasts

Mouse embryonic fibroblasts (MEFs) were generated following a protocol similar to that described by[112]. Briefly, embryos were collected from timed mating at embryonic day 13.5 (E13.5). Subsequently, the head, limbs, spinal cord, gonads, and internal organs were carefully excised, leaving behind the remaining tissue. This tissue was then finely minced and treated with 200 μL of Trypsin-EDTA (Sigma-Aldrich). After a 5-minute incubation at 37°C, the enzymatic reaction was halted by adding 10 mL of MEF growth media composed of DMEM (Sigma-Aldrich), supplemented with 10% fetal bovine serum (Corning), L-Glutamine (2 mM, Sigma-Aldrich), and penicillin-streptomycin (Sigma-Aldrich). The cells were cultured at 37°C under low oxygen conditions (5% O2).

For reprogramming experiments, MEFs were isolated from mice harboring an inducible, polycistronic OKSM cassette located in the 3' untranslated region (UTR) of the

*Col1a1* gene. Additionally, these MEFs carried the M2-rtTA transactivator at the Rosa26 locus, along with an EGFP reporter construct regulated by *Pou5f1* regulatory elements[113]. All procedures involving mice were conducted in accordance with the guidelines stipulated by the Institutional Animal Care and Use Committee (IACUC) of Baylor College of Medicine and were in compliance with the approved protocol no. AN-8464.

**Induction of pluripotency**

To initiate the expression of pluripotency factors, reprogrammable MEFs were cultivated in induction media containing KO-DMEM (Life Technologies), with 15% fetal bovine serum (Corning), 1X non-essential amino acids (Life Technologies), L-Glutamine (2 mM, Sigma-Aldrich), 1000 U/ml LIF (10 ng/mL, R&D Systems), 55 mM beta-mercaptoethanol (Sigma-Aldrich), and supplemented with 50 µg/ml ascorbic acid (Sigma Aldrich) and 2 µg/ml doxycycline hyclate (Sigma-Aldrich).

For P-body isolation, reprogrammable MEFs were subjected to induction for a duration of 3 days, at which time, samples were harvested. Alternatively, to achieve complete reprogramming, these MEFs were maintained under induction conditions for a period of 8 days.

**Lentivirus production and transduction**

HEK293T cells were co-transfected with a transfer plasmid along with packaging plasmids VSV-G and D8.9, using a calcium phosphate transfection method. Subsequently, viral supernatants were harvested within a time frame of 24 to 32 hours following transfection. These harvested viral supernatants underwent concentration

through ultracentrifugation at a speed of 21,000×g for a duration of 2 hours, all performed at a temperature of 4°C. Once concentrated, the viruses were resuspended in Opti-MEM (Gibco). If not intended for immediate use, the concentrated viruses were stored at a temperature of -80°C.

**Western blot**

Cells were lysed in RIPA buffer (50 mM Tris-HCl (pH 8.0), 150 mM NaCl, 0.1% SDS, 0.5% sodium deoxycholate, 1% Triton-X-100, 1 mM EDTA, protease inhibitors, and benzonase). Lysates were subjected to standard western blotting using the following antibodies: DDX6 (1:5000, Cat#NB200-192, Novus Biologicals), Argonaute-2 (AGO2) (1:1000, Cat#C34C6; Cell Signaling Technologies), HRP-rabbit anti-human/mouse β-Actin (1:3000, Cat#5125, Cell Signaling), Goat, anti-rabbit-HRP-conjugated (Cat#31460, Thermo Fisher Scientific, 1:2,000 dilution), Rabbit, anti-mouse-HRP-conjugated (Cat#31450, Thermo Fisher Scientific, 1:2,000 dilution).

**Immunocytochemistry and confocal microscopy**

Cells were fixed using 4% methanol-free formaldehyde for a duration of 15 minutes at room temperature. After fixation, the cells were permeabilized and simultaneously blocked using a solution of 0.1% Triton-X and 10% donkey serum. Subsequently, primary antibodies were applied to the cells and allowed to incubate at 4°C overnight. Following this step, the cells were stained with Alexa Fluor-conjugated secondary antibodies, at room temperature for a duration of one hour. Where mentioned, Phalloidin-iFluor 594 Reagent (1:500 dilution) (Abcam, Cat# ab176757) was combined with the secondary

antibody to facilitate the labeling of membranes. Nuclei were counterstained using DAPI. The slides were mounted using ProLong Diamond (Life Technologies) and subsequently allowed to cure at room temperature. The following primary antibodies were used in this study: EDC4 (1:50, Cat#ab72408, Abcam), GFP (1:400, Cat#GFP-1020, Aves Lab), KLF17 (1:100, HPA024629, SIGMA), NANOG (D73G4) (Cat#4903, 1:300, Cell Signaling), BRACHYURY (T) (1:50, Cat#AF2085, R&D Systems), FOXA2 (1:400, Cat#8186, Cell Signaling), SOX1 (1:200, Cat#AF3369, R&D Systems), βIII-TUBULIN (TUJ1) (1:200, Cat# 801213, Biolegend), METTL3 (1:100, Cat# 15073-1-AP, Proteintech). Imaging was carried out using confocal microscopy equipped with either a 40x or 63x objective lens on an LSM 900 with Airyscan 2 (Zeiss). The acquired images with ZEN Blue 3.1 (Zeiss) were then subjected to analysis using FIJI (ImageJ 2.14.0/1.54f) software. P-bodies were counted manually to calculate number of RNP particles per cell.

**Immunofluorescence and Fluorescence in situ hybridization (IF-FISH)**

Cells were fixed using 4% methanol-free formaldehyde for a period of 30 minutes on ice. Subsequently, cells underwent permeabilization through a 0.1% Triton-X100 solution for 30 minutes at room temperature. For antibody labeling, cells were immunolabeled with chicken anti-GFP (1:400, Cat#GFP-1020, Aves Lab) at 37°C for 1 hour in 2xSSC buffer, followed with Alexa Fluor 488-conjugated donkey anti-chicken IgG (H+L) (1:500, Cat# A78948, Life Technologies) for a duration of 30 minutes at room temperature. Afterward, a post-fixation step was carried out using 4% methanol-free formaldehyde for 15 minutes at room temperature. Following this, the cells were labeled using a hybridization buffer

containing POLK (SMF-1063-5 Stellaris, LGC Biosearch Technologies) or control probes (T30-QUASAR 570-1 Stellaris) at 37°C overnight.

After washes, nuclei were counterstained using DAPI, and samples were imaged within the next three days. The imaging was conducted using a Cytivia DVLive system with an Olympus PlanApo N 60x/1.42NA oil objective, capturing 6 fields per well and collecting a total z-stack of approximately 15µm with optical spacing of 0.25µm. Images were then deconvolved using a conservative restorative algorithm, and then 16-bit TIFF files were used for image quantification. Quantification was performed using FISH-quant[114].

**Flow cytometry**

For intracellular staining of Ki-67, cells were fixed and permeabilized using Transcription Factor Buffer Set (BD Biosciences), according to manufacturer's instructions, followed by staining with Alexa 647-anti-mouse/human Ki-67 (1:300 dilution) (11F6) (Cat# 151206, BioLegend). Samples were acquired through FACSDiva v.8.0.1 (BD Biosciences) on an LSR-II. All flow cytometry data were analyzed on FlowJo v.10.8.2 (BD Biosciences)

**RNA dot blot**

200ng of RNA was loaded on a positively charged nylon transfer membrane (GE Healthcare). Membrane was UV cross-linked at 1200 J/m2, and subsequently blocked with gentle agitation for 1 hr at room temperature. Membrane was incubated with Anti-N6-Methyladenosine (m6A) antibody (1:3000 dilution) (Cat# SAB5600251, Sigma) with gentle agitation overnight at 4°C. Membrane was then incubate with HRP-conjugated

secondary antibody (Anti-rabbit IgG, HRP-linked Antibody) at 1:3000 with gentle agitation for 1 hr at room temperature. Methylene blue staining was used to normalized sample loading.

**Colony number quantification**

ImageJ was used to quantify colonies based on alkaline phosphatase (AP) images as previously described[112]. A region of interest of identical size was selected in each well using the oval tool. The "find edges" command was executed and the "color threshold tool" was used with the following parameters: "default thresholding method", "red" as the threshold color, "HSB" as the color space, and "dark background." "Analyze particles" was used to quantify the number of AP-positive colonies and the area of those colonies using the following parameters: "Size (pixel^2)=9-infinity"; "Circularity=0–1"; "Exclude on edges" was applied; "Include holes" was applied. The settings were identical for all samples processed.

**Quantitative RT-PCR (qRT-PCR)**

RNA extraction from cells was performed using the Monarch Total RNA Miniprep Kit (New England Biolabs). After RNA extraction, reverse transcription to cDNA was performed utilizing the LunaScript RT SuperMix Kit (New England Biolabs), according to the manufacturer's instructions. For qRT-PCR analysis, a master mix was prepared comprising Luna Universal qPCR Master Mix (New England Biolabs) along with pre-designed primers (Sigma-Aldrich) at a concentration of 0.5μM for the target genes. The qPCR reactions were conducted on a CFX96 Real-Time PCR Detection System (Bio-

Rad). The cycling conditions for the qRT-PCR reactions were as follows: an initial denaturation step at 95°C for 1 minute, followed by 40 cycles of denaturation at 95°C for 15 seconds, and annealing/extension at 60°C for 30 seconds. Primers are available upon request.

**Small RNA-seq**

Purified RNA from P-body and unsorted fractions of naïve and primed mouse ES cells, primed human ES cells, and human neural progenitors was mixed with RNA loading dye (New England Biolabs; B0363S) by equal volume, and incubated at 75 °C for 5 min. The mixture was loaded into 15% (wt/vol) urea polyacrylamide gel and ran in a 1× TBE running buffer at 200 V until the bromophenol blue reached the bottom of the gel. After staining with SYBR Gold solution (Invitrogen; S11494), the gel that contained 15-50 nucleotides RNAs was excised based on small RNA ladders (New England Biolabs) and Takara (3416) and eluted in 0.3 M sodium acetate (Invitrogen) and 100 U $ml^{-1}$ RNase inhibitor (New England Biolabs) overnight at 4 °C. The sample was then centrifuged for 10 min at 12,000g (4 °C). The aqueous phase was mixed with pure ethanol, 3 M sodium acetate and linear acrylamide (Invitrogen) at a ratio of 3:9:0.3:0.01. Then, the sample was incubated at -20 °C for 2 hours and centrifuged for 25 minutes at 12,000g (4 °C). After removing the supernatant, the precipitation was resuspended in nuclease-free water, quantified, and stored at -80°C or used for further processing. The size-isolated RNAs were incubated in 50µL reaction mixture containing 5 µL 10× PNK buffer (New England Biolabs), 1 mM ATP (New England Biolabs), 10 U T4PNK (New England Biolabs) and RNA at 37 °C for 20 min. Then, the mixture was added to 500 µL TRIzol reagent to perform

the RNA isolation procedure. After purification, the RNAs were incubated in 50 µl reaction mixture containing 50 mM HEPES (pH 8.0), 75 µM ferrous ammonium sulfate (pH 5.0), 1 mM α-ketoglutaric acid, 2 mM sodium ascorbate, 50 mg $l^{-1}$ bovine serum albumin (Sigma-Aldrich; A7906-500G), 2,000 U $ml^{-1}$ RNase inhibitor, and equal concentrations of AlkB and RNA at 37°C for 30 min. Then, the mixture was added to 500µL TRIzol reagent to perform the RNA isolation. Small RNA libraries were constructed using the NEBNext Small RNA Library Prep Set for Illumina (New England Biolabs). Libraries were amplified and sequenced using the SE100 strategy on the Illumina system by the University of California, San Diego IGM Genomics Center.

**Polysome profiling**

Sucrose gradients were made by preparing 10% and 50% (w/w) sucrose solutions in polysome buffer (15 mM Tris-HCl, 15 mM MgCl2, 0.3M NaCl, ph 7.4) supplemented with 100ug/mL Cycloheximide **(**CHX, Tocris). 5.9mL 50% sucrose was loaded into each centrifuge tube (Polyallomer 14x95mm, Beckman #331372), followed by 10% sucrose on top, then placed in a gradient maker (Gradient Master, Biocomp model 108), with the program set to: SHORT SUCR 10–50% WW 1st (Time 1:55; Angle 81.5, Speed 25 rpm; 1 cycle). Prepared gradients were stored at 4°C for 2 hours prior to use.

DDX6 degron 2i mouse ES cells (2x 10cm plates for each biological replicate) were grown until confluent. To induce degron, dTag13 (dTAG13 Sigma) was added to a final concentration of 1µM. After 6 hours, cells were treated with 100 µg/ml CHX for 10 minutes at 37°C. Cells were washed once with 1X PBS supplemented with 100 µg/mL CHX, and harvested using Accutase supplemented with 100 µg/mL CHX. After quenching, cells

were spun down at 200g for 5 min, and washed two times with 10 mL 1X PBS supplemented with 100 μg/mL CHX. Cell pellets were resuspended in 400 μL lysis buffer (polysome buffer with 1% Triton-x 100, 0.1 U/μL RNase OUT (Invitrogen), 0.125 U/μL DNase I (Zymo Research), incubated on ice for 10 minutes, and then centrifuged at 12,000xg at 4°C for 10 min. 50 μL of each lysate was kept for total RNA samples, and the remaining 350 μL were used for fractionation. Control cells were harvested in the same manner alongside dTag13 treated cells.

For fractionation, 350 μL of the lysate was loaded onto each gradient and centrifuged at 30,000 RPM for 2hrs at 4°C (Beckman Optima-XE 90, SW41 rotor). 300 μL fractions were collected from the top using a fractionator with a UV monitor (Density Gradient Fractionation System—Teledyne ISCO). Ribosome-containing fractions were identified by the UV spectrum, and pooled to make up the ribosome-bound pool. RNA from all samples was extracted using the Direct-zol RNA Miniprep kit (Zymo Research, R2050). Prior to loading on the columns, Trizol-LS (Invitrogen) was added to sucrose at 1:1 volume, followed by 1:1 100% ethanol. RNA integrity and concentration were checked using Agilent tape station, and libraries were prepped from a starting input of 500ng using the NEB Ultra II Directional RNA Library Prep Kit for Illumina (NEB). Libraries were quantified using the NEBNext Library Quant Kit for Illumina (NEB) and pooled to equal amounts of each library. Final libraries were sent to Novogene for sequencing and were sequenced at a depth of >35M reads.

**Proteomics analysis**

Cell pellets were resuspended in lysis buffer (8 M urea, 150 mM NaCl, 100 mM Tris pH 8) and sonicated for 20s at 20% amplitude. Samples were incubated with dithiothreitol (DTT) to a concentration of 5 mM for 30 minutes at room temperature. Iodoacetamide (IAA) was added to a final concentration of 10 mM, and samples were incubated for 30 minutes in the dark. A final incubation with 10 mM DTT was performed for 30 minutes at room temperature. An automated SP3[115] digestion protocol was performed on the King Fisher Flex (Thermo). Briefly, 20 uL of MagReSyn Amine beads per sample were washed twice with 60 uL of HPLC water. After washing, beads were resuspended in 20 uL HPLC water. Protein lysates were added to a deep well plate, where they were diluted to 100 uL with water. 20 uL of beads were added to each well, and each sample was diluted with 100% acetonitrile (ACN) to a final ACN concentration of 70% to induce aggregation of proteins to the beads. The beads were then washed thrice with 150uL of 95% ACN, followed by two washes with 150uL of 70% ethanol. Finally, beads were deposited in a plate containing trypsin (Promega, 1:100 enzyme:protein ratio) and lysC (Wako, (1:100 enzyme:protein ratio) in 50 mM ammonium bicarbonate. Beads were incubated overnight at 37°C with 800 rpm mixing and the resulting supernatant, containing the digested peptides, was transferred to a PCR filter plate to remove residual beads. After filtration, protease activity was quenched by acidification with formic acid to a final volume percentage of 1%. This first elution was then dried by vacuum centrifugation. The elution of peptides off the beads was repeated by the addition 50 uL of water to the beads with shaking for 1h at RT. Following shaking, the liquid was collected from the beads, filtered, and combined with the first elution, and again dried by vacuum centrifugation.

The resulting peptides were separated on a PepSep reverse-phase C18 column (1.9 µm particles, 15 cm, 150 mm ID) (Bruker) with a gradient of 3–28% buffer B (0.1% formic acid in acetonitrile) over buffer A (0.1% formic acid in water) over 67 minutes, an increase to 40% B in 5 minutes, and held at 95% B for 8 minutes. Eluting peptide cations were analyzed by electrospray ionization on an Orbitrap Eclipse (Thermo Fisher Scientific). For data-independent acquisition (DIA) analysis, MS1 scans of peptide precursors were performed at 120,000 resolution (200 m/z) over a scan range of 350-1050 m/z, with an AGC target of 250% and a max injection time of 100 ms. MS2 scans were collected over 350-950 m/z in 15 m/z isolation windows with a 0.5 m/z overlap. Maximum injection time was set to auto, with AGC set to standard, and an MS2 resolution of 15,000. Higher energy collisional dissociation (HCD) was performed at a NCE of 28%. Six gas-phase fractionation (GPF)[116] -DIA runs were collected from a pooled sample of all conditions. tSIM MS1 scans were performed at 120,000. tMS2 scans were collected at 30,000 fragment resolution, an AGC target of 1e6, a maximum ion injection time of 60 ms, and a NCE of 26. Data was collected using 4*m*/*z* precursor isolation windows in a staggered-window pattern, with each GPF fraction covering an approximately 100 m/z range.

All data was searched against the Uniprot mouse database (downloaded 06/11/24) using the GPF library in Spectronaut (Biognosys, version 19.0). Default settings, including trypsin digestion, variable modifications of methionine oxidation and N-termini acetylation, and fixed modification of cysteine carbamidomethylation, were used. DIA runs were filtered to obtain a false discovery rate of 1% at the peptide spectrum match and protein level[117]. Quantitative analysis was performed in the R statistical programming language

using the artMS package (release 3.20) and MSstats (version 3.20)[118]. For MSstats analysis, normalization_method was set to equalizeMedians, and censored missing values were imputed by the Accelerated Failure Time model.

**Motif Enrichment Analysis**

To identify sequence motifs enriched in P-body transcripts, we used the STREME[119] function from the MEME suite[120] (version 5.5.7). For target sequences, FASTA files were generated containing the longest 3' UTR of the top 100 P-body-enriched genes for each cell type, while control sequences consisted of FASTA files with the longest 3' UTR of the top 100 P-body-depleted genes. To create these FASTA files, BED files were downloaded from the UCSC table browser for mm10 and hg38 annotations by selecting the Genes and Gene Predictions group, NCBI RefSeq track, and UCSC RefSeq (refGene) table. For extraction of 3' UTR sequences only, the setting "create one BED record per 3' UTR Exons" was applied, and the longest annotated 3' UTR per gene was retained. These BED files were filtered to include only the top 100 P-body-enriched and top 100 P-body-depleted genes and then converted to FASTA format using the bedtools (v2.28.0) getfastafunction. Tomtom[120] (MEME Suite) was subsequently run using the streme.txt output from STREME and the following target motif databases: RNA (DNA-encoded) Ray 2013 RNA Binding Protein dataset and miRBase[121] (v22) Single Species miRNA (DNA-encoded) for the respective species.

**Small RNA-seq analysis**

The raw reads were trimmed and annotated by performing SPORTS1.1.2 (parameters: sports.pl-M 1 -a -x GUUCAGAGUUCUACAGUCCGACGAUC -y AAGATCGGAAGAGCACACGTCT -l 15-L 45 -s) with the pre-compiled mouse annotation database (mm39 (for mouse), hg38 (for human)). Pairwise comparison of differentially expressed sncRNAs among different groups was performed using the R package DEseq2 with a threshold of $FC > 2$ and adjusted $p<0.05$.

**Alternative Polyadenylation analysis**

Alternative polyadenylation (APA) from P-body and cytoplasmic fractions of control MEFs, d3 reprogrammed MEFs, and d3 reprogrammed MEFs with Nudt21 KD were analyzed using LABRAT (v1.0)[122]. Paired-end FASTQ files were input to LABRAT's runSalmon mode to quantify transcripts, using the reference genome FASTA (mm10) and GFF annotation (gencode vM17) files with the lasttwoexons option specified. The calculatepsi mode was subsequently run on the Salmon output to calculate the Percentage of Distal polyA site Usage Index (PDUI), which represents the reads from the distal polyA site/ total reads from both isoforms. The mean PDUI for each gene was calculated in each fraction (P-body and cytoplasm). Before plotting, genes not annotated as protein coding in EnsDb.Mmusculus.v79, and APA events annotated as ALE or mixed isoforms were excluded.

To identify polyadenylation site (PAS) usage in d3 reprogrammed MEFs with or without Nudt21 KD, we used PAS-seq data from GSE99922[5]. Read counts were normalized using TMM-corrected values via edgeR. For each gene, the two most highly expressed PASs were identified and labeled as proximal or distal based on strand-aware

genomic position. APA shifts were calculated as log10((proximal usage in control / distal usage in control) / (proximal usage in Nudt21 KD / distal usage in Nudt21 KD)). Negative values indicate increased proximal PAS usage. Genes were considered to have significant APA shifts between control and Nudt21 KD MEFs if either PAS had FDR < 0.05 and the absolute log10 shift exceeded log10(1.5).

**Statistical Analysis**

Quantitative data are presented as mean ± s.d. Statistical analyses were performed using Prism 9.3.0 software (GraphPad). Details for statistical analyses, including replicate numbers, are provided in the figure legends.

Bioinformatics analyses and statistics were done in R 4.3.1. Unless otherwise specified, plots were made using ggplot2 version 3.5.1. Boxplots show mean plus quartiles, and whiskers show minimum and maximum quartiles excluding outliers. Comparisons between groups were performed using Wilcoxon rank-sum or signed-rank tests (for unpaired or paired data, respectively) and two-sample *t*-tests as appropriate, using the rstatix R package. Wilcoxon effect sizes (*r*) were calculated by converting *p*-values to *Z*-scores and dividing by the square root of the sample size, with the direction based on whether the median difference was above or below zero. Where applicable, Cliff's delta was used to quantify effect size using the cliff.delta() function.

## REFERENCES

112. Bar-Nur, O. *et al.* Small molecules facilitate rapid and synchronous iPSC generation. *Nat Methods* **11**, 1170-1176 (2014).

113. Lengner, C.J. *et al.* Oct4 expression is not required for mouse somatic stem cell self-renewal. *Cell stem cell* **1**, 403-415 (2007).

114. Imbert, A. *et al.* FISH-quant v2: a scalable and modular tool for smFISH image analysis. *RNA* **28**, 786-795 (2022).

115. Hughes, C.S. *et al.* Single-pot, solid-phase-enhanced sample preparation for proteomics experiments. *Nat Protoc* **14**, 68-85 (2019).

116. Searle, B.C. *et al.* Generating high quality libraries for DIA MS with empirically corrected peptide predictions. *Nat Commun* **11**, 1548 (2020).

117. Elias, J.E. & Gygi, S.P. Target-decoy search strategy for increased confidence in large-scale protein identifications by mass spectrometry. *Nat Methods* **4**, 207-214 (2007).

118. Choi, M. *et al.* MSstats: an R package for statistical analysis of quantitative mass spectrometry-based proteomic experiments. *Bioinformatics* **30**, 2524-2526 (2014).

119. Bailey, T.L. STREME: accurate and versatile sequence motif discovery. *Bioinformatics* **37**, 2834-2840 (2021).

120. Bailey, T.L., Johnson, J., Grant, C.E. & Noble, W.S. The MEME Suite. *Nucleic Acids Res* **43**, W39-49 (2015).

121. Kozomara, A., Birgaoanu, M. & Griffiths-Jones, S. miRBase: from microRNA sequences to function. *Nucleic Acids Res* **47**, D155-D162 (2019).

122. Gillen, A.E., Goering, R. & Taliaferro, J.M. Quantifying alternative polyadenylation in RNAseq data with LABRAT. *Methods Enzymol* **655**, 245-263 (2021).
