# Supplementary material for: Selective RNA sequestration in biomolecular condensates directs cell fate transitions
Source: Nat Biotechnol. 2025 Oct 28;44(9):1553–68. doi: 10.1038/s41587-025-02853-z (PMC12728835; doi:10.1038/s41587-025-02853-z)
Supplement: Supplementary file 2 [file 41587_2025_2853_MOESM2_ESM.pdf]

nature portfolio

Corresponding author(s): Bruno Di Stefano

Last updated by author(s): Sep 5, 2025

Reporting Summary

Nature Portfolio wishes to improve the reproducibility of the work that we publish. This form provides structure for consistency and transparency in reporting. For further information on Nature Portfolio policies, see our Editorial Policies and the Editorial Policy Checklist.

Statistics

For all statistical analyses, confirm that the following items are present in the figure legend, table legend, main text, or Methods section.

n/a	Confirmed	
☐	☒	The exact sample size (*n*) for each experimental group/condition, given as a discrete number and unit of measurement
☐	☒	A statement on whether measurements were taken from distinct samples or whether the same sample was measured repeatedly
☐	☒	The statistical test(s) used AND whether they are one- or two-sided<br>*Only common tests should be described solely by name; describe more complex techniques in the Methods section.*
☐	☒	A description of all covariates tested
☐	☒	A description of any assumptions or corrections, such as tests of normality and adjustment for multiple comparisons
☐	☒	A full description of the statistical parameters including central tendency (e.g. means) or other basic estimates (e.g. regression coefficient) AND variation (e.g. standard deviation) or associated estimates of uncertainty (e.g. confidence intervals)
☐	☒	For null hypothesis testing, the test statistic (e.g. *F*, *t*, *r*) with confidence intervals, effect sizes, degrees of freedom and *P* value noted<br>*Give P values as exact values whenever suitable.*
☒	☐	For Bayesian analysis, information on the choice of priors and Markov chain Monte Carlo settings
☒	☐	For hierarchical and complex designs, identification of the appropriate level for tests and full reporting of outcomes
☐	☒	Estimates of effect sizes (e.g. Cohen's *d*, Pearson's *r*), indicating how they were calculated

Our web collection on statistics for biologists contains articles on many of the points above.

Software and code

Policy information about availability of computer code

Data collection	Software used: FACSDiva v.8.0.1 (BD Biosciences), ZEN Blue 3.1 (Zeiss)
Data analysis	GraphPad Prism v9.3.0<br>FIJI (ImageJ 2.14.0/1.54f)<br>FlowJo v10.8.2 (BD Biosciences)<br>HISAT2 v2.1.0<br>Salmon v1.10.2<br>nf-core RNA-seq pipeline v3.9<br>R v4.3.1 with packages: DESeq2 v1.40.2, fgsea v1.26.0, ggVennDiagram v1.2.0, clusterProfiler v4.8.3 (including enrichGO), eulerr v7.0.2, biomaRt v2.58.0, MSstats v3.20, artMS v3.20, edgeR v3.42.4<br>Spectronaut v19.0 (Biognosys)<br>Ensembl BioMart genes v108<br>miRDB database<br>POSTAR3/CLIPdb database<br>RepeatMasker (version?)<br>LABRAT v1.0<br>PyCoverage (version?)<br>MEME Suite v5.5.7 (including STREME and Tomtom)

For manuscripts utilizing custom algorithms or software that are central to the research but not yet described in published literature, software must be made available to editors and reviewers. We strongly encourage code deposition in a community repository (e.g. GitHub). See the Nature Portfolio guidelines for submitting code & software for further information.

Data

Policy information about availability of data

All manuscripts must include a data availability statement. This statement should provide the following information, where applicable:

- Accession codes, unique identifiers, or web links for publicly available datasets
- A description of any restrictions on data availability
- For clinical datasets or third party data, please ensure that the statement adheres to our policy

The accession numbers for the RNA-seq, small RNA-seq, PANDORA-seq, and polysome profiling data reported in this paper are GSE245943. The mass spectrometry proteomics data have been deposited to the ProteomeXchange Consortium via the PRIDE partner repository with the dataset identifier PXD058827.

Research involving human participants, their data, or biological material

Policy information about studies with human participants or human data. See also policy information about sex, gender (identity/presentation), and sexual orientation and race, ethnicity and racism.

Reporting on sex and gender	n/a
Reporting on race, ethnicity, or other socially relevant groupings	n/a
Population characteristics	n/a
Recruitment	n/a
Ethics oversight	n/a

Note that full information on the approval of the study protocol must also be provided in the manuscript.

Field-specific reporting

Please select the one below that is the best fit for your research. If you are not sure, read the appropriate sections before making your selection.

☒ Life sciences ☐ Behavioural & social sciences ☐ Ecological, evolutionary & environmental sciences

For a reference copy of the document with all sections, see nature.com/documents/nr-reporting-summary-flat.pdf

Life sciences study design

All studies must disclose on these points even when the disclosure is negative.

Sample size	For all experiments, sample size was determined based on preliminary experiments or similar experiments in previously published literature (e.g. Kodali et al, Nat Cell Biol. 2024 Oct;26(10):1745-1758; Di Stefano et al Nat Methods. 2018 Sep;15(9):732-740; Hubstenberger et al Mol Cell. 2017 Oct 5;68(1):144-157.e5).
Data exclusions	No data were excluded from this study.
Replication	All attempts to independently reproduce experiments were successful (typically reproduced at least 3 times, with multiple biological replicates each time), except for large-scale sequencing assays (e.g., RNA-seq, proteomics, polysome profiling), which were cost-prohibitive to repeat independently, but were performed using at least two biological replicates.
Randomization	Randomization was irrelevant and not performed for in vitro experiments with cultured cells. Mice were only utilized to derive primary fibroblast cultures.
Blinding	Investigators were blinded for quantitative counting analyses (e.g. P-body counting, smFISH analysis, mESC colony counting), with data collection and analysis performed by researchers who were unaware of sample identity. For other experimental analyses, blinding was not performed as the same investigators who conducted experiments also performed data acquisition and analysis.

Reporting for specific materials, systems and methods

We require information from authors about some types of materials, experimental systems and methods used in many studies. Here, indicate whether each material, system or method listed is relevant to your study. If you are not sure if a list item applies to your research, read the appropriate section before selecting a response.

Materials & experimental systems

n/a	Involved in the study
☐	☒ Antibodies
☐	☒ Eukaryotic cell lines
☒	☐ Palaeontology and archaeology
☐	☒ Animals and other organisms
☒	☐ Clinical data
☒	☐ Dual use research of concern
☒	☐ Plants

Methods

n/a	Involved in the study
☒	☐ ChIP-seq
☐	☒ Flow cytometry
☒	☐ MRI-based neuroimaging

Antibodies

Antibodies used

DDX6 (1:5000, Novus Biologicals, Cat#NB200-192)
Argonaute-2 (1:1000, Cat#C34C6; Cell Signaling Technologies)
HRP-rabbit anti-human/mouse β-Actin (1:3000, Cat#5125, Cell Signaling)
Goat, anti-rabbit-HRP-conjugated (Cat#31460, Thermo Fisher Scientific, 1:2,000 dilution)
Rabbit, anti-mouse-HRP-conjugated (Cat#31450, Thermo Fisher Scientific, 1:2,000 dilution)
EDC4 (1:50, Cat#ab72408, Abcam)
GFP (1:400, Cat#GFP-1020, Aves Lab)
KLF17 (1:100, HPA024629, SIGMA),
NANOG (D73G4) (Cat#4903, 1:300, Cell Signaling)
BRACHYURY (T) (1:50, Cat#AF2085, R&D Systems)
FOXA2 (1:400, Cat#8186, Cell Signaling)
SOX1 (1:200, Cat#AF3369, R&D Systems)
bIII-TUBULIN (TUJ1) (1:200, Cat# 801213, Biolegend)
METTL3 (1:100, Cat# 15073-1-AP, Proteintech)
Alexa 647-anti-mouse/human Ki-67 (1:300 dilution) (11F6) (Cat# 151206, BioLegend)
Alexa Fluor 488-conjugated donkey anti-chicken IgG (H+L) (1:500, Cat# A78948, Life Technologies)
Anti-N6-Methyladenosine (m6A) antibody, Rabbit monoclonal (1:3000 dilution) (Cat# SAB5600251, Sigma)
Phalloidin-iFluor 594 Reagent (1:500 dilution) (Abcam, Cat# ab176757)

Validation

DDX6 (1:5000, Novus Biologicals, Cat#NB200-192)
https://www.novusbio.com/products/ddx6-antibody_nb200-192?srsltid=AfmBOooTIBwZKIu3iaVn8xQht0JfJX-M5VzEonp_Vp9tvbFO5B_QrAP2#datasheet

Rabbit anti-Argonaute-2 (1:1000, Cat#C34C6; Cell Signaling Technologies)
https://www.cellsignal.com/products/primary-antibodies/argonaute-2-c34c6-rabbit-mab/2897?srsltid=AfmBOoo0ZxvjsLCA2JgnIdfy-aORpK-FnJOCx9a5OVETtwBPg4WPAZ17

HRP-rabbit anti-human/mouse β-Actin (1:3000, Cat#5125, Cell Signaling)
https://www.cellsignal.com/products/antibody-conjugates/b-actin-13e5-rabbit-mab-hrp-conjugate/5125?srsltid=AfmBOopPVXL5QcGS16V9YcZvdcItwAEHpVIspnMCnE1-9kyLfFj-Ip2m

Goat, anti-rabbit-HRP-conjugated (Cat#31460, Thermo Fisher Scientific, 1:2,000 dilution)
https://www.thermofisher.com/antibody/product/Goat-anti-Rabbit-IgG-H-L-Secondary-Antibody-Polyclonal/31460

Rabbit, anti-mouse-HRP-conjugated (Cat#31450, Thermo Fisher Scientific, 1:2,000 dilution)
https://www.thermofisher.com/antibody/product/Rabbit-anti-Mouse-IgG-H-L-Secondary-Antibody-Polyclonal/31450

EDC4 (1:50, Cat#ab72408, Abcam)
https://www.abcam.com/en-us/products/primary-antibodies/edc4-antibody-ab72408?srsltid=AfmBOor3ihbPjqa7F9JrZ4ekr0yWt6QRLNwaXlegUXuuCgws1dCAdc3q

GFP (1:400, Cat#GFP-1020, Aves Lab)
https://www.antibodiesinc.com/products/anti-green-fluorescent-protein-antibody-gfp?srsltid=AfmBOopWugN9Bv1fKQxd8GbgIja5-hmc7JOnfWEGAwahE70ZghnXTHLh

KLF17 (1:100, HPA024629, SIGMA)
https://www.sigmaaldrich.com/US/en/product/sigma/hpa024629?srsltid=AfmBOooU1G4wVwStIBLNI0rD0scZcEvlbxud2G8cXAhznf140rye-GqU

NANOG (D73G4) (Cat#4903, 1:300, Cell Signaling)
https://www.cellsignal.com/products/primary-antibodies/nanog-d73g4-xp-rabbit-mab/4903?srsltid=AfmBOoqOjiMq6jo38yf5mHL_szfvSpLp3b6CvO_nQvtdVESMiXBkKId7

BRACHYURY (T) (1:50, Cat#AF2085, R&D Systems)
https://www.rndsystems.com/products/human-mouse-brachyury-antibody_af2085

FOXA2 (1:400, Cat#8186, Cell Signaling)

https://www.cellsignal.com/products/primary-antibodies/foxa2-hnf3b-d56d6-xp-rabbit-mab/8186?srsltid=AfmBOoqtNwFVBDMsXRRR1x353KzaSz7z6bQJVtwh2OhbYCCZt1hC7Ftf

SOX1 (1:200, Cat#AF3369, R&D Systems)
https://www.rndsystems.com/products/human-mouse-rat-sox1-antibody_af3369

βIII-TUBULIN (TUJ1) (1:200, Cat# 801213, Biolegend)
https://www.biolegend.com/de-de/products/purified-anti-tubulin-beta-3-tubb3-antibody-11580?GroupID=GROUP686

METTL3 (1:100, Cat# 15073-1-AP, Proteintech)
https://www.ptglab.com/products/METTL3-Antibody-15073-1-AP.htm?srsltid=AfmBOooUR6mjmoAjC6cOqsEl-0NkORzOf6uoZKC5rU_RsBU6sYjtG2SK

Alexa 647-anti-mouse/human Ki-67 (1:300 dilution) (11F6) (Cat# 151206, BioLegend)
https://www.biolegend.com/de-de/products/alexa-fluor-647-anti-mouse-human-ki-67-antibody-12890

Alexa Fluor 488-conjugated donkey anti-chicken IgG (H+L) (1:500, Cat# A78948, Life Technologies)
https://www.thermofisher.com/antibody/product/Donkey-anti-Chicken-IgY-H-L-Highly-Cross-Adsorbed-Secondary-Antibody-Polyclonal/A78948

Anti-N6-Methyladenosine (m6A) antibody, Rabbit monoclonal (1:3000 dilution) (Cat# SAB5600251, Sigma)
https://www.sigmaaldrich.com/US/en/product/sigma/sab5600251?srsltid=AfmBOoq3ic2rMs7HT0gP-kmYmuwH3jfVJ6Hx4yQ84RYFSjiSI5oImVvB

Phalloidin-iFluor 594 Reagent (1:500 dilution) (Abcam, Cat# ab176757)
https://www.abcam.com/en-us/products/reagents/phalloidin-ifluor-594-reagent-ab176757?srsltid=AfmBOopajLbDdiRcgztSRAFAuE9foGp92dXSbPt2CvoH8hkbgcxh5a4I

Eukaryotic cell lines

Policy information about cell lines and Sex and Gender in Research

Cell line source(s)	UCLA-4 human ES cells (female) were obtained from the UCLA Stem Cell Core. Derivatives of the endoderm, mesoderm and ectoderm lineages were derived from UCLA-4 cells cultured in primed conditions. WIBR3 TETOFF METTL3 cells (female) were obtained from Dr. Jacob Hanna's lab (Weizmann Institute). Chicken ES cells (male and female) were obtained from Dr. Q. Ying's lab (USC). KH2 mouse ES cells (male) were originally obtained from Dr. Rudolf Jaenisch's lab (Whitehead Institute). HEK293T cells ATCC (Cat# CRL3216) were purchased from ATCC. Mouse embryonic fibroblasts were derived from OKSM mouse E13.5 embryos (female and male) as detailed in the methods section. The BTAG hiPSC cell line (male) was obtained from Dr. Mitinori Saitou's lab (ASHBI, Japan). Gen1C hiPSC (male) were obtained from the lab of Dr. Bruce Conklin (UCSF).
Authentication	HEK293T cells were authenticated by ATCC. The other cell lines were authenticated by their donating labs and in prior publications.
Mycoplasma contamination	Cells were confirmed negative for mycoplasma contamination weekly using a Mycoplasma PCR Detection Kit (Applied Biological Materials).
Commonly misidentified lines (See ICLAC register)	No commonly misidentified cell lines were used in this study.

Animals and other research organisms

Policy information about studies involving animals; ARRIVE guidelines recommended for reporting animal research, and Sex and Gender in Research

Laboratory animals	CD1 wild type and OKSM mice (Charles River strain # 022 and Jax #011001) were used as to derive mouse embryonic fibroblasts. All mice used in this study were 8-12 weeks old, and embryos were collected from timed mating at embryonic day 13.5 (E13.5). Mice were maintained under a standard 12 h/12 h light/dark cycle, at ambient temperature and humidity.
Wild animals	Study did not involve wild animals.
Reporting on sex	Both female and male mice were used to derive mouse embryonic fibroblasts (MEFs). Since the experiments were conducted in vitro with isolated cells rather than with intact animals, the sex of the source animals was not a relevant biological variable for the outcomes measured.
Field-collected samples	Study did not involve samples collected from the field.
Ethics oversight	All mice were maintained in specific pathogen-free (SPF) animal facilities, approved, and overseen by the Institutional Animal Care and Use Committee (IACUC) of Baylor College of Medicine (IACUC #AN-8464).

Note that full information on the approval of the study protocol must also be provided in the manuscript.

Flow Cytometry

Plots

Confirm that:

☒ The axis labels state the marker and fluorochrome used (e.g. CD4-FITC).

☒ The axis scales are clearly visible. Include numbers along axes only for bottom left plot of group (a 'group' is an analysis of identical markers).

☒ All plots are contour plots with outliers or pseudocolor plots.

☒ A numerical value for number of cells or percentage (with statistics) is provided.

Methodology

Sample preparation	P-body purification was performed as previously described. In brief, LSM14A-GFP-expressing cells were lysed for 20 min on ice in lysis buffer (50 mM Tris, pH 7.4, 1 mM EDTA, 150 mM NaCl, 0.2% Triton-X-100) supplemented with 65 U ml−1 RNaseOut ribonuclease inhibitor (Promega) and EDTA-free protease inhibitor cocktail (Roche Diagnostics). Lysates were centrifuged at 200g for 5 min at 4 °C to remove nuclei. Residual DNA was removed by incubation in the presence of 10 mM MgSO4, 1 mM CaCl2 and 4 U ml−1 of RQ1 DNase (Promega) for 30 min at room temperature. Following centrifugation at 10,000g for 7 min at 4 °C, pellets were resuspended in 40 µl lysis buffer containing 80 U RNaseOut (Promega) to generate the cytoplasmic fraction. From this fraction, P-bodies were sorted on a FACSAria.<br><br>To detect expression of fluorescent reporter proteins, DAPI or DRAQ7 was first added where appropriate to mark dead cells.
Instrument	Samples were acquired on LSR-II, LSR-Fortessa, FACSCanto II (BD Biosciences), or sorted on a FACSAria or Influx (BD Biosciences). All flow cytometry data were analyzed on FlowJo 10.8.2 (BD Biosciences).
Software	All data were acquired on flow cytometers or sorters using FACSDiva, then analyzed using FlowJo 10.8.2 (BD Biosciences).
Cell population abundance	Post-sort purity of sorted samples was > 95%, and was determined by analyzing the sample on the same instrument immediately after sorting, using the same gates set for sorting.
Gating strategy	Cells were first gated to exclude dead cells, doublets, and debris on the basis of FSC and SSC. Viability dyes (i.e. DAPI or DRAQ7) were used to further exclude dead cells. Cells were therafter gated based on reporter expression. Gates indicating positive and negative are based on isotype controls and single color controls.

☒ Tick this box to confirm that a figure exemplifying the gating strategy is provided in the Supplementary Information.
