# Supplementary material for: Selective RNA sequestration in biomolecular condensates directs cell fate transitions
Source: Nat Biotechnol. 2025 Oct 28;44(9):1553–68. doi: 10.1038/s41587-025-02853-z (PMC12728835; doi:10.1038/s41587-025-02853-z)

Whole membrane images for DDX6 degron western

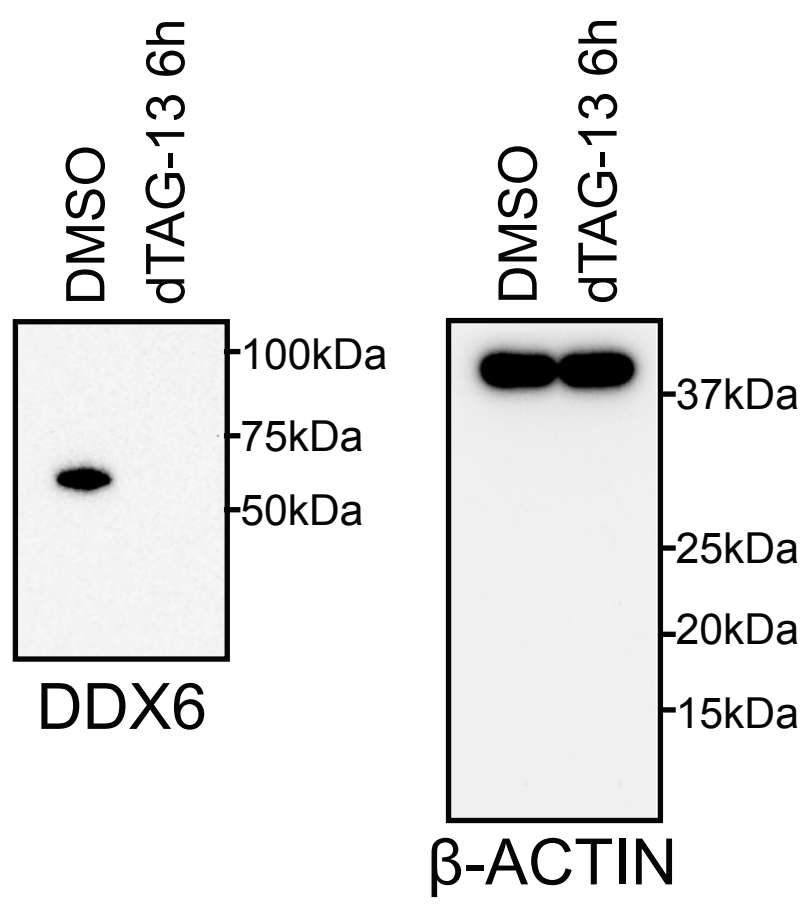

Supplement: Supplementary file 4 [file 41587_2025_2853_MOESM7_ESM.pdf]
